# Clove-mediated green synthesis of silver nanoparticles and *in vitro* investigation of their antimicrobial, antioxidant, and anticancer potential

**DOI:** 10.3389/fmicb.2026.1860045

**Published:** 2026-07-28

**Authors:** Eman Jassim Mohammed, Ali R. Laftah, Salem S. Salem, Eslam Abdelhakim Seyam, Ahmed Gamal Al-bakarey, Yosra Modafer, Fady Sayed Youssef, Ahmed E. M. Abdelaziz, Mahmoud M. Al-Habibi, Mohamed Khedr, Mohammad Alfaifi, Ali A. Shati, Ahmed M. Hussein, Alsayed E. Mekky

**Affiliations:** 1 Department of Microbiology, College of Science, Mustansiriyah University, Baghdad, Iraq; 2 Department of Botany and Microbiology, Faculty of Science, Al–Azhar University, Cairo, Egypt; 3 Department of Insurance and Risk Management, College of Business, Imam Mohammad Ibn Saud Islamic University (IMSIU), Riyadh, Saudi Arabia; 4 Department of Chemistry, Faculty of Science, Al–Azhar University, Cairo, Egypt; 5 Department of Biology, College of Science, Jazan University, Jazan, Saudi Arabia; 6 Department of Pharmacology, Faculty of Veterinary Medicine, Cairo University, Giza, Egypt; 7 Department of Botany and Microbiology, Faculty of Science, Port-Said University, Port-Said, Egypt; 8 Microbiology and Immunology Department, Faculty of Pharmacy (Boys), Al-Azhar University, Cairo, Egypt; 9 Central Labs, King Khalid University, Alqura’a, Abha, Saudia Arabia; 10 Biology Department, Faculty of Science, King Khalid University, Abha, Saudi Arabia; 11 Tissue Culture and Cancer Biology Research Laboratory, King Khalid University, Abha, Saudi Arabia; 12 Department of Pharmaceutical Sciences, Division of Pharmacology and Toxicology, University of Vienna, Vienna, Austria

**Keywords:** antibacterial activity, Coxsackievirus B4, hepatitis A virus, herpes simplex virus type 1, silver nanoparticles, *Syzygium aromaticum*

## Abstract

**Introduction:**

Green synthesis of silver nanoparticles (AgNPs) using plant extracts offers an eco-friendly and sustainable strategy for developing multifunctional nanomaterials with biomedical applications. This study aimed to biosynthesize AgNPs using *Syzygium aromaticum* (clove) extract and evaluate their physicochemical characteristics and antioxidant, anti-inflammatory, antiviral, anticancer, and antibacterial activities.

**Methods:**

AgNPs were biosynthesized using clove extract and characterized by Ultraviolet–Visible Spectroscopy (UV–Vis), X-ray Diffraction (XRD), Transmission Electron Microscopy (TEM), and Energy-Dispersive X-ray Spectroscopy (EDX). Antioxidant activity was determined by IC₅₀ analysis, while anti-inflammatory potential was assessed using hemolysis protection and human red blood cell (HRBC) membrane stabilization assays. Antiviral activity was evaluated against hepatitis A virus (HAV), Coxsackievirus B4 (COXB4), and herpes simplex virus type 1 (HSV-1). Cytotoxicity was assessed in human colorectal carcinoma (CaCO₂) and normal lung fibroblast (Wi-38) cells. Antibacterial activity was tested against multidrug-resistant (MDR) bacterial strains, and quantitative molecular analyses were performed to determine the expression of bacterial virulence genes and cancer-related genes. All reported values represent the mean of three independent experiments.

**Results:**

The synthesized AgNPs were predominantly spherical, well dispersed, and had an average particle size of approximately 9.5 nm. XRD confirmed a crystalline face-centered cubic (fcc) structure, while UV–Vis analysis showed a characteristic surface plasmon resonance peak at 437 nm, confirming successful nanoparticle formation. The AgNPs exhibited strong antioxidant activity with an IC₅₀ of approximately 13.5 μg/mL and demonstrated significant anti-inflammatory activity through membrane stabilization and hemolysis protection. Antiviral assays showed marked inhibition of HAV, COXB4, and HSV-1 at 250 μg/mL, with the highest inhibition observed against HAV (74%). Cytotoxicity studies revealed selective anticancer activity, with an IC₅₀ of approximately 137 μg/mL against CaCO₂ cells compared with 387 μg/mL for Wi-38 cells. Broad-spectrum antibacterial activity was observed against MDR *Staphylococcus aureus* (ATCC 27217), *Staphylococcus haemolyticus* (ATCC 29970), *Escherichia coli* (BAA-197), and *Klebsiella pneumoniae* (BAA-1705). Molecular analyses demonstrated significant suppression of bacterial virulence genes, including luxS in *E. coli* (70%), *fnbA*-a and *cna* in *S. aureus* (74%), *fnbA*-h in *S. haemolyticus* (77%), and *rmpA* in *K. pneumoniae* (81%) at AgNP concentrations of 10 μg/mL for Gram-positive and 12.5 μg/mL for Gram-negative strains. In CaCO₂ cells, AgNP treatment significantly downregulated c-MYC (0.49-fold), K-RAS (0.67-fold), and BCL2 (0.78-fold; *p* < 0.01), while significantly upregulating BAX (1.74-fold).

**Discussion:**

Clove-mediated AgNPs demonstrated potent multifunctional biological activities, including antioxidant, anti-inflammatory, antiviral, selective anticancer, and antibacterial effects, accompanied by modulation of cancer-associated and bacterial virulence genes. These findings highlight the therapeutic potential of biosynthesized AgNPs as promising candidates for the development of novel antimicrobial and anticancer nanomedicines.

## Introduction

Clove is widely recognized for its pain-relieving properties and is rich in diverse bioactive constituents, including methyl salicylate, vanillin, gallotannic acid, *β*-caryophyllene, tannins, crategolic acid, and acetyl eugenol. It also contains flavonoids such as rhamnetin, kaempferol, eugenin, and augmentin, in addition to several sesquiterpenes and triterpenoids, including oleanolic acid, stigmasterol, and campesterol (Mekky et al., 2025a, 2025b). Traditionally, clove has been used to relieve dental pain and gastrointestinal disorders. Clove essential oil acts as a carminative by enhancing gastric acidity and peristalsis and exhibits anti-parasitic, antibacterial, and antifungal activities. For centuries, cloves have been employed as natural remedies for intestinal worms, diarrhea, and related digestive conditions (Mohammad et al., 2026).

Clove buds have also been reported to function as natural biosorbents capable of binding heavy metals. The essential oil is predominantly composed of aromatic compounds (approximately 72–90%), contributing to its anesthetic and antiseptic properties (Tariq et al., 2025). Among its constituents, eugenol is considered the principal bioactive molecule and has been extensively investigated for its antioxidant, antiviral, anti-inflammatory, insecticidal, and photoprotective effects (Mekky et al., 2024a). The abundance of phenolic and terpenoid compounds in clove makes it particularly suitable for nanoparticle synthesis, as these molecules can act as reducing, stabilizing, and capping agents during metal ion transformation (Elbagory et al., 2022). In recent years, plant-derived phytochemicals have attracted significant attention as eco-friendly mediators in the green synthesis of metallic nanoparticles. Such approaches enhance biocompatibility and confer functional surface chemistry to the resulting nanomaterials. Compared to conventional chemical and physical synthesis methods, biosynthetic routes rely on non-toxic reducing agents and eliminate the need for hazardous stabilizers, offering a sustainable alternative for nanoparticle fabrication (Abdelrazik et al., 2023; Hamed et al., 2020). Several studies have reported potent antibacterial activity of metal oxide nanoparticles, including zinc oxide (ZnO), titanium dioxide (TiO₂), and silver oxide (Ag₂O), particularly against multidrug-resistant (MDR) bacterial strains (Liao et al., 2019; Mba and Nweze, 2020).

Metal-based nanoparticles exert antimicrobial activity through multiple cellular and molecular mechanisms. These include interaction with bacterial DNA, disruption of replication processes, protein denaturation, membrane destabilization, increased permeability, and generation of reactive oxygen species (ROS), collectively leading to impaired cellular homeostasis (Abdelrazik et al., 2023). Among various nanomaterials, silver nanoparticles (AgNPs) have received considerable attention due to their broad-spectrum antimicrobial activity and relatively lower cytotoxicity toward mammalian cells at controlled concentrations (Mekky et al., 2024b). Their high surface area-to-volume ratio enhances physicochemical interaction with microbial cells, contributing to improved antimicrobial efficacy (Stabryla et al., 2018). With the global rise of MDR infections, nanotechnology-based antimicrobial strategies have emerged as promising complementary tools (Mekky et al., 2024b; Mba and Nweze, 2021; Liu et al., 2025). Although AgNPs can be synthesized via chemical, physical, and mechanical methods, many of these techniques involve toxic reagents or high energy consumption. In contrast, biological synthesis using plant extracts provides a safer, cost-effective, and environmentally sustainable approach (Ahmed et al., 2022; Jeevanandam et al., 2022). The phytochemical composition of plant extracts not only mediates nanoparticle formation but may also synergistically enhance biological performance.

Recent advances in nanotherapeutic research have increasingly emphasized mechanism-oriented and multimodal strategies. Contemporary platforms integrate externally triggered activation systems, such as ultrasound-assisted or semiconductor-sensitized nanoparticles, to amplify ROS generation and enhance antimicrobial and antivirulence efficacy (Ding et al., 2026). These approaches aim not only to induce microbial cell death but also to interfere with virulence pathways, quorum sensing networks, and biofilm formation, thereby potentially reducing resistance development (Juszczuk-Kubiak, 2024). While such sophisticated systems demonstrate enhanced precision, their complexity, cost, and scalability may limit widespread application. Within this evolving landscape, green-synthesized AgNPs derived from medicinal plant extracts represent a simpler yet scientifically relevant platform that warrants systematic evaluation in relation to current state-of-the-art strategies (Nagime et al., 2025).

Beyond antimicrobial properties, AgNPs have also been investigated for their broader biological interactions. Experimental studies suggest that AgNPs may influence intracellular signaling pathways such as mitogen-activated protein kinase (MAPK), nuclear factor kappa B (NF-κB), and epidermal growth factor receptor (EGFR), which are associated with inflammation, cell proliferation, and tumor progression (Hassan et al., 2024; Takáč et al., 2023; Thiruvengadam et al., 2023). Additionally, AgNPs have been explored as photothermal and photodynamic agents, as well as nanocarriers for targeted drug delivery and adjuncts to conventional therapeutic modalities (Hassan et al., 2024; Melo et al., 2025). However, careful evaluation of their cytotoxic profile and mechanistic interactions remains essential before considering translational applications (Jain et al., 2021; Kowalczyk et al., 2021).

Although several studies have reported the green synthesis of AgNPs using clove extract and investigated selected biological activities, most previous reports focused on individual applications or a limited range of biological evaluations (Andleeb, 2020). In contrast, the present work provides a comprehensive assessment of clove-mediated AgNPs by integrating physicochemical characterization with the evaluation of multiple biological activities, including antimicrobial activity against multidrug-resistant pathogens, antioxidant, anti-inflammatory, antiviral, and anticancer effects. This integrated approach contributes to a broader understanding of the biomedical potential of clove-derived AgNPs and supports their future development as multifunctional nanomaterials.

Despite promising developments in advanced multimodal nanotherapeutic systems, foundational *in vitro* studies remain critical for establishing biological performance, safety margins, and mechanistic indications of newly synthesized nanoparticles. In this context, plant-mediated green synthesis offers a sustainable platform for generating AgNPs suitable for preliminary biological screening and further optimization. Therefore, the present study aimed to synthesize silver nanoparticles using clove extract through a green approach and to characterize their physicochemical properties. The *in vitro* biological activities of the synthesized AgNPs were systematically evaluated, including antimicrobial activity against MDR strains, antioxidant capacity, anti-inflammatory effects, antiviral activity, and cytotoxicity against cancer cell lines, in order to provide a comprehensive experimental assessment within the framework of sustainable nanomaterial development.

## Materials and methods

### Plant samples collection

Dried flower buds of *Syzygium aromaticum* L. (clove) were sourced from a local marketplace in Cairo, Egypt. The plant material was taxonomically authenticated by Prof. Dr. Abdo Hamed Marai from Al-Azhar University, with further verification by the Department of Botany and Microbiology, Faculty of Science, Al-Azhar University. Although a formal voucher specimen number was not issued, the extract composition and phytochemical consistency were confirmed through GC–MS analysis, ensuring reproducibility and traceability. Prior to use, the cloves were rinsed thoroughly with double-distilled water to remove surface impurities, dried in an oven at 55 °C for 48 h, ground into a fine powder, and sieved for uniform particle size. The resulting biosorbent powder was stored in airtight containers under dry conditions for subsequent experiments.

### queous extract preparation from clove

For extract preparation, 25 grams of finely powdered clove were combined with 500 mL of distilled water and stirred thoroughly to ensure proper mixing. The mixture was allowed to stand at room temperature overnight to facilitate maximum extraction of bioactive compounds. It was then subjected to gentle heating at 60 °C for 20 min. Once cooled to room temperature, the mixture was filtered through Whatman No. 1 filter paper (110 mm diameter) to remove solid residues. The obtained aqueous extract was collected and stored at 4 °C until further use in subsequent experiments (Mekky et al., 2024a; Behera et al., 2024).

### Plant extract process and AgNPs biosynthesis

Clove buds obtained from *S. aromaticum* were oven-dried to constant weight, manually cleaned, and ground into a fine powder. Fifty grams of the dried powder were extracted using a methanol–distilled water mixture (50:50, v/v). The mixture was sonicated to enhance extraction efficiency and then filtered through Whatman No. 1 filter paper. The aqueous fraction was freeze-dried and stored at 4 °C, while the methanolic fraction was concentrated under reduced pressure using a rotary evaporator to remove the solvent (Alsamman et al., 2023). For the green synthesis of AgNPs, a slightly modified protocol based on the method described by Oves et al. (2022) was followed. Briefly, 5 mL of the prepared clove extract was mixed with 50 mL of 1 mM AgNO₃ solution in a 250 mL Erlenmeyer flask. The reaction mixture was stirred vigorously at 90 °C for 2 min. A rapid color change from pale yellow to brownish-yellow within approximately 5 min indicated the reduction of Ag^+^ ions and formation of AgNPs. The synthesized nanoparticles were collected by centrifugation at 10,000 rpm for 10 min and washed three times with distilled water to remove residual impurities. The purified AgNPs were stored in dark, airtight containers under controlled conditions for further characterization and applications.

### AgNPs characterization

The successful formation of AgNPs was preliminarily indicated by a visible color change, reflecting the reduction of Ag^+^ ions to metallic Ag^0^ using extract of *S. aromaticum* as a natural reducing and stabilizing agent. To comprehensively evaluate their physicochemical properties, multiple analytical techniques were employed. UV–Visible spectroscopy was performed using a JASCO V-730 spectrophotometer, and absorption spectra were recorded over a wavelength range of 200–700 nm with a resolution of approximately 0.2 nm. The appearance of a characteristic surface plasmon resonance (SPR) band confirmed nanoparticle formation. Transmission electron microscopy (TEM) analysis was conducted using a JEOL 1200EX microscope operated at an accelerating voltage of 120 kV. A drop of the nanoparticle suspension was deposited onto a carbon-coated copper grid and allowed to dry prior to imaging to determine particle morphology and size distribution. X-ray diffraction (XRD) measurements were carried out using a Rigaku Ultima IV diffractometer with Cu Kα radiation (*λ* = 1.54060 Å). Diffraction data were collected over a 2*θ* range of 10°–80°, employing a scanning rate of 2° min^−1^ and a step size of 0.02°. The average crystallite size (D) was estimated using the Debye–Scherrer equation:


D=Kλ/βcosθ,

where D represents the crystallite size, K is the shape factor (0.9), λ is the X-ray wavelength (0.154 nm), *β* is the full width at half maximum (FWHM), and θ is the Bragg diffraction angle. Fourier transform infrared (FTIR) spectroscopy was performed using a JASCO-6700 instrument within the spectral range of 400–4,000 cm^−1^ to identify functional groups involved in nanoparticle reduction and stabilization. For sample preparation, 300 μL of the AgNP suspension was mixed with 10 mg of KBr, oven-dried, and compressed into pellets prior to analysis. The hydrodynamic particle size distribution, polydispersity index (PDI), and zeta potential of the biosynthesized AgNPs were measured using a SZ-100 nanoparticle analyzer (HORIBA Scientific, Kyoto, Japan).

### Antioxidant potential of AgNPs

The antioxidant potential of the synthesized silver nanoparticles was assessed using the DPPH (1,1-diphenyl-2-picrylhydrazyl) free radical scavenging assay, which is a standard approach for evaluating the ability of compounds to neutralize free radicals. A 0.1 mM DPPH solution was freshly prepared in ethanol. For the assay, 3 mL of AgNP suspensions at different concentrations (ranging from 0.003 to 1 mg/mL) were individually combined with 1 mL of the DPPH solution. Each mixture was briefly vortexed and then kept in the dark at room temperature for 30 min to allow the reaction to proceed. After the incubation period, absorbance was measured at 517 nm using a Milton Roy UV–Vis spectrophotometer. Ascorbic acid served as the standard antioxidant for benchmarking the scavenging efficiency (Bhakya et al., 2016)


$$\begin{array}{l} \text{inhibition percentage or DPPH reduction potential}\\ =\mathrm{A0}-A1/\mathrm{A0}\times 100. \end{array}$$

where A1: test or standard sample absorbance, and A0: control response reading.

### Anti-inflammatory action of AgNPs

#### Hypotonicity-induced hemolysis assay

About 3 mL of blood was aseptically collected from healthy volunteers with informed written consent into tubes containing heparin as an anticoagulant. The study was conducted in accordance with the ethical standards of Al-Azhar University, Assiut, Egypt (Approval No. 16-2025, dated May 2025), and in compliance with the Declaration of Helsinki. Multiple independent donor samples were used to account for variability. The samples were centrifuged at 3000 rpm for 10 min to separate the red blood cells (RBCs). The RBC pellet was washed with an equal volume of physiological saline to remove plasma residues, and then resuspended to obtain a 40% (v/v) RBC suspension in isotonic phosphate buffer (10 mM, pH 7.4) (Olasunkanmi and Afuye, 2021).

For the hypotonicity-induced hemolysis assay, plant extracts were dissolved in distilled water to produce a hypotonic medium, and serial dilutions (0.1–1 mg/mL) were prepared in triplicate (5 mL per tube). Parallel isotonic solutions with the same extract concentrations were also prepared. Indomethacin (0.2 mg/mL) served as a positive control, while distilled water functioned as the negative control. A volume of 0.1 mL of the RBC suspension was added to each tube, and the mixtures were incubated at 37 °C for 1 h. Tubes were then centrifuged at 1300 × g for 3 min, and the absorbance of the supernatant was measured at 540 nm to assess hemoglobin release. Hemolysis caused by distilled water was considered as 100%, and the percentage of protection offered by the extracts was calculated using the following equation (Olasunkanmi and Afuye, 2021):


$$\begin{array}{l} \text{Hemolysis inhibition rate}\;(\%)=\\ 1-((\mathrm{OD2}-\mathrm{OD1})/(\mathrm{OD3}-\mathrm{OD1}))\times 100. \end{array}$$

Where: OD1 = absorbance of the sample in isotonic solution; OD2 = absorbance of the sample in hypotonic solution and OD3 = absorbance of the control sample in hypotonic solution.

#### Antibacterial activity of NPs

The antibacterial efficacy of the biosynthesized Ag NPs was tested against four MDR bacterial strains, comprising two Gram-positive species (*S. aureus* ATCC 27217 and *S. haemolyticus* ATCC 29970) and two Gram-negative species (*E. coli* BAA-197 and *K. pneumoniae* BAA-1705. Antibacterial efficacy was assessed using the agar well diffusion method, following the procedure described by Alsamman et al. (2023).

### NPs as anti-virulence bacterial factors

#### RNA isolation and cDNA synthesis

##### RNA isolation

According to pamphlet’ constructions, total RNAs of 48-h fermentation of four bacterial strains grown in a Lauria Bertani broth using Trizol (Gibcol). Using the Advantage RT-PCR Kit (Clontech, Alo Alto, CA, USA), cDNA of the positive sense strand was synthesized.

#### qRT-PCR analysis of virulence genes

Quantitative PCR (qRT-PCR) was performed to evaluate the relative expression of selected virulence-associated genes, including *fnbA* and *Cna* from *S. aureus, rmpA* from *Klebsiella pneumoniae*, and *luxS* from *E. coli*. The *RecA* gene served as the reference housekeeping gene. Specific primers used for amplification are listed in Table 1 and were designed using the NCBI Primer-BLAST tool. Total RNA was extracted following standard protocols and reverse transcribed into cDNA using a commercial cDNA synthesis kit according to the manufacturer’s instructions (Wong and Medrano, 2005). PCR amplification was carried out with gene-specific primers under optimized conditions, ensuring that reactions remained within the linear range. PCR products were separated by agarose gel electrophoresis and visualized with an appropriate nucleic acid stain (Bustin, 2005). Band intensities were quantified using densitometric analysis, and relative gene expression levels were compared between samples.

**Table 1:** Specific PCR-primers for amplification and qRT-PCR of virulence genes interest.

Primer	Target gene	Sequence (5′- > 3′)	Tm	GC%	Self 3′ comp	Refetences
FW-primer-1	*fnbA*	AGCGGTAACCAGTCATTCGAG	60	52	2	Mekky et al. (2024b)
RW-primer-1		TTGGCGGCGTTGTATCTTCT	60	50	0	
FW-primer-2		GAAGATACAACGCCGCCAAC	59.9	55	0	
RW-primer-2		AGGTTCTTCTTTTGCGGGTG	58.8	50	0	
FW primer-3	*Cna*	AAGGTGAACAGGTGGGTCAA	59	50	0	
RW primer-3		CACTACTTGTTCCCGCTTCA	57.8	50	1	
FW1RecA	*RecA*	GCCCTAATTGGTCCAGGCG	45	44.3	0	Ginzinger (2002)
RW1 RecA		ACAACGGCGTTCTCTCCTAT	45	44.4	0	
FW2 RecA		ACACAACGTCATTGCAAATGTGA	45	44.3	1	
RW2 RecA		GCCTGGACCAATTAGGGCAT	45	44.3	1	
Fw-Rm	*RmpA*	TGCATGAGCCATCTTTCATCAAC	59.8	43.4	2	This study
RW-Rm		ACTGGGCTACCTCTGCTTCAT	60.9	52.3	2	
FW-Lu	*LuxS*	GCCACACTGGTAGACGTTCA	59.9	55	2	
RW-Lu		ATCCATACCCTGGAGCACCT	60	55	2	

#### Antiviral potential to HSV-1, HAV, and CoxB4

##### Propagation of HSV-1, HAV, and CoxB4 on Vero cells

Herpes simplex virus type 1 (HSV-1), Hepatitis A virus (HAV), and Coxsackievirus B4 (CoxB4) were obtained from the Applied Research Laboratory, VACSERA (Giza, Egypt), and propagated in Vero cells cultured in E-MEM supplemented with 10% FBS and antibiotics at 37 °C with 5% CO₂. Vero cells were seeded in 75-cm^2^ flasks and infected at an MOI of 0.5 after removing the growth medium. Virus adsorption was carried out for 60–90 min with gentle agitation. Fresh medium containing 2% FBS was then added without removing the inoculum. Infected and uninfected (control) cultures were incubated at 37 °C and observed daily for cytopathic effects (CPE). Once CPE reached ~90%, cultures were harvested by three freeze–thaw cycles, aliquoted, and stored at −70 °C (Afshar, 1992; Schmidt, 1995).

#### Antiviral potential of tested AgNPs

The antiviral efficacy of the prepared AgNPs was assessed against HSV-1, HAV, and CoxB4 following the protocol described by Husseiny et al. (2015), with minor modifications. Viral titers were determined in Vero cell lines 24 h after exposure to AgNPs, and antiviral activity was expressed as the reduction in viral titer relative to untreated controls. Briefly, Vero cells were seeded in 96-well plates at a density of 1 × 10^5^ cells/mL and allowed to form monolayers. Cells were pretreated with a previously determined safe concentration of AgNPs (31.25 μL/well) and incubated at 37 °C for 24 h. Untreated wells served as negative controls. After removing the nanoparticle-containing medium, 100 μL of 10-fold serial dilutions of HSV-1, HAV, and CoxB4 (prepared in E-MEM) were added to both treated and untreated wells. Plates were incubated at 37 °C and monitored daily under an inverted microscope for cytopathic effects (CPE). After 7 days, CPE endpoints were recorded and compared between treated and control groups (Steven et al., 2000).

Viral titers were calculated as the 50% cell culture infectious dose (CCID₅₀) using the Reed and Muench (1938) method. Antiviral activity was expressed as the log reduction in viral titer between treated and untreated samples. The percentage antiviral potential was calculated according to the following equation (El-Sheekh et al., 2022):

$\begin{array}{l} \text{Antiviral Potential}\;(\%)=\\ (\log\;\text{virus titer reduction}/\log\;\text{virus seed titer})\times 100. \end{array}$

All titration assays were conducted in triplicate to ensure reproducibility.

#### Antineoplastic activity of Green AgNPs

##### MTT assay

The cytotoxic and metabolic effects of the biosynthesized AgNPs were evaluated using the MTT assay (3-(4,5-dimethylthiazol-2-yl)-2,5-diphenyltetrazolium bromide; Sigma-Aldrich), following established procedures (Elhalik et al., 2024; Kamiloglu et al., 2020). This colorimetric assay measures mitochondrial metabolic activity as an indicator of cell viability. Human colorectal adenocarcinoma (CaCO₂) and normal lung fibroblast (Wi-38) cell lines were cultured to confluence and then treated with varying concentrations of AgNPs for 24–48 h. Untreated cells maintained in DMEM supplemented with 10% fetal bovine serum (FBS) at 37 °C served as controls. After the incubation period, absorbance was measured at 490 nm using a microplate reader. Cell viability (%) was calculated relative to the control according to the standard formula described in the referenced method (Elhalik et al., 2024). All experiments were performed in triplicate, and results were expressed as mean ± standard deviation.

#### qPCR assay

Total RNA was isolated from CaCO2 cell line treated with (137.35 μg/mL AgNPs) using the Trizol reagent (Gibco) according to the protocol provided by the manufacturer. The synthesis of the first-strand cDNA was performed using the Advantage RT-for PCR Kit (Clontech, Palo Alto, CA, USA). Reverse transcription polymerase chain reaction (rt-PCR) was executed in 50 μL reactions using specific primers detailed in Table 1. Each PCR reaction mix included 75 ng of the generated cDNA, 200 mM dNTPs, 0.1 mM of each specific primer, 1.5 mM MgCl2, and 1 U of Taq DNA polymerase (Takara), with the final volume adjusted to 50 μL with sterile water. The PCR amplification followed these conditions: an initial denaturation step at 94 °C for 4 min; 32 cycles of denaturation at 94 °C for 30 s, annealing at 50 °C for 50 s, extension at 71.5 °C for 1 min; and a final extension at 71.5 °C for 5 min. The PCR products were then run on an agarose gel using TAE buffer (40 mM Tris-Acetate, pH 7.6, and 1 mM EDTA). After electrophoresis, the gels were stained with ethidium bromide (0.5 mg/mL), and the bands were visualized under UV light. The lengths of the amplicons were compared to a standard DNA marker (GeneRuler™ 100 bp DNA Ladder, MBI Fermentas, Vilnius, Lithuania) to determine their sizes (Elhalik et al., 2024).

Table DD specific PCRvprimers for oncogenes and apoptosis markers in CaCO2 cell line.

**Table:** 

Gene	Forward primer (5′ → 3′)	Reverse primer (5′ → 3′)	References
MYC (c-MYC)	ACACATCAGCACAACTACG	CGCCTCTTGACATTCTCC	Gheytanchi et al. (2021)
KRAS	CAGTAGACACAAAACAGGCTCAG	TGTCGGATCTCCCTCACCAATG	This study
BCL2	CTTGACAGAGGATCATGCTGTAC	GGATGCTTTATTTCATGAGGC	Sabit et al. (2021)
BAX	GGGCCCACCAGCTCTGA	CCTGCTCGATCCTGGATGA	Lo et al. (2014)
GAPDH	GCCTCGTCCCGTAGACAAAA	GCAACAATCTCCACTTTGCCA	Mekky et al. (2025a, 2025b)

### Real-time PCR amplification conditions

Using the primers shown in Table 1, complementary DNA (cDNA) from control and treated samples was subjected to semiquantitative PCR. 2x Quantitech SYBR® Green RT Mix (Fermentase) containing approximately 12.5 L of the 25 L of the real-time PCR experiment consisting of 1 L of 50 ng cDNA, 1 L of 25 p.m./L from each forward and reverse primer, and 9.25 L of RNase-free water. The centrifuge was used on the samples before they were loaded into the rotor wells. PCR program set for interest genes as starting denaturation for 2 min at 95 °C, then cycles at 95 °C/30 s, annealing at 57.5 °C/30 s, extension step at 72 °C/30 s. On the other hand, the PCR program for 18 S, which is a standard housekeeping gene, was optimized and adjusted as follows: denaturation at 95 °C/2 min, annealing at 44 °C/25 s, and extension at 72 °C/30 s. The Rotor Quality 6,000 gear from Qiagen in the USA was utilized to do the response. The PCR cycle at the threshold cycle (CT) results are present (Elhalik et al., 2024; Abdel Ghany Elrahmany et al., 2023).

### Statistical analysis

All experimental data were statistically analyzed to evaluate significant differences among treatment groups. The effects of varying concentrations of clove-derived AgNPs and different exposure times were assessed using one-way analysis of variance (ANOVA). Statistical calculations were performed using the ANOVA program, and differences were considered statistically significant at *p* < 0.05.

## Results and discussion

### HPLC analysis

The HPLC analysis of clove extract revealed the presence of several phytochemicals belonging to different chemical classes, including phenolic compounds, sesquiterpenes, polyacetylenes, and fatty acids (Table 2). Among the identified compounds were Eugenol, Bicyclo[7.2.0]undec-4-ene, Humulene, Cis-*α*-Bisabolene, Panaxydol, and Palmitic acid. These compounds have been previously reported in the literature as constituents of clove and other medicinal plants and are known to possess various biological properties (Mekky et al., 2024a).

**Table 2:** Key compounds in clove extract and their potential bioactivities.

Compound name	Area %	Chemical class	Molecular formula	Potential bioactivity
Eugenol (Phenol, 2-methoxy-4-(2-propenyl))	5.23	Phenolic compound	C10H12O2	Antimicrobial, antioxidant, anticancer
Bicyclo[7.2.0]undec-4-ene, 4,11,11-trimethyl-8-methylene	31.25	Sesquiterpene hydrocarbon	C15H24	Antimicrobial, moderate antioxidant
Humulene	2.73	Sesquiterpene	C15H24	Antimicrobial, anti-inflammatory
Cis-α-Bisabolene	2.73	Sesquiterpene	C15H24	Antimicrobial, anti-inflammatory
2-Propenoic acid, 3-phenyl	1	Phenolic acid derivative	C9H8O2	Antioxidant, antimicrobial
Panaxydol	1.66	Polyacetylene	C17H24O2	Anticancer, antimicrobial
Palmitic acid/Hexadecanoic acid	3	Saturated fatty acids	C16H32O2	Antimicrobial, membrane-active

Eugenol, one of the major phenolic constituents detected in the extract, has been widely reported to exhibit antioxidant, antimicrobial, and anticancer activities (Aldabaan et al., 2024). In addition to its biological properties, phenolic compounds have been suggested to participate in the reduction and stabilization of metal nanoparticles during green synthesis processes. However, the present study did not directly investigate the specific role of Eugenol in nanoparticle formation or biological activity. Sesquiterpenes identified in the extract, including Bicyclo[7.2.0]undec-4-ene, Humulene, and Cis-*α*-Bisabolene, have been previously associated with antimicrobial and anti-inflammatory activities. Their presence in the extract reflects the complex phytochemical composition of clove and may contribute to the chemical environment involved in nanoparticle biosynthesis. Nevertheless, their individual contribution to the biological activities observed in this study was not experimentally evaluated. Similar mechanisms were reported by Rajivgandhi et al. (2020), who found that sesquiterpene-rich extracts improved the antibacterial potential of green-synthesized nanoparticles. Moreover, their presence may contribute to the biocompatibility of AgNPs on mammalian cell lines, minimizing cytotoxicity to normal cells.

The extract also contained Panaxydol, a polyacetylene compound reported in previous studies to possess antimicrobial and anticancer properties, as well as Palmitic acid, a saturated fatty acid frequently identified in plant-derived extracts. While these compounds have documented biological activities, the current results do not permit direct attribution of the observed antimicrobial, antioxidant, or anticancer effects of the synthesized AgNPs to any specific phytochemical constituent. Fatty acids such as Palmitic acid contribute to membrane-targeted antibacterial activity, improving the interaction of AgNPs with bacterial cell walls. These results are consistent with findings by Shehabeldine et al. (2022), who demonstrated that fatty acid-capped nanoparticles showed higher antibacterial efficacy against MDR strains.

Overall, the HPLC profile demonstrates that the clove extract contains a diverse mixture of bioactive compounds that may participate in nanoparticle synthesis and stabilization. Similar phytochemical compositions have been reported in studies involving plant-mediated synthesis of silver nanoparticles. Previous studies (Husseiny et al., 2015; Govindappa et al., 2021) reported that green-synthesized AgNPs from phenolic-rich plant extracts exhibited smaller particle size, higher surface reactivity, and improved biological activities, which corroborates our findings. The presence of multiple bioactive compounds also supports the observed anticancer effects, consistent with Rajeshkumar et al. (2018), who reported superior cytotoxicity of polyphenol-capped AgNPs on cancer cell lines compared to crude extracts.

### Green synthesis of AgNPs

**Figure 1:**
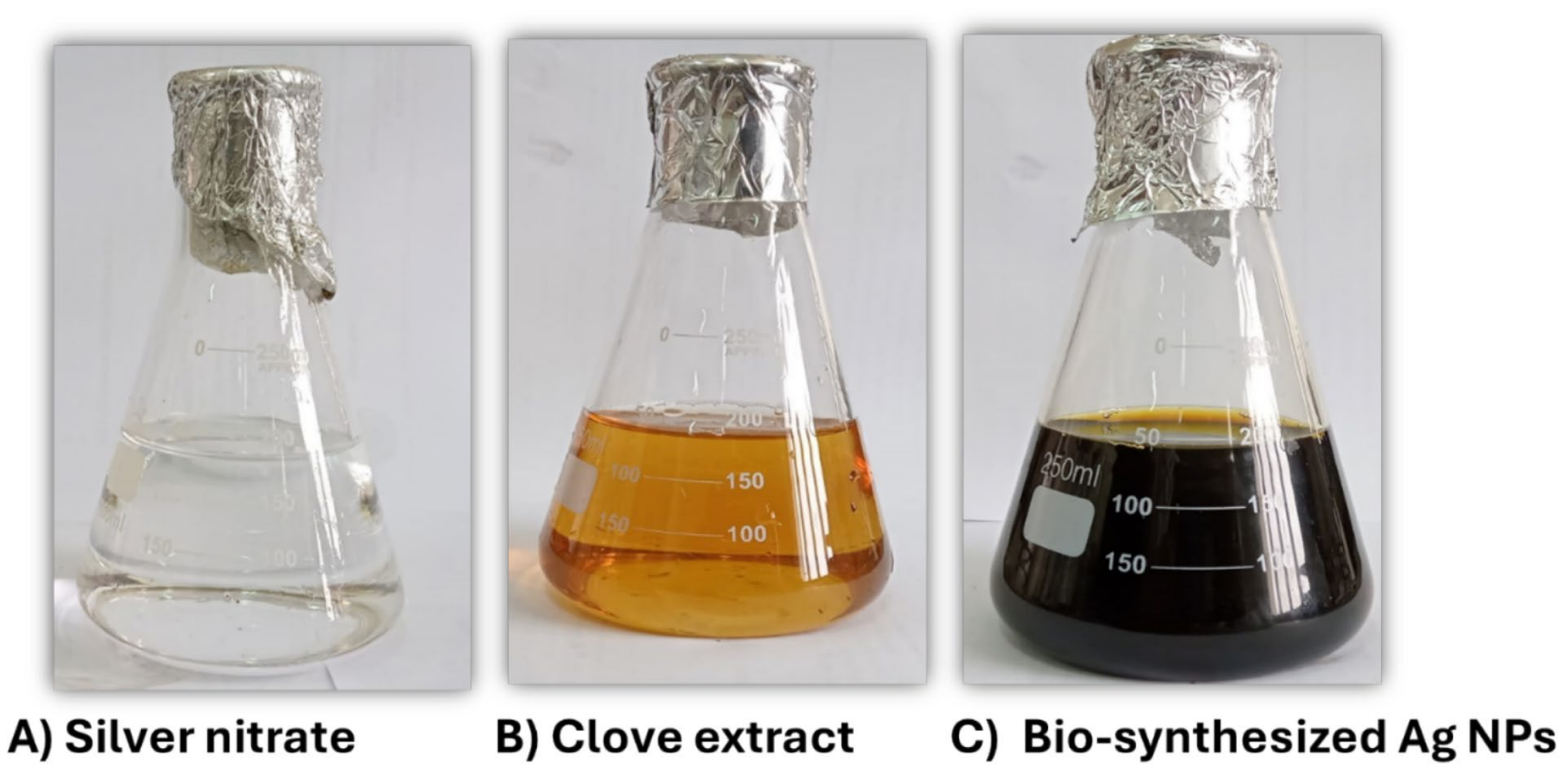
Schematic representation of the green synthesis process: **(A)** silver nitrate (AgNO₃) solution, **(B)** extract of *Syzygium aromaticum* (clove), and **(C)** formation of AgNPs.

Plants are recognized as efficient biofactories for nanoparticle synthesis due to their abundance of phytochemicals capable of reducing and stabilizing metal nanoparticles (Bandeira et al., 2020; Sharma et al., 2023). In the present study, clove extract successfully mediated the reduction of Ag^+^ ions from AgNO_3_ to AgNPs, as evidenced by the gradual color change from colorless to yellow and finally dark brown (Figure 1). This color transformation is characteristic of silver nanoparticle formation and is associated with the excitation of surface plasmon resonance resulting from the reduction of silver ions into metallic nanoparticles (Soni et al., 2021). The successful synthesis of AgNPs can be attributed to the phytochemical constituents of clove extract, particularly phenolic compounds and other reducing metabolites, which may participate in both nanoparticle formation and stabilization. The rapid appearance of the brown coloration suggests efficient reduction kinetics and successful nanoparticle generation. Furthermore, the reproducible formation of nanoparticles across independent batches indicates the reliability of the biosynthesis process.

Preliminary observations indicated consistent nanoparticle formation across multiple batches, supporting reproducibility. The rapid color change also provides an initial indication of reaction kinetics. Appropriate controls, including extract-only and chemically synthesized AgNPs, were considered to distinguish nanoparticle-specific effects from residual phytochemicals in downstream bioassays. Among various green synthesis strategies involving microorganisms (such as fungi, bacteria, actinomycetes, and yeasts), plant-based methods stand out for their simplicity, sustainability, and scalability (Jeevanandam et al., 2022; Dikshit et al., 2021). In this process, clove leaf extract not only reduced Ag^+^ ions but also acted as a capping and stabilizing agent. Similar strategies have been reported in the literature. For instance, Al-Askar et al. (2023) demonstrated the biosynthesis of white ZnO nanoparticles via the reaction between zinc acetate (ZnCH_3_COOH) and an aqueous extract of *Pluchea indica*. Moreover, orange and pomegranate peel extracts, rich in total phenolics, have been shown to effectively reduce Ag^+^ ions to AgNPs (Mostafa et al., 2021). Singh et al. (2021) used *Carissa carandas* leaf extract for the dual role of reducing and capping agents in AgNPs biosynthesis. Additionally, fungal sources such as *Rhizopus oryzae* biomass extract have also been employed for the green synthesis of AgNPs (Saied et al., 2023). Narenkumar et al. (2018) reported the green synthesis of silver nanoparticles using *Azadirachta indica* leaf extract, where the phytochemical and proteinaceous constituents of the extract functioned as both reducing and stabilizing agents during nanoparticle formation. The biosynthesized AgNPs exhibited notable antimicrobial and antibiofilm activities, highlighting the important role of plant-derived biomolecules in enhancing nanoparticle stability and biological efficacy. These findings support the use of plant-mediated synthesis as an eco-friendly approach for producing bioactive silver nanoparticles with promising biomedical applications. However, differences in nanoparticle characteristics may arise from variations in phytochemical composition, extract concentration, and synthesis conditions. Therefore, the properties observed in the present study are likely influenced by the unique composition of the clove extract used as the reducing and capping agent.

### UV–vis spectrophotometry

**Figure 2:**
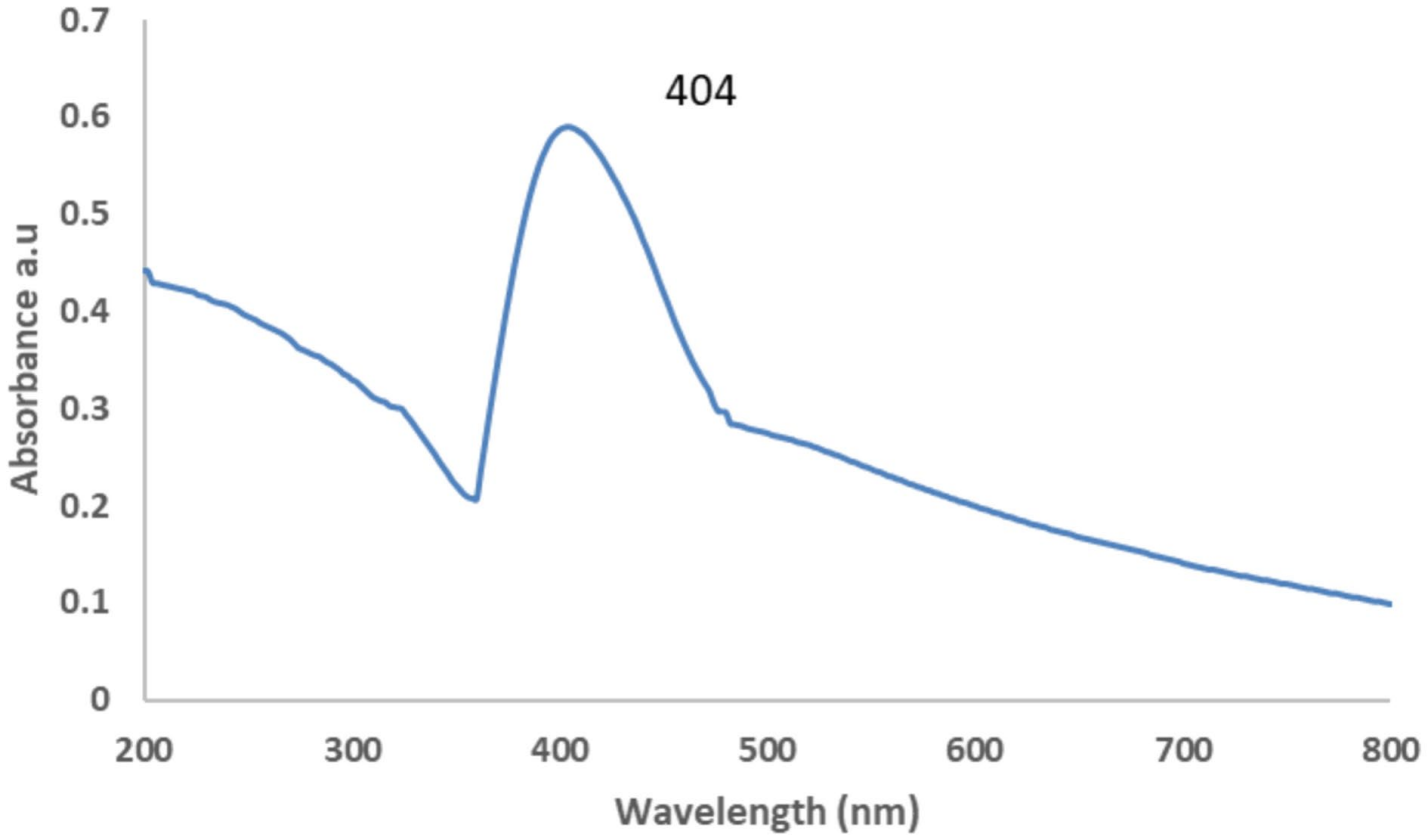
UV–Vis absorption spectrum showing the SPR band of the biosynthesized AgNPs.

The biosynthesized AgNPs were characterized using topographical and physicochemical analyses. As illustrated in Figure 2, the UV–visible absorption spectrum displayed a distinct absorption peak at 404 nm, indicating the formation of AgNPs. A noticeable color change to dark brown was observed during the synthesis, which is typically attributed to the excitation of surface plasmon resonance (SPR) (Saied et al., 2023). The presence of SPR further confirmed the successful reduction of Ag^+^ ions to Ag^0^ nanoparticles, with the characteristic absorption peak at 404 nm falling within the expected SPR range for AgNPs (380–450 nm) (Chinnaiah et al., 2022; Iram et al., 2021; Sarkar et al., 2024). The optical properties of AgNPs are strongly influenced by particle size, shape, and aggregation state. Generally, an increase in particle size or concentration may lead to a red shift in the SPR band, whereas smaller particles often exhibit blue-shifted absorption peaks (Elgendy et al., 2022). Uma Maheshwari Nallal et al. (2021) reported a rapid and efficient solar-assisted green synthesis of AgNPs using *Allium ampeloprasum*, confirmed by a UV–Vis absorption peak at 446 nm. Similarly, Singh et al. (2021) documented peaks at 432 nm and 444 nm for biosynthesized AgNPs at 25 °C and 60 °C, respectively. Further supporting evidence comes from El-Bendary et al. (2021), who observed a peak at 410 nm for AgNPs synthesized by *Aspergillus caespitosus*, and Hashem et al. (2022), who reported an SPR band at 420 nm in AgNPs produced by *Bacillus thuringiensis* MAE 6. Pallavi et al. (2022) also recorded a defined absorption peak at 418 nm for AgNPs synthesized by *Streptomyces hirsutus* strain SNPGA-8. Collectively, these findings confirm the successful biosynthesis of AgNPs and the characteristic optical properties of silver nanostructures (Mekky et al., 2024b). Similar observations were reported by Singaravel and Murugan (2025), who successfully synthesized silver nanoparticles using Musa AAB stem extract. FTIR analysis revealed the presence of hydroxyl, carbonyl, and amine functional groups, suggesting that plant-derived biomolecules such as proteins, carbohydrates, and polyphenols participated in both the reduction of silver ions and stabilization of the resulting nanoparticles. These findings are consistent with the proposed role of phytochemicals in the present study, highlighting their contribution to nanoparticle formation, stability, and biological activity.

### FTIR analysis

**Figure 3:**
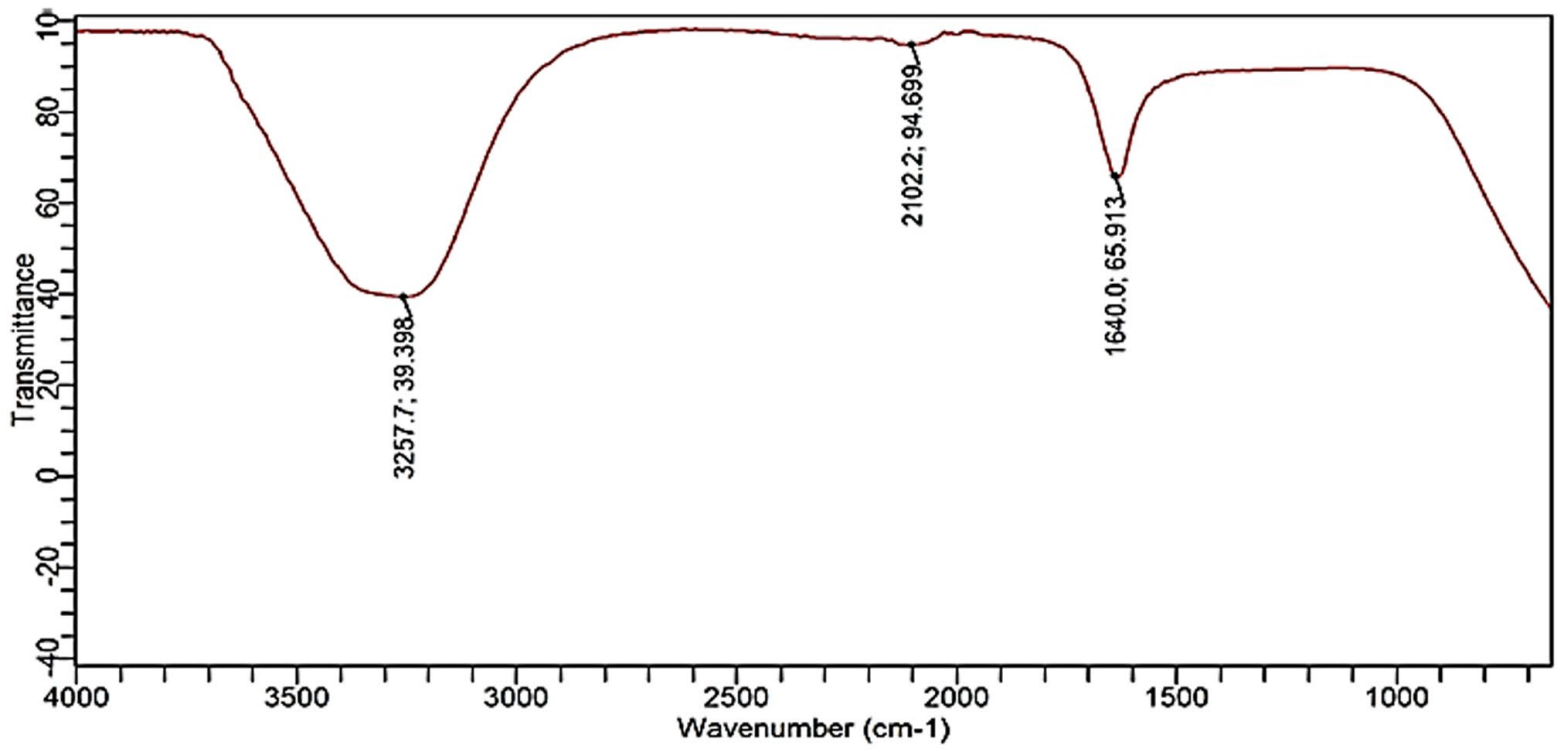
FTIR analysis of biosynthesized AgNPs.

The FTIR spectrum of Ag NPs is presented in Figure 3. A broad and intense absorption band was observed at 3257.7 cm^−**1**^, which is attributed to the O–H stretching vibration of hydroxyl groups, indicating the presence of polyphenols, alcohols, or water molecules associated with the bioactive components of *S. aromaticum* L. extract (Muruganandham et al., 2023). These hydroxyl groups play a crucial role as reducing and stabilizing agents during nanoparticle synthesis (Szczyglewska et al., 2023). Another weak band at 2102.2 cm^−1^ could be attributed to C ≡ C or C ≡ N stretching, typically arising from alkynes or nitriles present in minor quantities in plant phytochemicals or degradation products (Pasieczna-Patkowska et al., 2025). The absorption band at 1640.0 cm^−1^ is assigned to C=O stretching of amide I or conjugated carbonyl groups, often associated with proteins or flavonoids, suggesting their involvement in nanoparticle capping and stabilization (Yadav et al., 2024). These functional groups confirm the role of *S. aromaticum* L. extract’s bioactive constituents in the bioreduction and capping processes, highlighting the green synthesis mechanism. The absence of strong peaks from external contaminants also supports the purity of the synthesized nanomaterial (Vinayagam et al., 2025).

### XRD analysis

**Figure 4:**
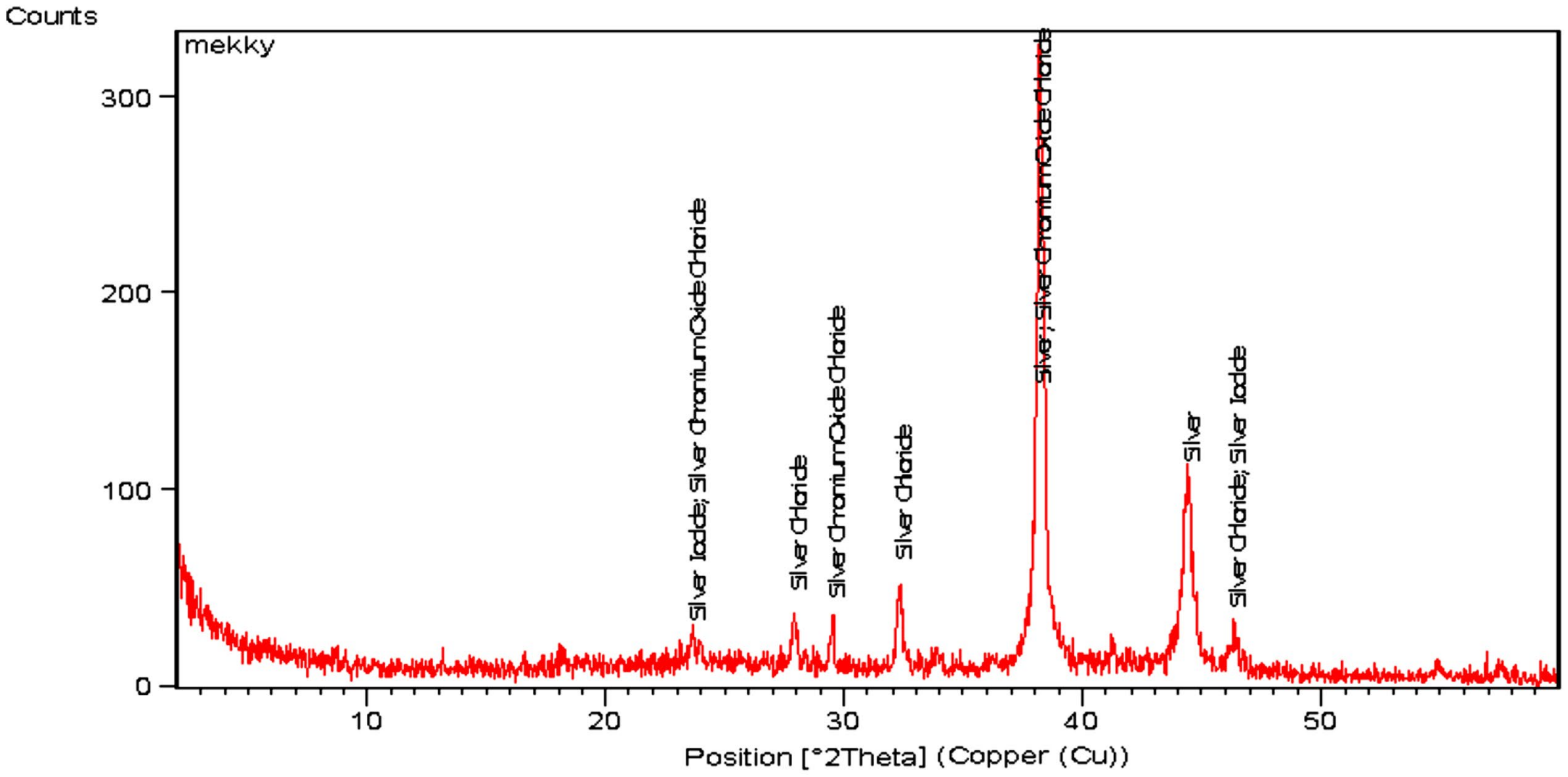
XRD analysis of biosynthesized AgNPs.

The crystalline nature of the biosynthesized AgNPs was examined through XRD analysis. Several distinct diffraction peaks appeared at 2θ angles of approximately 24°, 28°, 29°, 39°, 45.2°, and 47°, which correspond to the characteristic planes (111), (200), (220), and (311) of a face-centered cubic (FCC) silver lattice structure, as illustrated in Figure 4. The mean crystallite size was estimated to be around 11 nm using the Debye–Scherrer equation. These structural features are consistent with those observed by Saied et al. (2022), who reported similar diffraction patterns for silver nanoparticles synthesized using *Cytobacillus firmus*. Other investigations have likewise demonstrated the FCC structure of AgNPs, with particle sizes ranging from 5 to 20 nm (Elshafei et al., 2021; Khanal et al., 2022; Sudarsan et al., 2021). For instance, Pallavi et al. (2022) determined the crystallite size of AgNPs synthesized by *Streptomyces hirsutus* SNPGA-8 to be approximately 12.74 nm. El-Gamal et al. (2018) also confirmed the presence of sharp peaks at typical FCC lattice planes in AgNPs formed by *Streptomyces* sp. Ml-3. Moreover, Ajitha et al. (2019) showed that using *Syzygium aromaticum* (clove) extract led to the formation of AgNPs with prominent XRD peaks at 38.0°, 44.1°, 64.4°, and 77.4°, further confirming their metallic and crystalline nature (Mekky et al., 2024b).

### TEM imaging of ag NPs

Transmission electron microscopy (TEM) analysis confirmed the formation of predominantly spherical and well-dispersed AgNPs with an average size of 9.5 nm. The small particle size and uniform distribution suggest efficient reduction and stabilization by phytochemicals present in the biological extract, which likely acted as both reducing and capping agents, preventing agglomeration and controlling nucleation kinetics. The observed nanoscale dimensions are consistent with the presence of polyphenols and other bioactive compounds that enhance rapid nucleation and limit particle growth (Saied et al., 2022). Comparable studies have reported similar outcomes. For instance, Lan Chi et al. (2022) synthesized BT-AgNPs with sizes between 20.15 and 22.21 nm, while *Juglans regia* (walnut) fruit extract yielded nanoparticles averaging 31.4 nm (Khorrami et al., 2018). LnFb-AgNPs synthesized by Nagaraja et al. (2022) had an average size of 24.5 nm, within a range of 10 to 45 nm. In another case, Hamouda et al. (2019) used *Oscillatoria willlei* NTDM01 to generate larger spherical AgNPs ranging from 100 to 200 nm. Conversely, smaller AgNPs (5–15 nm) were obtained using *Bacillus* sp. KFU36 (Lakhan et al., 2020). Clove bud extract was employed by Lakhan et al. (2020) to produce well-dispersed AgNPs measuring between 10 and 50 nm. Similarly, Hamed et al. (2020) biosynthesized AgNPs from two endosymbiotic actinomycetes isolated from the sponge *Crella cyathophora*, resulting in particles sized between 8.6 and 35.2 nm with both uniform and varied morphologies. Other plant-based approaches, such as using lemon and pomegranate extracts, generated nanoparticles within the 28–32 nm range, as confirmed by various characterization techniques like UV–vis, FTIR, XRD, and TEM (Abdelrazik et al., 2023). Moreover, Alsamman et al. (2023) described biosynthesized spherical AgNPs with smooth surfaces ranging from 5 to 30 nm, predominantly falling within the 10–15 nm interval. In another plant-mediated synthesis, the reported mean particle size reached approximately 52 nm.

### DLS and zeta potential

**Figure 5:**
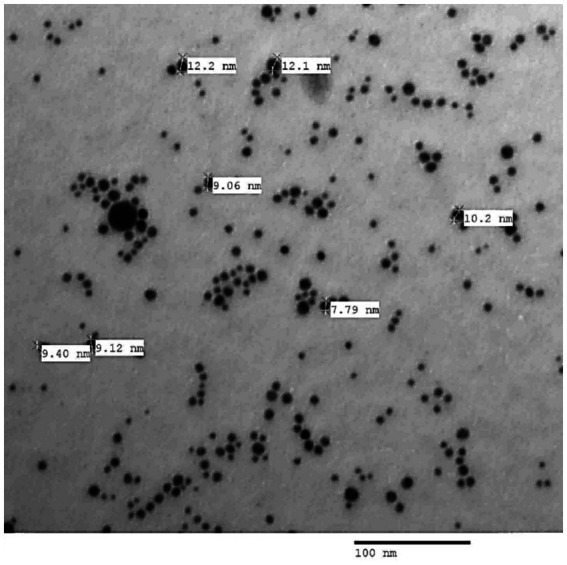
TEM graph of biosynthesized AgNPs.

Dynamic light scattering (DLS) analysis revealed that the synthesized nanoparticles exhibited a narrow and nearly monodisperse size distribution (Figure 5). The average hydrodynamic diameter (Z-average) was 55.0 nm, with a corresponding peak diameter of 55.0 nm for intensity, volume, and number distributions. The polydispersity index (PDI) was 0.223, indicating a relatively homogeneous nanoparticle population with limited size variation. The standard deviation ranged from 12.27 to 12.63 nm, confirming a moderate dispersion around the mean particle size. The single dominant peak observed in the size distribution profile suggests the absence of significant particle aggregation and demonstrates successful synthesis of uniformly distributed nanoparticles. The DLS results demonstrated that the prepared nanoparticles possessed an average hydrodynamic size of approximately 55 nm, which falls within the nanoscale range and is considered suitable for biomedical and antimicrobial applications. The presence of a single, symmetrical peak centered around 55 nm indicates a uniform particle population and good colloidal dispersion. Furthermore, the relatively low PDI value (0.223) suggests a narrow size distribution and acceptable monodispersity, as PDI values below 0.3 are generally indicative of well-dispersed nanoparticle systems.

The hydrodynamic diameter obtained by DLS may be larger than the particle size measured by electron microscopy techniques due to the contribution of the hydration layer and surface-bound biomolecules surrounding the nanoparticles in suspension (Abesekara and Chau, 2022; Kokina et al., 2025). The absence of additional peaks in the distribution profile further confirms minimal aggregation and good colloidal stability. These findings support the effectiveness of the biological synthesis process in producing nanoparticles with controlled size distribution and favorable physicochemical characteristics, which are critical factors influencing cellular uptake, antimicrobial activity, and overall biological performance.

**Figure 6:**
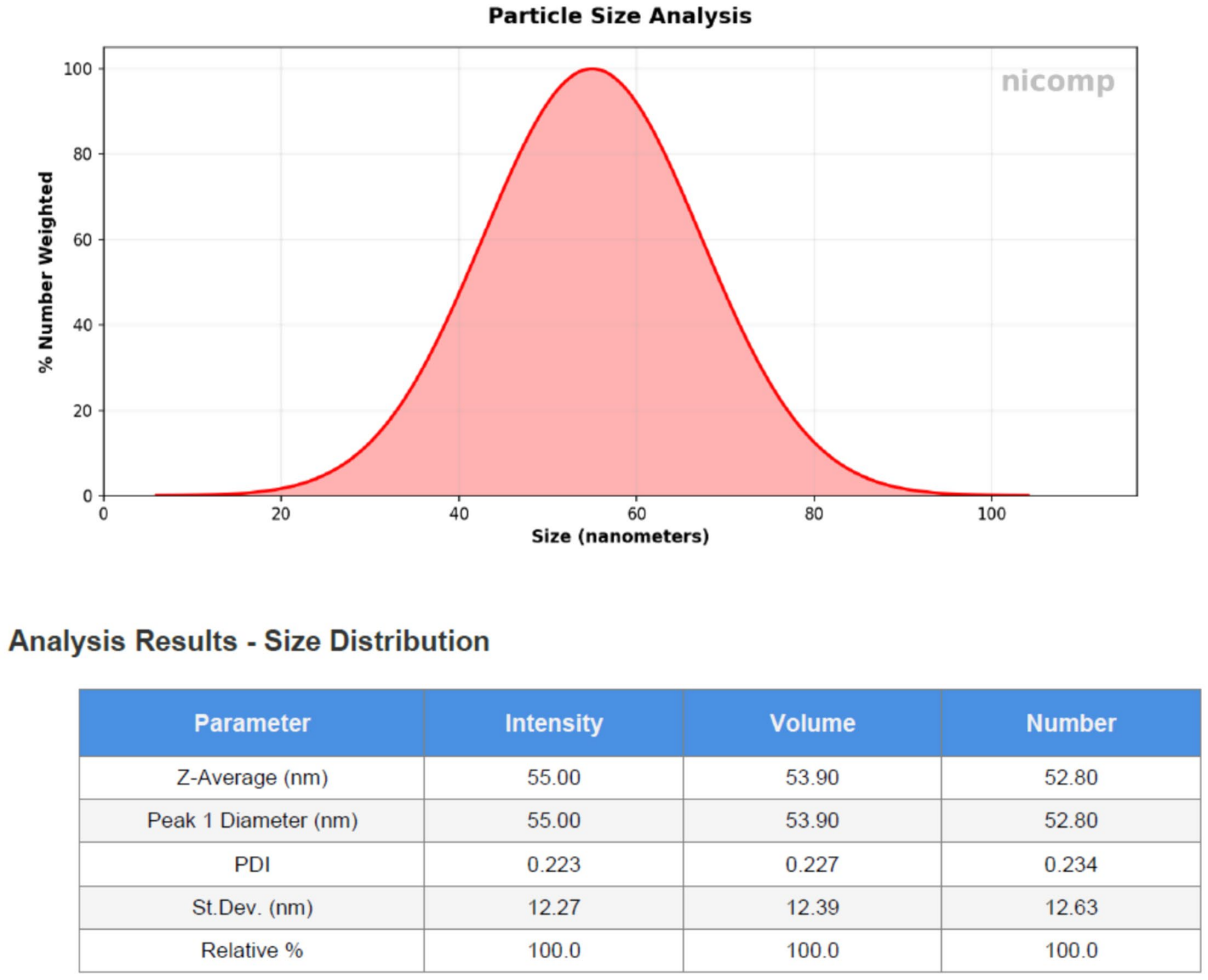
Dynamic light scattering (DLS) analysis showing the particle size distribution of the biosynthesized nanoparticles. The nanoparticles exhibited a mean hydrodynamic diameter (Z-average) of 55.0 nm with a narrow size distribution (PDI = 0.223), indicating good uniformity and dispersion stability.

The zeta potential analysis demonstrated that the biosynthesized nanoparticles possessed a mean surface charge of −30.10 mV (Figure 6). The zeta potential distribution exhibited a single, well-defined peak centered around −30 mV with a narrow deviation of 2.41 mV, indicating a relatively homogeneous surface charge distribution. The electrophoretic mobility was measured at −2.348 μm·cm/V·s, while the conductivity of the suspension was 1.505 mS/cm. Measurements were performed at pH 7.2 ± 0.1 and 23 °C, confirming the stability of the colloidal system under near-physiological conditions. Zeta potential is an important parameter for evaluating the colloidal stability of nanoparticle suspensions because it reflects the magnitude of electrostatic repulsion between particles. In the present study, the synthesized nanoparticles exhibited a zeta potential value of −30.10 mV, indicating good colloidal stability. Generally, nanoparticle dispersions with zeta potential values greater than ±30 mV are considered sufficiently stable due to strong electrostatic repulsive forces that prevent particle aggregation.

The negative surface charge observed in this study may be attributed to the adsorption of negatively charged biomolecules derived from the fungal extract onto the nanoparticle surface (Nxumalo et al., 2024). These biomolecules likely act as both reducing and capping agents during nanoparticle synthesis, contributing to enhanced stability and preventing agglomeration (Javed et al., 2020). The narrow zeta potential distribution and low deviation value further support the uniformity of surface charge among the nanoparticles. Moreover, the observed zeta potential value is consistent with the DLS results, which showed a relatively low PDI and a single size-distribution peak, collectively indicating a well-dispersed and stable nanoparticle suspension. Such stability is advantageous for biomedical applications because it promotes prolonged suspension stability, improved interaction with microbial and cancer cells, and enhanced biological efficacy. Therefore, the obtained zeta potential data confirm that the biosynthesis approach successfully produced nanoparticles with favorable surface characteristics and adequate colloidal stability.

### Antioxidant assays

Reactive oxygen species (ROS) are generated within living cells as a consequence of interactions between biomolecules and molecular oxygen (Di Meo and Venditti, 2020). To mitigate the harmful effects of ROS, antioxidant agents are commonly employed. Although numerous studies have reported potent antioxidant and ROS-scavenging activities of biogenic AgNPs, attributed to surface-bound phytochemicals and capping agents (Shehabeldine et al., 2022; Kumar et al., 2020; Talank et al., 2022), the present study did not experimentally evaluate antioxidant activity or ROS scavenging. Therefore, no experimental conclusions regarding ROS-related effects of the clove-synthesized AgNPs can be drawn.

In this study, the biosynthesized AgNPs exhibited strong, dose-dependent antioxidant activity, as evaluated by the DPPH radical scavenging assay (Kumar et al., 2020). The antioxidant potential of aqueous tomato leaf extract, AgNPs, and standard references was assessed using both DPPH and H₂O₂ radical models. Using ascorbic acid as a positive control, the AgNPs demonstrated DPPH inhibition of 92.5, 85.5, 78.7, 71.7, 64.7, 57.8, 51.1, 44.4, 38.1, and 31.4% at serial concentrations of 1, 0.5, 0.25, 0.12, 0.062, 0.031, 0.015, 0.007, 0.003, and 0.001 mg/mL, respectively, yielding an IC₅₀ of 13.54 μg/mL (Figure 6). These results are consistent with Mekky et al. (2024b), who reported similar inhibition percentages and IC₅₀ values for AgNPs synthesized from tomato leaf extract. However, the antioxidant activity of the AgNPs was lower than that of ascorbic acid (IC₅₀ = 3.45 μg/mL) in both studies. Interestingly, the crude tomato leaf extract displayed higher antioxidant activity than the AgNPs (IC₅₀ = 27 μg/mL), though it remained less effective than ascorbic acid at equivalent concentrations. In comparison, Shehabeldine et al. (2022) reported that chitosan-coated AgNPs (Chi/Ag-NPs) exhibited 92, 90, and 75% antioxidant activity at concentrations of 4,000, 2000, and 1,000 μg/mL, respectively, with an IC₅₀ of 261 μg/mL. This comparatively lower activity was attributed to the presence of bio-reductive phytochemicals on the nanoparticle surface.

**Figure 7:**
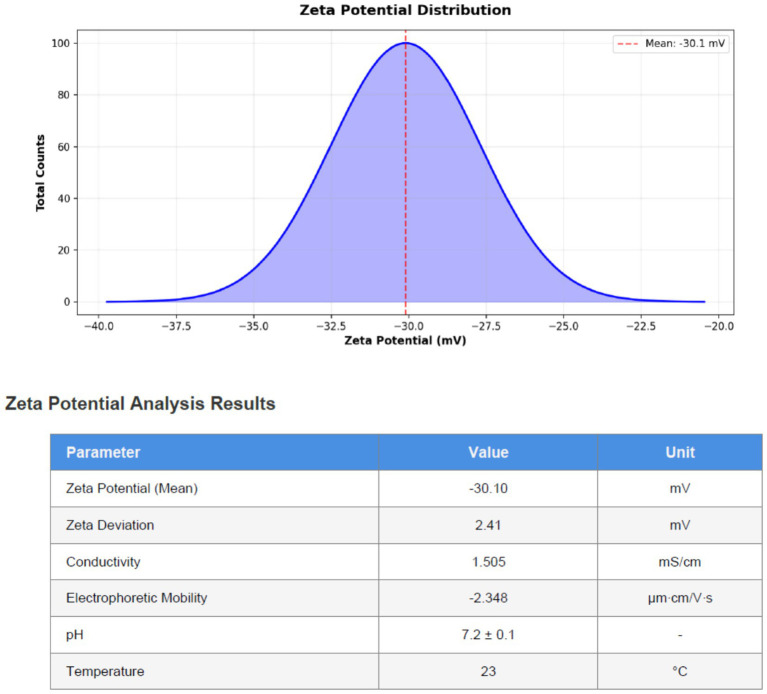
Zeta potential distribution of the biosynthesized nanoparticles. The nanoparticles exhibited a mean zeta potential of −30.10 mV, indicating good colloidal stability and a strong negative surface charge. The narrow distribution profile suggests homogeneous surface properties and minimal particle aggregation.

For instance, Rajivgandhi et al. (2020) reported antioxidant values of 0.765, 0.478, and 0.890 at 250 μg/mL, while Dridi et al. (2022) found that synthetically produced AgNPs exhibited total antioxidant activity comparable to gallic acid, with 9.46 mg EAA/g DM and 5.09% inhibition, alongside a FRAP value of 0.66. Similarly, Rajeshkumar et al. (2018) observed superior antioxidant properties in seaweed-synthesized AgNPs compared to crude extracts. Govindappa et al. (2021) reported enhanced DPPH and H₂O₂ scavenging by Pfp-AgNPs, likely due to their surface chemistry and capping agents, and another study by Talank et al. (2022) who showed biogenic AgNPs achieving 69.73 ± 0.56% DPPH inhibition at 0.5 mg/mL. Collectively, these findings highlight the contribution of nanoparticle structure and surface properties to their antioxidant potential, providing a context for understanding the multifunctional biological activities of AgNPs, although direct ROS or antioxidant assessments were not the primary focus of the present work (see Figure 7).

### Anti- inflammatory activity

**Figure 8:**
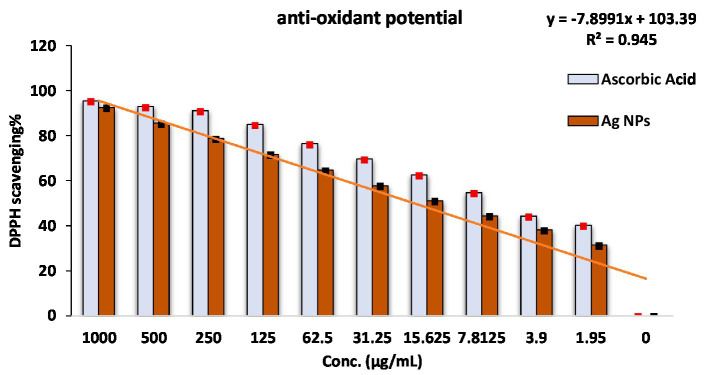
Dose-dependent antioxidant activity of biosynthesized AgNPs compared with ascorbic acid as a standard, measured at various concentrations (μg/mL) using the DPPH radical scavenging assay. Data are presented as mean ± standard deviation of three independent experiments. Statistical differences among groups were analyzed using one-way ANOVA, followed by Tukey’s *post-hoc* test, with significance considered at *p* < 0.05.

The anti-inflammatory potential of the biosynthesized AgNPs was assessed using a hemolysis inhibition assay at concentrations of 1,000, 800, 600, 400, 200, and 100 μg/mL. The corresponding hemolysis inhibition percentages were 95.5, 89.8, 87.2, 85.1, 80.5, and 77.6%, respectively (Figure 8). These values were slightly lower than those observed for the standard anti-inflammatory drug at equivalent concentrations, indicating significant, dose-dependent anti-inflammatory activity. Inflammation is a complex physiological response involving cell membrane destabilization, protein denaturation, and increased vascular permeability. Protein denaturation, induced by heat or oxidative stress, disrupts both secondary and tertiary protein structures (Zaki et al., 2022). Based on the American Society for Testing and Materials (ASTM F 756–00, 2000), chemical substances can be classified according to their hemolytic activity as hemolytic (>5% hemolysis), moderately hemolytic (2–5%), or non-hemolytic (<2%) (Hajji et al., 2019). Comparable studies have reported similar findings. For instance, Chahardoli et al. (2021) demonstrated that quercetin-capped AgNPs exhibited complete (100%) anti-inflammatory activity, attributed to the bioactive coating, which enhances both biocompatibility and nanoparticle stability.

**Figure 9:**
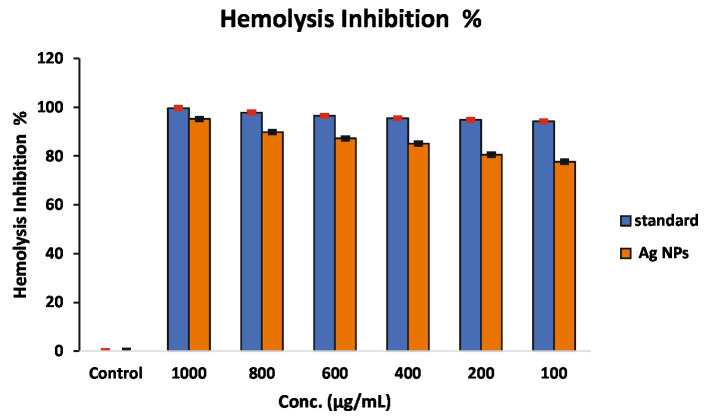
Dose-dependent anti-inflammatory activity of biosynthesized AgNPs at different concentrations compared with the standard drug, showing significant hemolysis inhibition and low hemolytic activity. Data are presented as mean ± SD of three independent experiments. Statistical significance was determined using one-way ANOVA followed by Tukey’s *post-hoc* test (*p* < 0.05).

Further investigations demonstrated that the biosynthesized AgNPs effectively stabilized red blood cell (RBC) membranes, outperforming the standard anti-inflammatory drug indomethacin in membrane protection. The absorbance values reflecting membrane stabilization by AgNPs were 0.027, 0.056, 0.076, 0.092, 0.10, and 0.11 at concentrations of 1,000, 800, 600, 400, 200, and 100 μg/mL, respectively. In contrast, indomethacin exhibited higher absorbance readings of 0.13, 0.22, 0.27, 0.30, 0.39, and 0.44 at the same concentrations (Figure 9), indicating comparatively lower membrane protection. Additionally, hemolysis inhibition assays at lower AgNP concentrations (20, 40, 60, and 80 μg/mL) yielded inhibition rates of 36.6, 55.5, 61.2, and 59.71%, respectively, confirming the dose-dependent membrane-stabilizing and anti-inflammatory effects of the nanoparticles. Sodium diclofenac, used as a reference, achieved higher inhibition percentages of 47.4, 67.1, 72.1, and 69.8% at the corresponding concentrations (Ahmad et al., 2024). Moreover, a study by Anandhi et al. (2019) reported that Ag NPs could stabilize membranes with efficiency ranging between 15 and 72% by reducing hypotonicity-induced erythrocyte lysis. Collectively, these findings demonstrate that green-synthesized Ag NPs possess significant anti-inflammatory and membrane-stabilizing activities, supporting their potential use in therapeutic applications. The anti-inflammatory potential of the green-synthesized AgNPs was preliminarily assessed using hemolysis inhibition and RBC membrane stabilization assays at various concentrations. While these tests indicate that the nanoparticles may stabilize cell membranes and inhibit hemolysis to some extent, it should be emphasized that such ex vivo assays provide only preliminary indications of anti-inflammatory activity and do not directly reflect *in vivo* physiological effects. Comparisons with standard anti-inflammatory drugs suggest lower or comparable performance in these simple assays, but further in vivo studies are necessary to evaluate therapeutic relevance (Hajji et al., 2019; Chahardoli et al., 2021; Ahmad et al., 2024; Anandhi et al., 2019; Kumar et al., 2019).

### AgNPs activity to MDR bacterial strains

The biosynthesized AgNPs were evaluated for their antibacterial efficacy against four MDR bacterial strains using the agar well diffusion assay, including two Gram-positive strains (*S. aureus* ATCC 27217 and *S. haemolyticus* ATCC 29970) and two Gram-negative strains (*E. coli* BAA-197 and *K. pneumoniae* BAA-1705). Gentamycin served as the positive control, while sterile distilled water was used as a negative control. AgNPs demonstrated significant antibacterial activity, with MIC and MBC values of 16 μg/mL and 32 μg/mL, respectively, against the Gram-positive isolates, whereas Gram-negative strains required higher concentrations (both MIC and MBC at 32 μg/mL) to achieve comparable inhibition. This difference is likely attributable to the outer lipopolysaccharide LPS layer in Gram-negative bacteria, which acts as a permeability barrier, consistent with previous reports (Vinicius de Oliveira Brisola Maciel et al., 2020).

**Figure 10:**
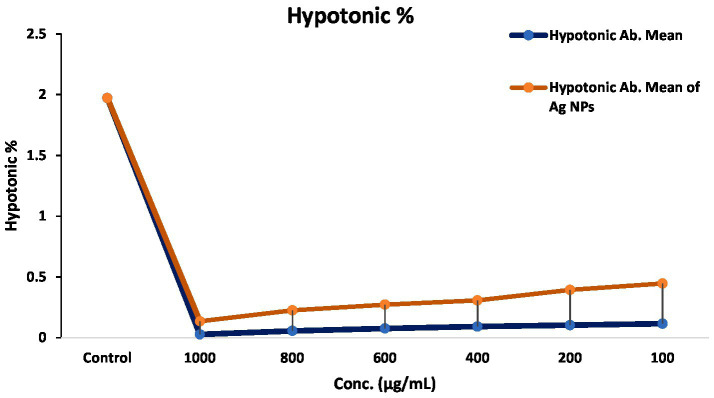
Effect of biosynthesized AgNPs on red blood cell (RBC) membrane stabilization under hypotonic osmotic stress compared with the standard drug. Data are presented as mean ± SD of three independent experiments, with statistical analysis performed using one-way ANOVA followed by Tukey’s *post-hoc* test (*p* < 0.05).

*K. pneumoniae* BAA-1705 exhibited the highest susceptibility with a 36 mm inhibition zone, followed by *S. aureus* ATCC 27217 with 34 mm, while *S. haemolyticus* ATCC 29970 and *E. coli* BAA-197 showed zones of 29 mm, corresponding to 193–200% relative to the gentamycin standard (see Table 3 and Figure 8). These results align with prior studies reporting MIC values of 7.8 μg/mL for *Salmonella Typhimurium* and 3.9 μg/mL for *E. coli* and *K. pneumoniae* (Loo et al., 2018), and MICs of 3 mg/mL and 2 mg/mL for *S. aureus* and *Enterococcus faecalis*, respectively (Khaydarov et al., 2011; Yousef et al., 2022). According to Alsamman et al. (2023), AgO nanoparticles showed the highest antimicrobial activity among several tested nanomaterials against MDR isolates, with TiO₂ nanoparticles ranked second. Additionally, clove-derived AgNPs exhibited bacteriostatic effects at 40 μg/mL, while higher concentrations (60–100 μg/mL) of clove- and eugenol-based AgNPs demonstrated bactericidal properties. These outcomes underscore their efficacy, particularly against Gram-positive pathogens such as *S. aureus*. Although the exact antibacterial mechanisms of AgNPs were not directly assessed in the present study, literature suggests that AgNPs can interact with bacterial cell envelopes, potentially leading to membrane disruption and increased permeability. Silver ions may also interact with cellular components, including DNA and proteins, which could contribute to impaired replication and protein synthesis (Alsamman et al., 2023). It should be emphasized that these mechanistic interpretations are based solely on previously published studies and were not experimentally verified in this work (see Figure 10).

**Table 3:** Antibacterial activity of clove-mediated AgNPs against MDR bacterial strains for 24 h.

No.	Tested strains	Inhibition zone (mm) by 100 μL		
		Control		AgNPs
		Gentamycin	D. H_2_O	
1	*S. aureus* ATCC 27217	17 ± 0.5	0.0 ± 0.0	34 ± 1.2
2	*S. haemolyticus* ATCC 29970	15 ± 0.4	0.0 ± 0.0	29 ± 1.0
3	*E.coli* BAA-197	16 ± 0.6	0.0 ± 0.0	29 ± 0.9
4	*K. pneumoniae* BAA-1705	18 ± 0.5	0.0 ± 0.0	36 ± 1.1

Gentamycin (positive control) and distilled water (negative control). Values are expressed as the mean of three independent replicates ± standard error (SE).

### Inhibition of virulence factors expression by AgNPs

**Figure 11:**
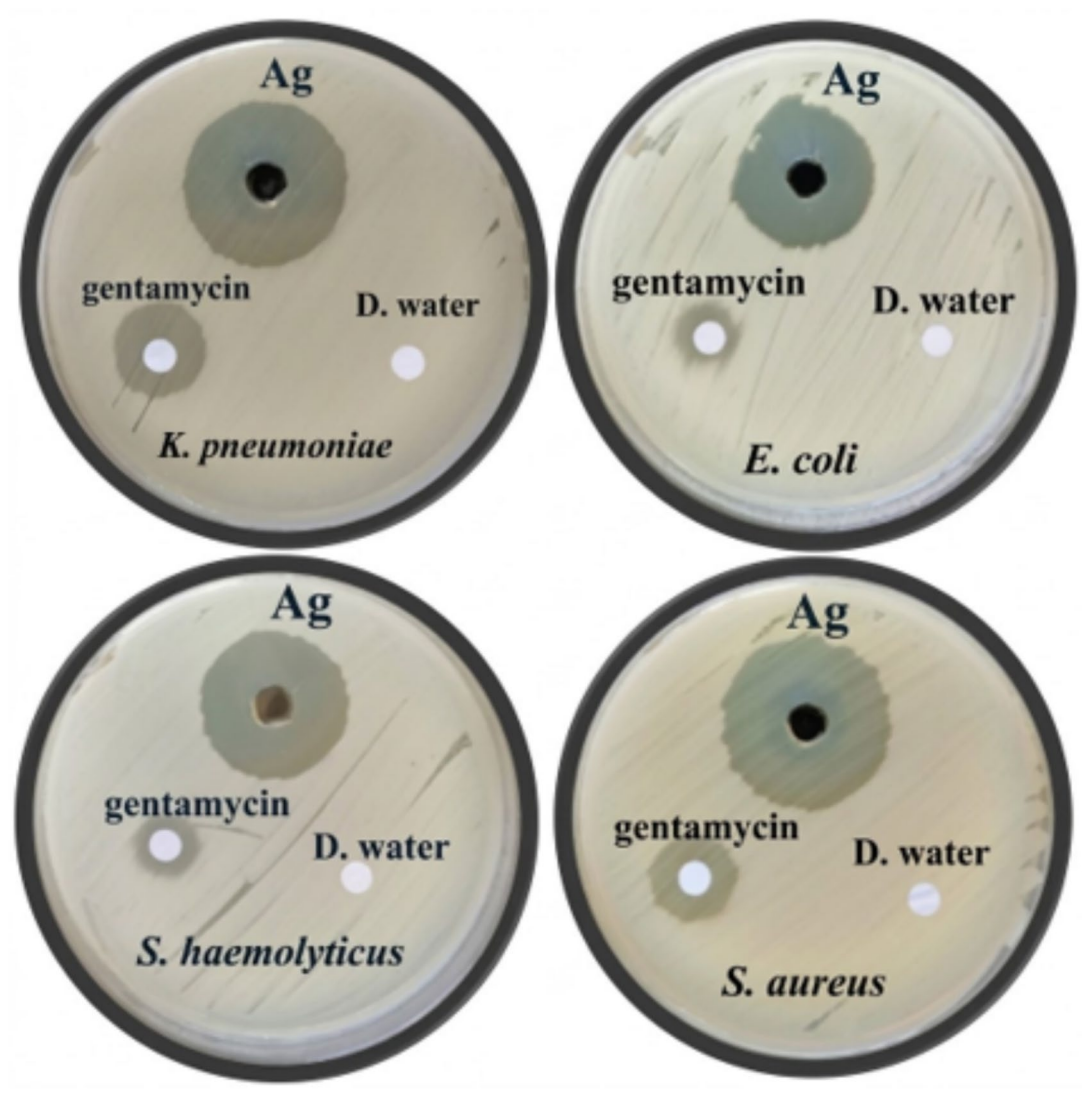
Antibacterial activity of Clove-AgNPs against MDR bacterial strains for 24 h at 37 °C through agar well diffusion method on Muller Hinton agar plates.

The hypervirulent phenotype of *K. pneumoniae* is associated with several genes, including *rmpA* and the mucoviscosity-associated gene A (*magA*) (Khattab and Hager, 2022), while *Cna* functions as an adhesin mediating colonization onto host tissue surfaces (Madani et al., 2017), and the *fnbA* gene in *S. aureus* is involved in biofilm formation (Abdulqader and Abood, 2024). The *luxS* gene mediates the quorum sensing (QS) mechanism in *E. coli* (Han et al., 2013). These genes were selected as targets to investigate the potential effects of clove-derived AgNPs on pathogenic MDR bacterial strains. Total RNA was extracted from both control (untreated) and AgNP-treated bacterial samples and reverse transcribed into cDNA. qRT-PCR was used to assess the relative expression of the selected virulence genes, and densitometric analysis of band intensity was performed to estimate the degree of downregulation. Fold changes in gene expression are presented in Figure 11. Among the tested genes, *luxS* in *E. coli* showed the highest downregulation (70%), followed by *fnbA*-a and *cna* in *S. aureus* (74%), and *fnbA-h* in *S. haemolyticus* (77%). The least responsive gene was *rmpA* in *K. pneumoniae*, showing 81% expression relative to control. The AgNP concentrations used were 10 μg/mL for Gram-positive strains and 12.5 μg/mL for Gram-negative strains. These findings are in line with previous reports; for example, Mekky et al. (2024b) demonstrated that AgNPs reduced the expression of *Cna* and *FnbA*, with *fnbA* downregulated by 6% and *Cna* by 12.5%. It should be emphasized that the observed downregulation in the present study provides preliminary mechanistic insight and was not complemented by phenotypic assays such as biofilm quantification or adhesion studies.

### Antiviral activity determination

**Figure 12:**
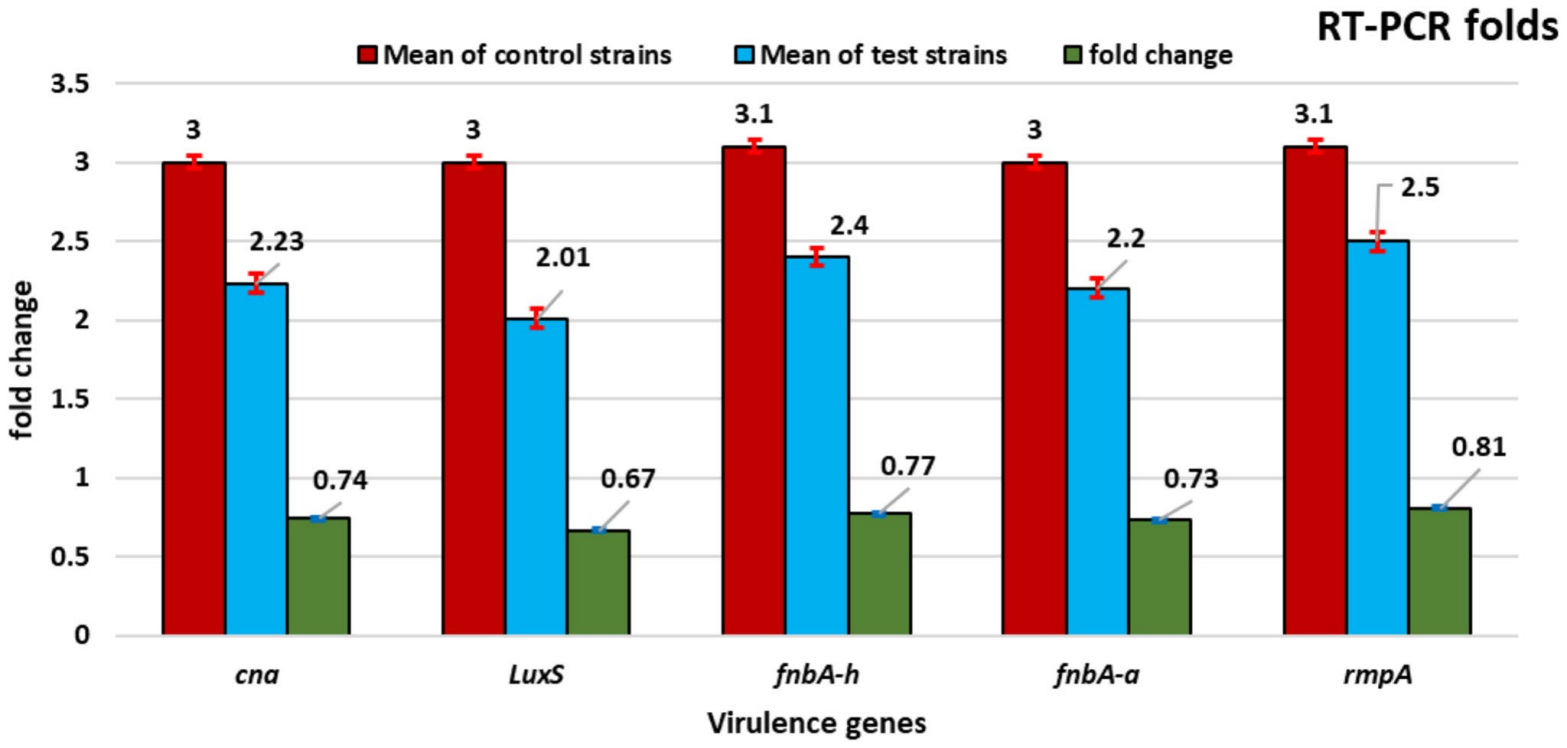
Fold change in gene expression of the tested bacterial strains measured by qRT-PCR, comparing cells treated with AgNPs (blue columns) to untreated controls (red columns). Data are presented as mean ± SD of three independent experiments, with statistical significance determined by one-way ANOVA followed by Tukey’s *post-hoc* test (*p* < 0.05).

**Figure 13:**
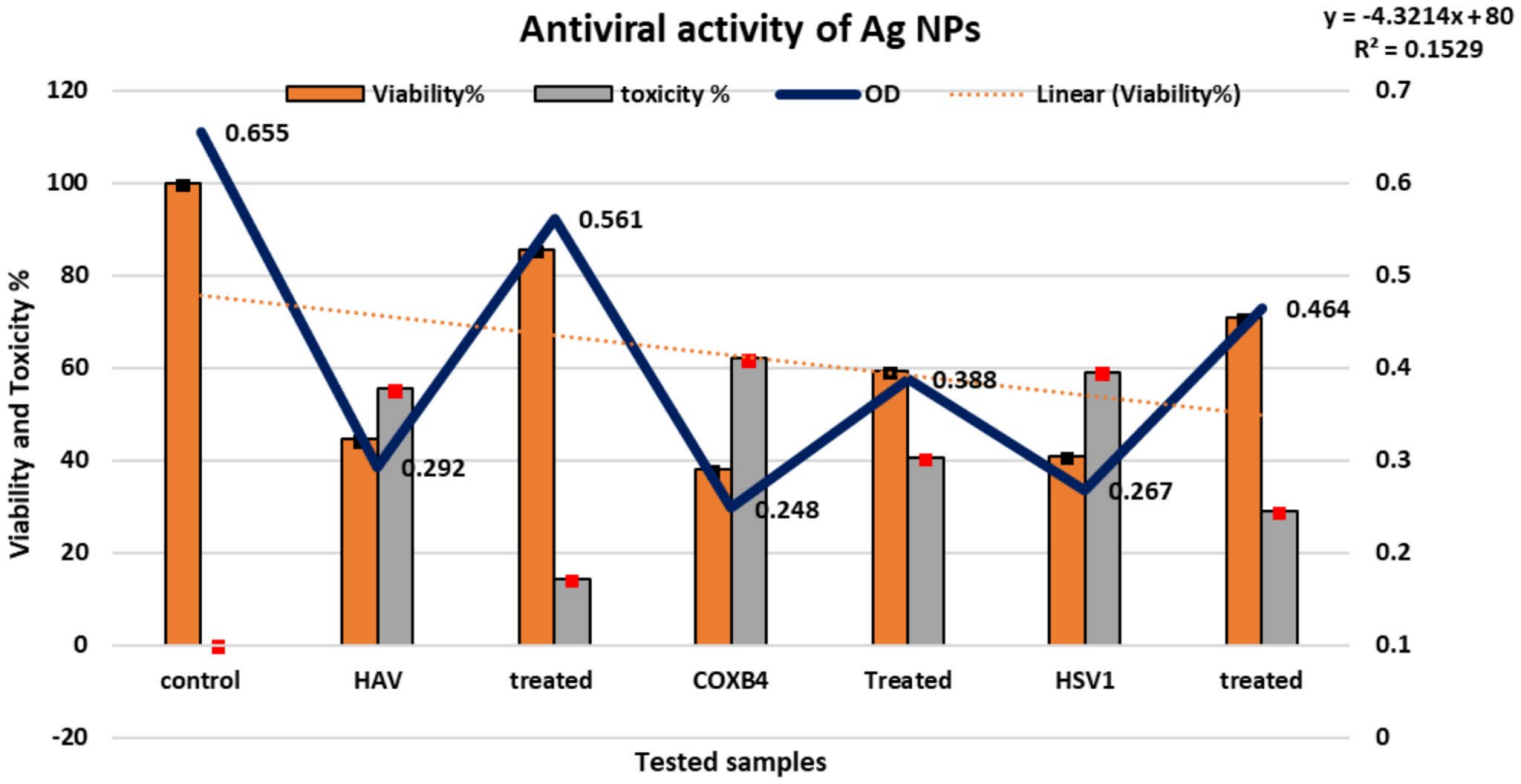
Comparison of cell viability and cytotoxicity among healthy control, virus-infected, and AgNP-treated samples. Data are expressed as mean ± SD of three independent experiments, with statistical significance analyzed using one-way ANOVA followed by Tukey’s *post-hoc* test (*p* < 0.05).

Globally, viral infections remain a significant public health concern. In this study, green biosynthesized AgNPs demonstrated effective antiviral activity *in vitro* at a concentration of 250 μg/mL against three human viruses: HAV, COXB4, and HSV1, as determined using the MTT assay on Vero cell lines. Treatment with AgNPs increased cell viability in infected cultures: from 44.5 to 85.6% for HAV, from 37.9 to 59.3% for COXB4, and from 40.8 to 70.9% for HSV1. Toxicity induced by viral infection was also reduced in all treated samples, reaching 14.3% against 55.4% for HAV, 40.6% against 62% for COXB4, and 29% against 59.1% for HSV1 (Figure 12). Previous studies have also reported antiviral potential of plant-mediated AgNPs. For example, AgNPs synthesized using lemon peel and juice showed dose-dependent inhibition of HSV-1 and SARS-CoV-2 replication (Dell'Annunziata et al., 2024). In our study, among the tested viruses, AgNPs were most effective against HAV (74.1%), followed by HSV1 (50.9%) and COXB4 (34.5%) (Figure 13). For comparison, ZnO nanoparticles demonstrated antiviral activity against HSV1 and COXB4 of 75.4 and 65.8%, respectively, at 62.5 μg/mL, with IC50 = 526 μg/mL (Almuhayawi et al., 2024).

It should be emphasized that these observations are limited to *in vitro* assays. No *in vivo* or immune-mediated studies were performed, and potential immune-related or inflammatory responses associated with AgNP treatment were not evaluated. Therefore, any broader extrapolation to clinical or emerging viral threats should be considered preliminary and cautious, in line with Zhang et al. (2023). Future studies are planned to evaluate the antiviral mechanism in greater detail, including viral entry/replication assays, nanoparticle-virus interaction studies, and calculation of selectivity indices to distinguish specific antiviral effects from general cytoprotection.

### Anticancer activity of ag NPs

**Figure 14:**
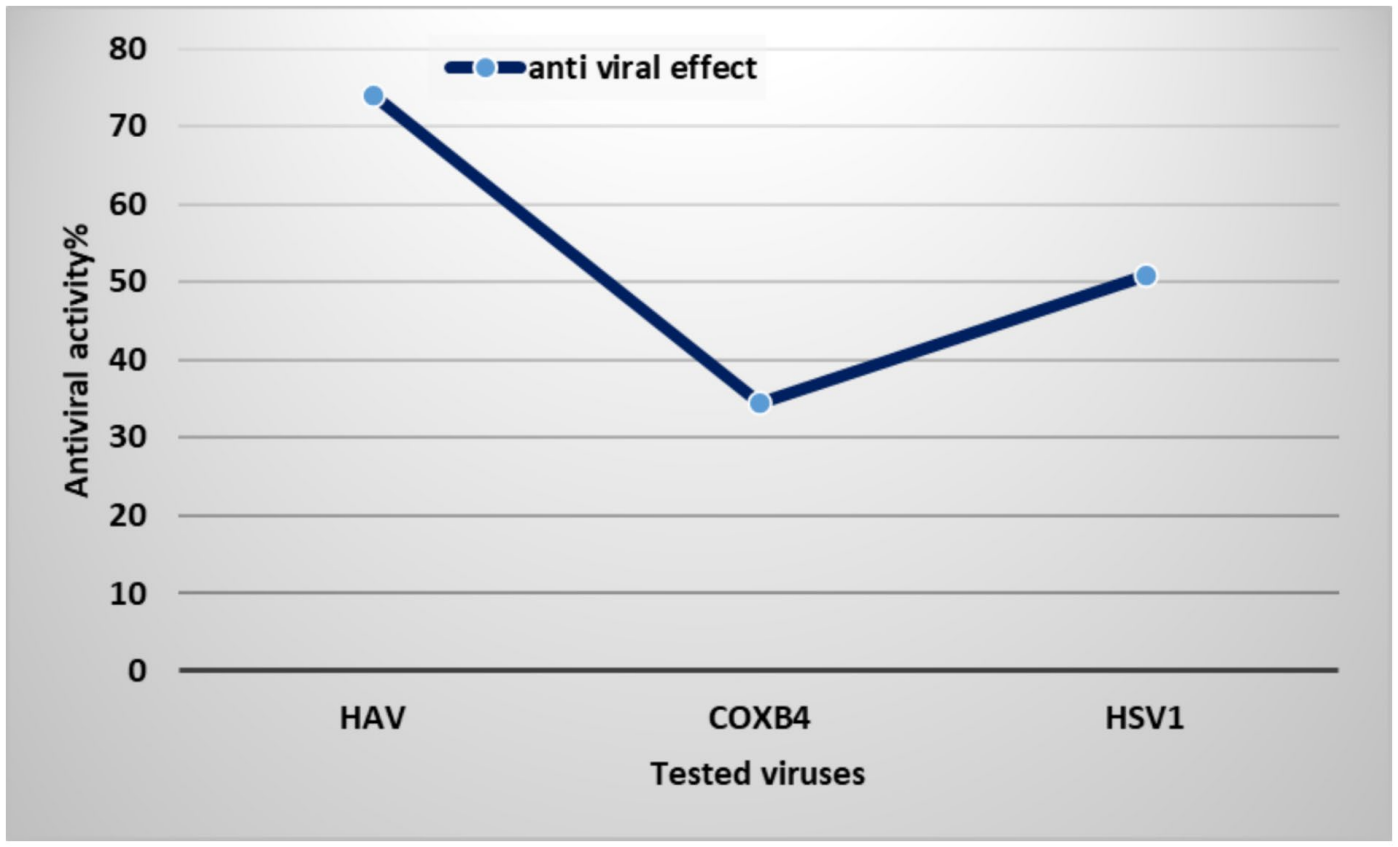
Antiviral activity % of AgNPs against three human viruses.

**Figure 15:**
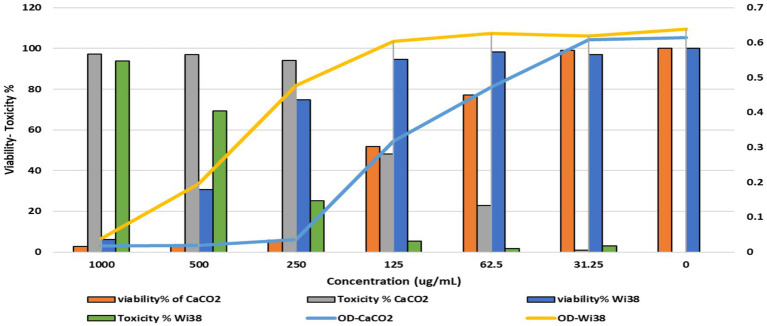
Cell viability and cytotoxicity of CaCO₂ and Wi-38 cell lines following treatment with varying concentrations of biosynthesized AgNPs. Data are expressed as mean ± SD of three independent experiments. Statistical significance was evaluated using one-way ANOVA followed by Tukey’s *post-hoc* test (*p* < 0.05).

The anticancer efficacy of the biosynthesized AgNPs was evaluated against CaCO_2_ and WI-38 cell lines using a concentration gradient (31.25 to 1,000 μg/mL). In CaCO_2_ cells, AgNPs induced 48.2% cytotoxicity at 125 μg/mL, with an IC50 value of 137.35 ± 4.55 μg/mL. A dose-dependent response was evident, with cytotoxicity increasing to 94.1% at 250 μg/mL, 96.9% at 500 μg/mL, and reaching 97.2% at 1000 μg/mL (Figure 14). Conversely, WI-38 cells demonstrated greater resilience; only 5.2% cytotoxicity was observed at 125 μg/mL, and the IC50 was significantly higher at 386.88 ± 7.71 μg/mL. Toxicity gradually rose to 69.2% at 500 μg/mL and peaked at 93.8% at 1000 μg/mL (Figure 15), indicating comparatively lower sensitivity than cancerous CaCO_2_ cells. These results are consistent with previous studies. For example, AgNPs synthesized using pomegranate extract were non-toxic to normal WI-38 fibroblasts but exhibited substantial growth inhibition in HepG2, MDA-MB-231, and HeLa cancer cells (Hassan et al., 2024). Similarly, AgNPs have been shown to exert cytotoxicity against MCF-7 breast cancer cells and RAW 264.7 macrophages, with macrophages displaying higher susceptibility, an effect attributed to increased intracellular ROS levels (Wypij et al., 2021). AgNPs synthesized from *Azadirachta indica* fruit extract also significantly reduced viability in A549 lung cancer cells (Alharbi and Alsubhi, 2022), and colloidal AgNPs (CN–Ag) decreased CaCO_2_ viability by 50% at 5 μg/mL after 48 h (Zein et al., 2020). Computational studies further suggest that clove-derived phytochemicals have strong affinities for apoptosis-related proteins in breast cancer, supporting experimental findings with clove-mediated AgNPs (CL-AgNPs) against MCF-7 cells (IC50 = 58.64 μg/mL) (Vishal et al., 2023). It should be emphasized that these observations are limited to *in vitro* MTT assays on two cell lines. No additional mechanistic assays, such as apoptosis, cell-cycle, or oxidative stress markers, were conducted. Therefore, the findings should be interpreted cautiously, and potential off-target toxicity or inflammatory responses, as reported in related studies by Jia et al. (2023), were not evaluated (Abdel Ghany Elrahmany et al., 2023; Andleeb, 2020; Gheytanchi et al., 2021; Sabit et al., 2021; Lo et al., 2014).

### qPCR of oncogenes and apoptotic markers in CaCO2 cell line

The impact of AgNPs on the expression levels of various oncogenic and apoptosis-regulating genes was found to be significant. The expression level of c-MYC transcription factor that is responsible for proliferation was decreased significantly to 0.49-fold (*p* < 0.001) (Table 4). The expression of the K-RAS oncogene, which regulates the KRAS/ERK cascade, associated with the colorectal cancer progression was decreased significantly to 0.67-fold (*p* < 0.001) (Table 4). The anti-apoptotic oncomarker BCL2 exhibited statistically significant downregulation to an extent of around 0.78 relative expression level when compared to the control group (*p* < 0.01). However, there was a statistically significant upregulation of the pro-apoptotic BAX marker, with 1.74 relative expression level compared to the control (*p* < 0.01).

**Table 4:** Oncogenes and apoptotic markers expression through qPCR.

Gene	Fold change	ΔCt control	ΔCt treated	Regulation
MYC (c-MYC)	0.49	0.033	1.067	Down (51%)
KRAS	0.67	−0.033	−0.323	Down (33%)
BCL2	0.78	0.467	0.867	Down (22%)
BAX	1.74	1.033	0.2	Up (74%)

To gain further insight into the potential anticancer mechanism, the expression of selected oncogenic and apoptosis-related genes was evaluated by qPCR in CaCO2 cells following AgNP treatment. The results demonstrated significant downregulation of c-MYC (0.49-fold), KRAS (0.67-fold), and BCL2 (0.78-fold), accompanied by a significant upregulation of the pro-apoptotic marker BAX (1.74-fold). These findings suggest that the observed reduction in CaCO2 cell viability may be associated with suppression of proliferation-related pathways and activation of apoptotic signaling. Nevertheless, the present study did not include direct apoptosis assays, ROS measurements, mitochondrial membrane potential analysis, or cell-cycle investigations. Therefore, while the gene expression data provide preliminary mechanistic support for the anticancer activity of the synthesized AgNPs, further studies are required to confirm the underlying molecular pathways.

## Conclusion

Silver nanoparticles (AgNPs) synthesized using clove extract, characterized by a uniform spherical shape and an average particle size of approximately 9.5 nm, demonstrated notable multifunctional biological properties *in vitro*. These nanoparticles exhibited strong antioxidant potential, with a low IC₅₀ value of 13.54 μg/mL, indicating efficient free radical scavenging. They also showed membrane-protective effects by inhibiting hemolysis and stabilizing HRBC membranes, suggesting promising biocompatibility. At a concentration of 250 μg/mL, clove-mediated AgNPs exhibited antiviral activity against HAV, COXB4, and HSV-1, while in anticancer assessments, they reduced the viability of CaCO_2_ colorectal cancer cells (IC50 = 137.35 μg/mL) and showed comparatively lower cytotoxicity toward normal Wi-38 fibroblast cells (IC50 = 386.88 μg/mL), suggesting a preliminary selective effect. The downregulation of c-MYC, K-RAS, and BCL2 genes and upregulation of the BAX gene due to the AgNP treatment imply that there is inhibition of cancer-causing signals and initiation of mitochondrial-mediated apoptosis in CCA cells. Consequently, the use of AgNPs can serve as an effective nanotherapeutic strategy against colorectal cancer cells. Antimicrobial evaluation revealed inhibition of several extensively drug-resistant (XDR) bacterial pathogens, including *S. aureus* (ATCC 27217), *S. haemolyticus* (ATCC 29970), *E. coli* (BAA-197), and *K. pneumoniae* (BAA-1705). The AgNPs also interfered with bacterial virulence by downregulating key pathogenic genes (*luxS, fnbA-a, cna, fnbA-h,* and *rmpA*), indicating potential dual antimicrobial and anti-virulence mechanisms. It should be emphasized that these findings are based solely on *in vitro* assays. No *in vivo* validation, pharmacokinetic evaluation, or long-term safety studies were conducted. Therefore, while the results provide valuable preliminary insights into the biological activities of clove-derived AgNPs, further research is required to evaluate their therapeutic potential, safety, and translational applicability in clinical contexts.

## Data Availability

The datasets presented in this study can be found in online repositories. The names of the repository/repositories and accession number(s) can be found in the article/Supplementary material.
