# Supplementary material for: Clove-mediated green synthesis of silver nanoparticles and in vitro investigation of their antimicrobial, antioxidant, and anticancer potential
Source: Front Microbiol. 2026 Jul 28;17:1860045. doi: 10.3389/fmicb.2026.1860045 (PMC13457700; doi:10.3389/fmicb.2026.1860045)

# *My GC-MS Report*

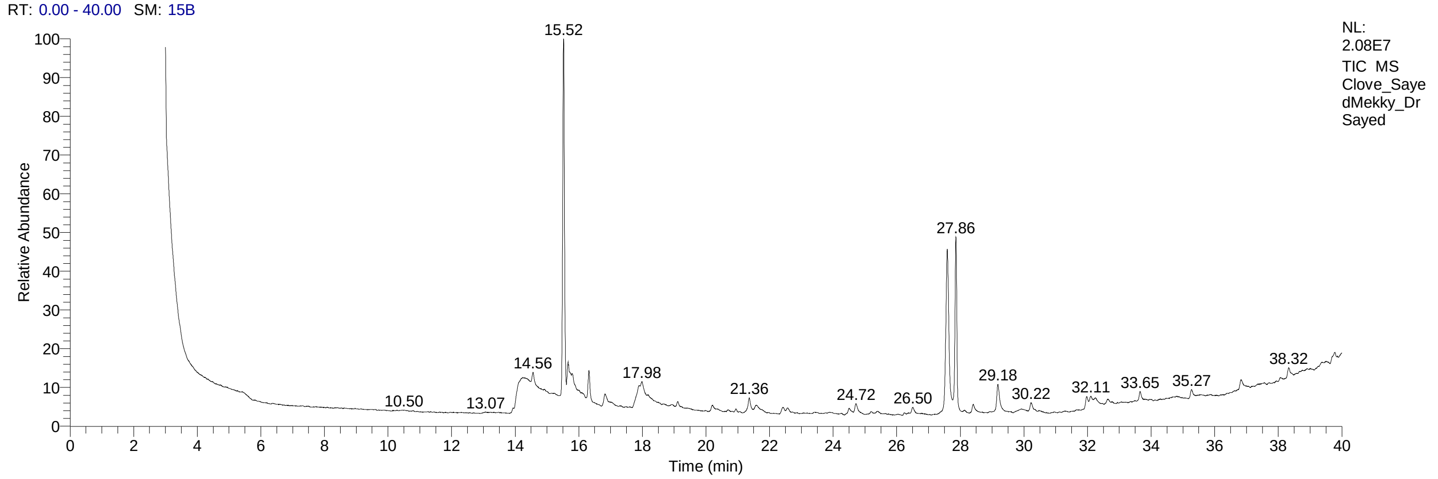


| RT | Area % | Peak Area | Peak Height |
|---|---|---|---|
| 14.11 | 2.74 | 6362467.02 | 987440.54 |
| 14.18 | 5.23 | 12140197.88 | 982521.88 |
| 14.56 | 1.15 | 2679617.29 | 677266.85 |
| 15.52 | 31.25 | 72592910.96 | 22082101.06 |
| 15.66 | 2.30 | 5352970.15 | 1495120.84 |
| 15.73 | 1.01 | 2357496.71 | 853731.45 |
| 15.80 | 1.86 | 4315373.78 | 860710.77 |
| 16.32 | 2.73 | 6339261.29 | 1788552.67 |
| 16.82 | 1.28 | 2982668.07 | 557752.51 |
| 17.89 | 1.62 | 3773765.41 | 636300.10 |
| 17.98 | 1.66 | 3861153.28 | 756105.79 |
| 21.36 | 1.48 | 3441506.68 | 730357.99 |
| 24.72 | 1.10 | 2561707.36 | 498073.43 |
| 27.59 | 18.61 | 43241550.92 | 8153992.95 |
| 27.86 | 14.63 | 33987890.42 | 10593378.04 |
| 29.18 | 3.02 | 7006263.01 | 1405908.98 |
| 30.22 | 1.09 | 2522757.50 | 482526.38 |
| 31.97 | 1.35 | 3124705.74 | 642804.62 |
| 32.10 | 1.57 | 3658283.29 | 560962.89 |
| 33.65 | 0.99 | 2307579.96 | 536547.70 |
| 36.82 | 1.09 | 2530895.69 | 471810.98 |
| 38.33 | 1.21 | 2804378.57 | 528655.43 |
| 39.78 | 1.02 | 2364096.91 | 482532.92 |

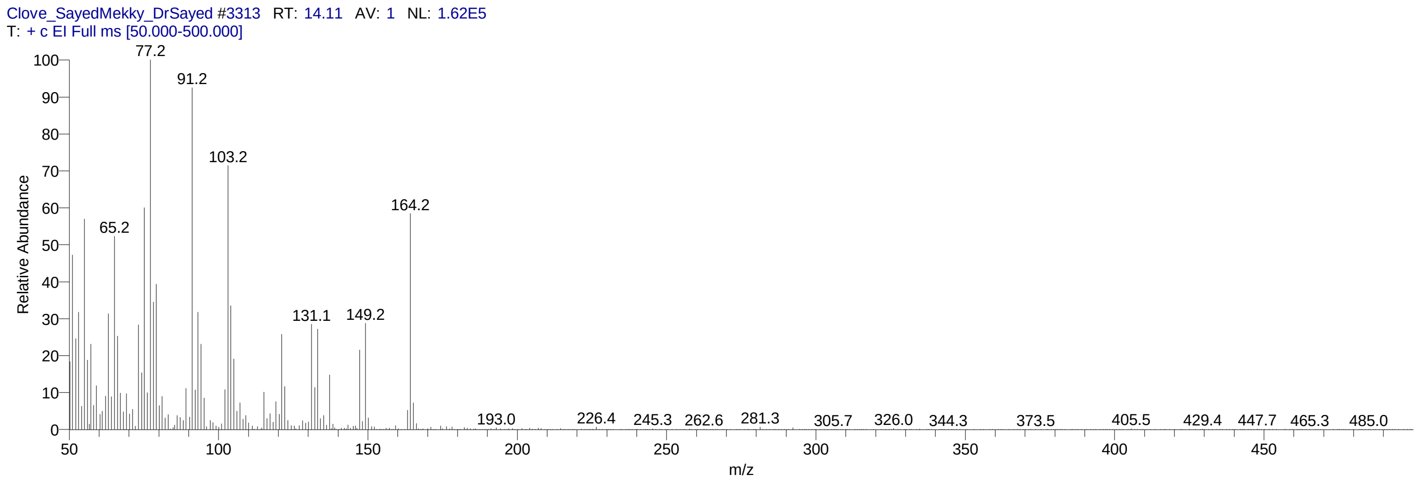

# My GC-MS Report

| RT | Compound Name | Area % | MF | Molecular Formula | Molecular Weight | Cas # | Library |
|---|---|---|---|---|---|---|---|
| 14.11 | PHENOL, 2-METHOXY-4-(2-PROPENYL)- | 2.74 | 817 | C10H12O2 | 164 | 97-53-0 | WileyRegistry8e |
| 14.11 | PHENOL, 2-METHOXY-4-(2-PROPENYL)- | 2.74 | 818 | C10H12O2 | 164 | 97-53-0 | WileyRegistry8e |
| 14.11 | Phenol, 2-methoxy-3-(2-propenyl)- | 2.74 | 799 | C10H12O2 | 164 | 1941-12-4 | mainlib |
| 14.11 | PHENOL, 2-METHOXY-3-(2-PROPENYL)- | 2.74 | 799 | C10H12O2 | 164 | 1941-12-4 | WileyRegistry8e |
| 14.11 | Eugenol | 2.74 | 803 | C10H12O2 | 164 | 97-53-0 | replib |

Compound Structure | Hit Spectrum

PHENOL, 2-METHOXY-4-(2-PROPENYL)-
Formula C10H12O2, MW 164, CAS# 97-53-0, Entry# 46893
4-ALLYL-2-METHOXY-PHENOL

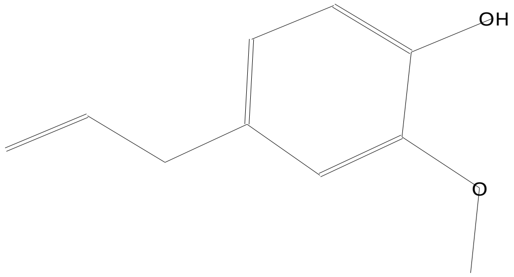


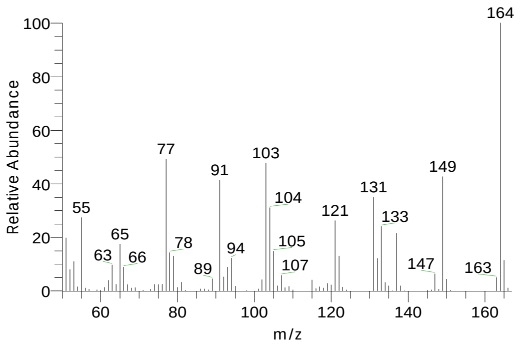


PHENOL, 2-METHOXY-4-(2-PROPENYL)-
Formula C10H12O2, MW 164, CAS# 97-53-0, Entry# 46886
4-ALLYL-2-METHOXY-PHENOL

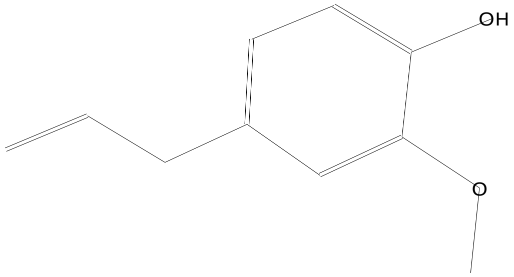


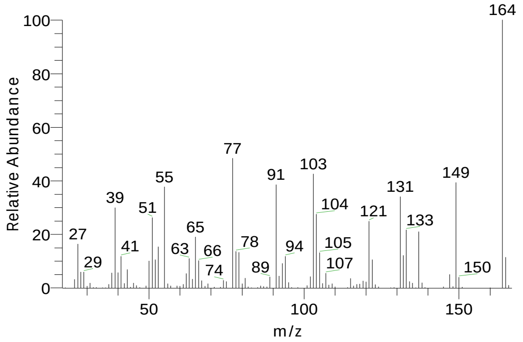


Phenol, 2-methoxy-3-(2-propenyl)-
Formula C10H12O2, MW 164, CAS# 1941-12-4, Entry# 153401
3-Allyl-2-methoxyphenol #

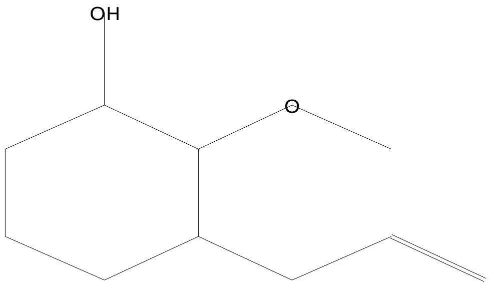


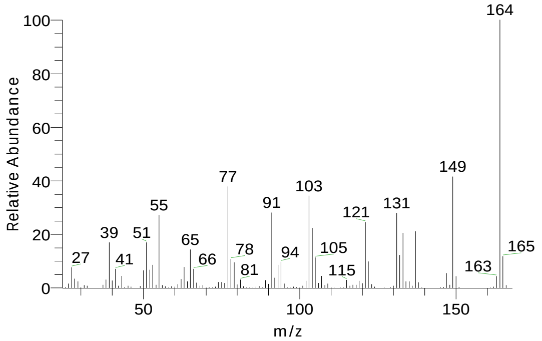


PHENOL, 2-METHOXY-3-(2-PROPENYL)-
Formula C10H12O2, MW 164, CAS# 1941-12-4, Entry# 390451
3-ALLYL-2-METHOXYPHENOL #

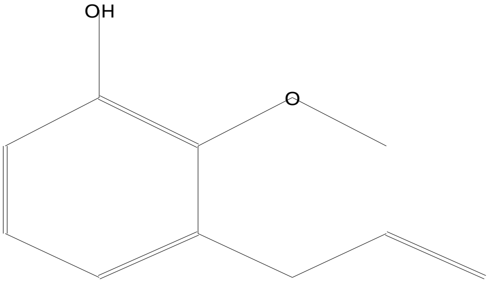


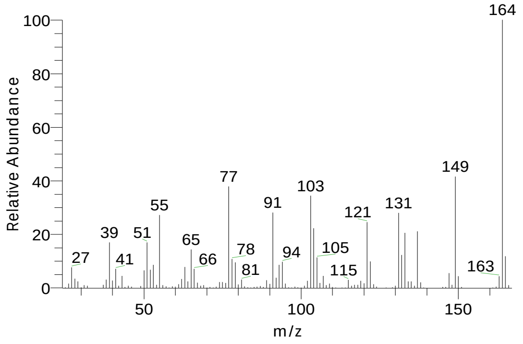

# *My GC-MS Report*

Compound Structure | Hit Spectrum

Eugenol
Formula C10H12O2, MW 164, CAS# 97-53-0, Entry# 25332
Phenol, 2-methoxy-4-(2-propenyl)-

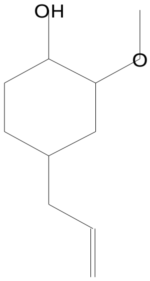


SI 789, RSI 803, replib, Entry# 25332, CAS# 97-53-0, Eugenol

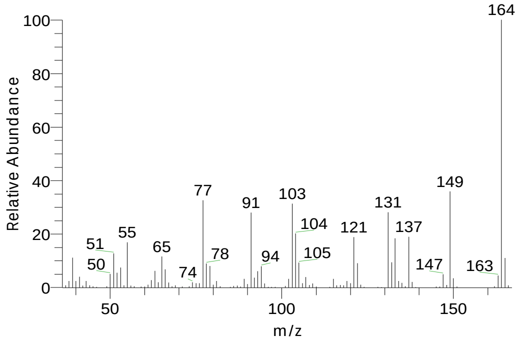


Clove_SayedMekky_DrSayed #3334 RT: 14.18 AV: 1 NL: 1.74E5
T: + c EI Full ms [50.000-500.000]

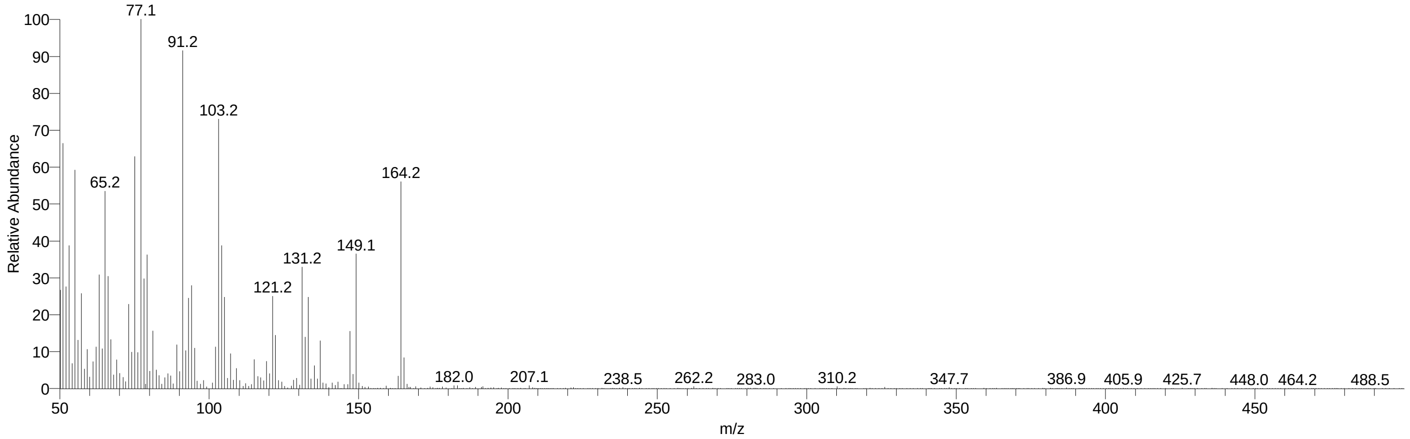


| RT | Compound Name | Area % | MF | Molecular Formula | Molecular Weight | Cas # | Library |
|---|---|---|---|---|---|---|---|
| 14.18 | Phenol, 2-methoxy-3-(2-propenyl)- | 5.23 | 806 | C10H12O2 | 164 | 1941-12-4 | mainlib |
| 14.18 | PHENOL, 2-METHOXY-3-(2-PROPENYL)- | 5.23 | 806 | C10H12O2 | 164 | 1941-12-4 | WileyRegistry8e |
| 14.18 | PHENOL, 2-METHOXY-4-(2-PROPENYL)- | 5.23 | 813 | C10H12O2 | 164 | 97-53-0 | WileyRegistry8e |
| 14.18 | PHENOL, 2-METHOXY-4-(2-PROPENYL)- | 5.23 | 810 | C10H12O2 | 164 | 97-53-0 | WileyRegistry8e |
| 14.18 | Eugenol | 5.23 | 802 | C10H12O2 | 164 | 97-53-0 | replib |

Compound Structure | Hit Spectrum

Phenol, 2-methoxy-3-(2-propenyl)-
Formula C10H12O2, MW 164, CAS# 1941-12-4, Entry# 153401
3-Allyl-2-methoxyphenol #

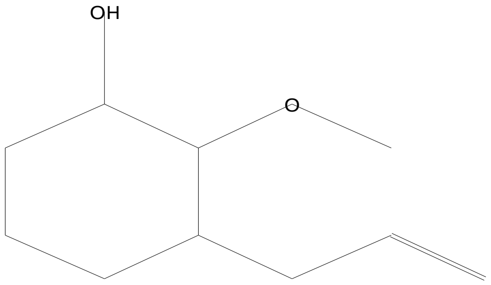


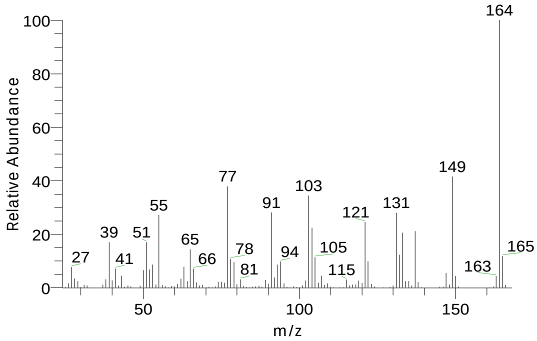

# *My GC-MS Report*

Compound Structure | Hit Spectrum

PHENOL, 2-METHOXY-3-(2-PROPENYL)-
Formula C10H12O2, MW 164, CAS# 1941-12-4, Entry# 390451
3-ALLYL-2-METHOXYPHENOL #

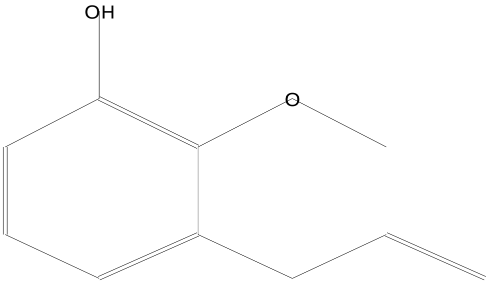


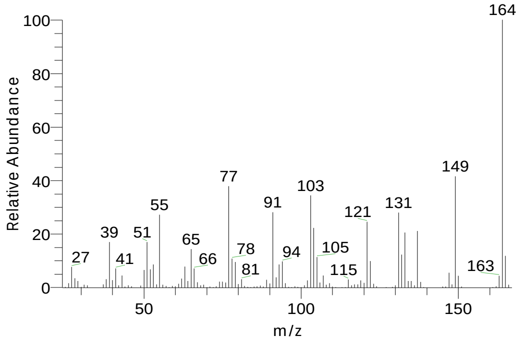


PHENOL, 2-METHOXY-4-(2-PROPENYL)-
Formula C10H12O2, MW 164, CAS# 97-53-0, Entry# 46886
4-ALLYL-2-METHOXY-PHENOL

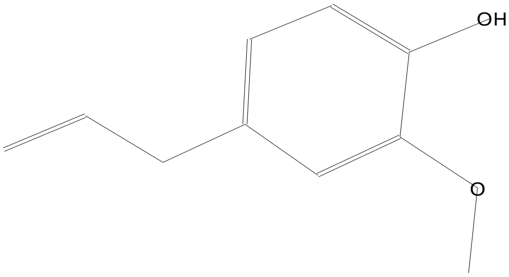


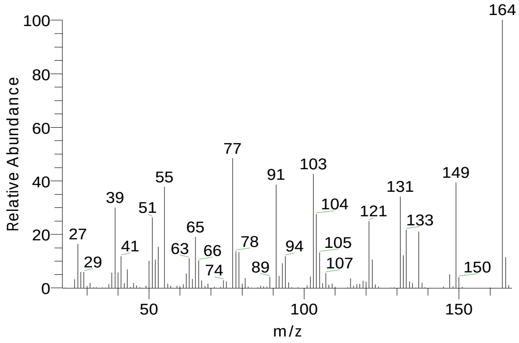


PHENOL, 2-METHOXY-4-(2-PROPENYL)-
Formula C10H12O2, MW 164, CAS# 97-53-0, Entry# 46893
4-ALLYL-2-METHOXY-PHENOL

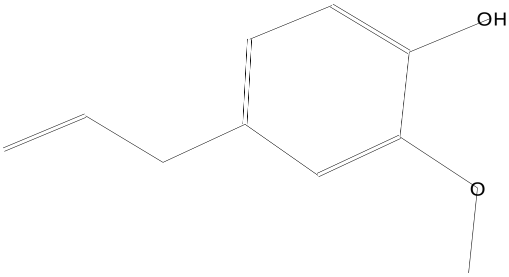


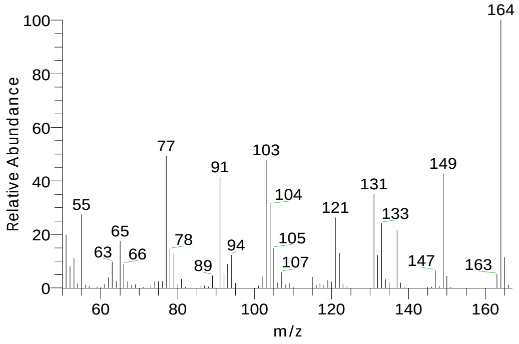


Eugenol
Formula C10H12O2, MW 164, CAS# 97-53-0, Entry# 25332
Phenol, 2-methoxy-4-(2-propenyl)-

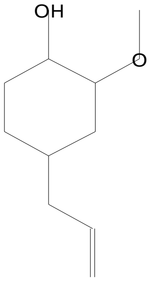


SI 788, RSI 802, replib, Entry# 25332, CAS# 97-53-0, Eugenol

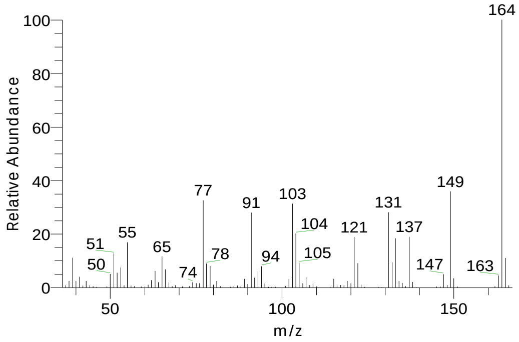

# My GC-MS Report

Clove_SayedMekky_DrSayed #3447 RT: 14.56 AV: 1 NL: 2.10E5
T: + c EI Full ms [50.000-500.000]

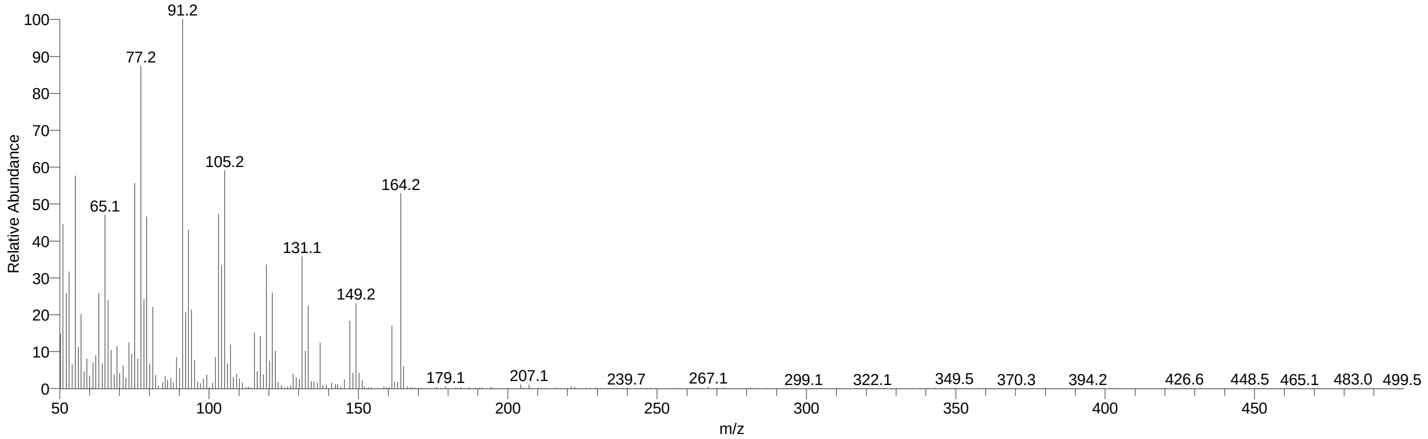


| RT | Compound Name | Area % | MF | Molecular Formula | Molecular Weight | Cas # | Library |
|---|---|---|---|---|---|---|---|
| 14.56 | Bicyclo[3.1.1]hept-3-ene-spiro-2,4'-(1',3'-dioxane), 7,7-dimethyl- | 1.15 | 763 | C12H18O2 | 194 | NA | mainlib |
| 14.56 | BICYCLO[3.1.1]HEPT-2-EN-6-SPIRO-4'-(1',3'-DIOXAN), 7,7-DIMETHYL- | 1.15 | 763 | C12H18O2 | 194 | NA | WileyRegistry8e |
| 14.56 | BICYCLO[3.1.1]HEPT-3-EN-2-SPIRO-4'-(1',3'-DIOXAN), 7,7-DIMETHYL- | 1.15 | 763 | C12H18O2 | 194 | NA | WileyRegistry8e |
| 14.56 | Phenol, 2-methoxy-4-(1-propenyl)- | 1.15 | 809 | C10H12O2 | 164 | 97-54-1 | mainlib |
| 14.56 | PHENOL, 2-METHOXY-4-(2-PROPENYL)- | 1.15 | 776 | C10H12O2 | 164 | 97-53-0 | WileyRegistry8e |

Compound Structure

Hit Spectrum

Bicyclo[3.1.1]hept-3-ene-spiro-2,4'-(1',3'-dioxane), 7,7-dimethyl-
Formula C12H18O2, MW 194, CAS# NA, Entry# 433
$:28CGUXJLVEFQACKH-UHFFFAOYSA-N

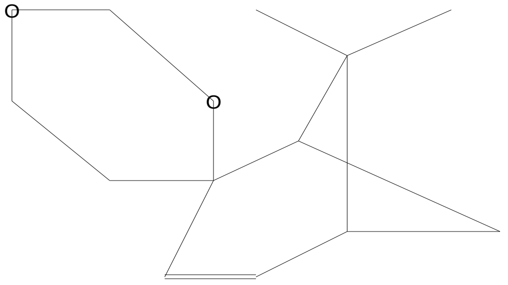


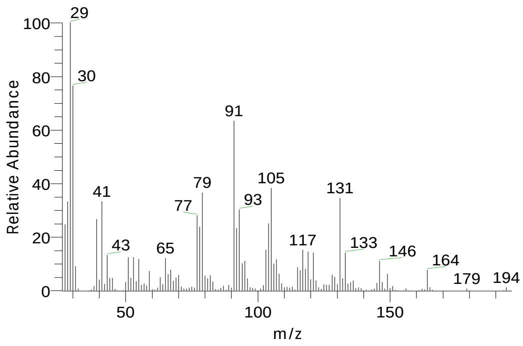


BICYCLO[3.1.1]HEPT-2-EN-6-SPIRO-4'-(1',3'-DIOXAN), 7,7-DIMETHYL-
Formula C12H18O2, MW 194, CAS# NA, Entry# 77632

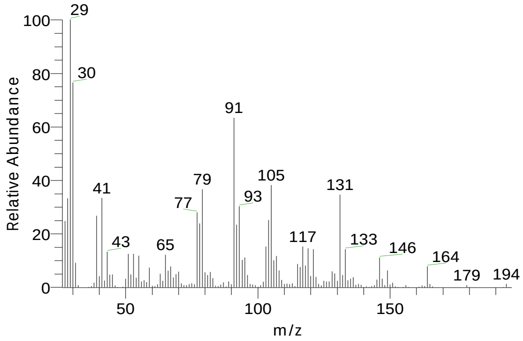

# *My GC-MS Report*

Compound Structure | Hit Spectrum

BICYCLO[3.1.1]HEPT-3-EN-2-SPIRO-4'-(1',3'-DIOXAN), 7,7-DIMETHYL-
Formula C12H18O2, MW 194, CAS# NA, Entry# 391066

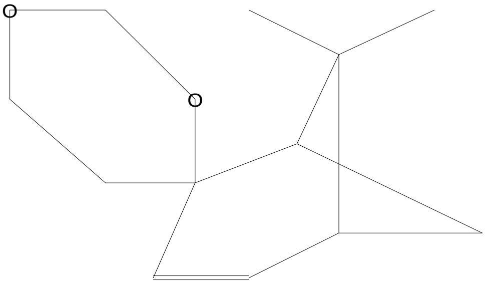


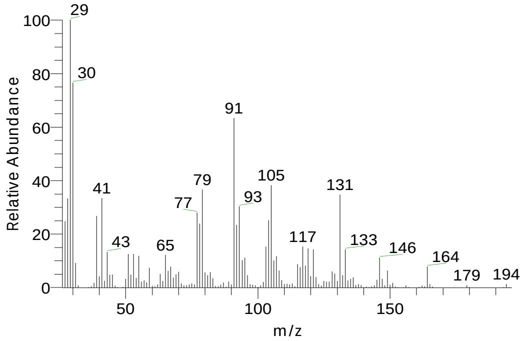


Phenol, 2-methoxy-4-(1-propenyl)-
Formula C10H12O2, MW 164, CAS# 97-54-1, Entry# 153098
Isoeugenol

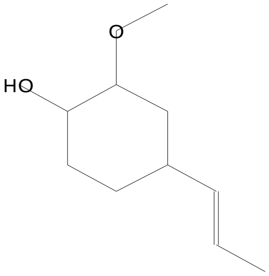


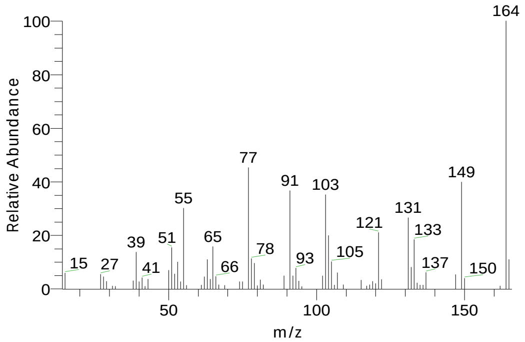


PHENOL, 2-METHOXY-4-(2-PROPENYL)-
Formula C10H12O2, MW 164, CAS# 97-53-0, Entry# 46886
4-ALLYL-2-METHOXY-PHENOL

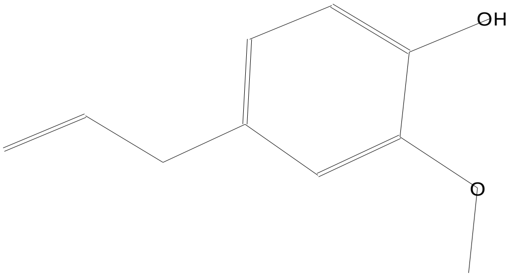


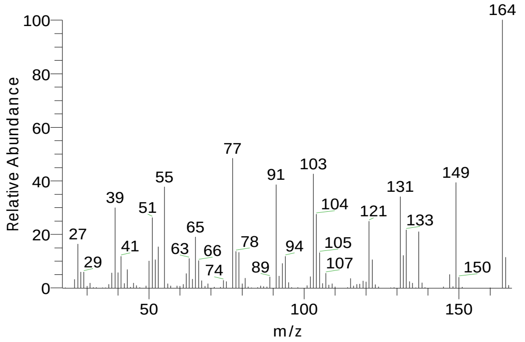


Clove_SayedMekky_DrSayed #3735 RT: 15.52 AV: 1 NL: 2.53E6
T: + c EI Full ms [50.000-500.000]

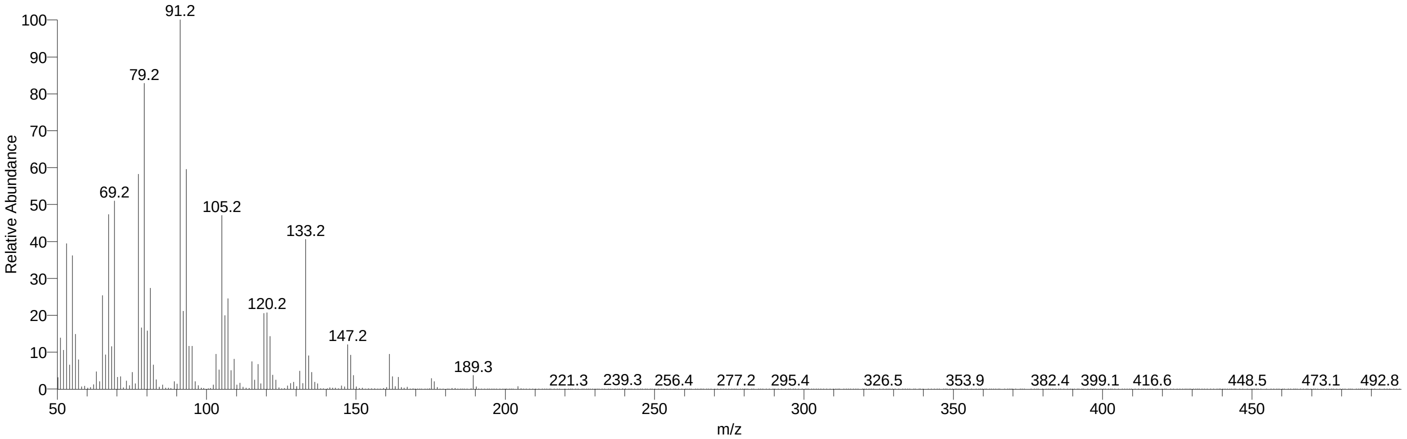


| RT | Compound Name | Area % | MF | Molecular Formula | Molecular Weight | Cas # | Library |
|---|---|---|---|---|---|---|---|
| 15.52 | BICYCLO[7.2.0]UNDEC-4-ENE, 4,11,11-TRIMETHYL-8-METHYLENE-, [1R-(1R*,4E,9S*)]- | 31.25 | 921 | C15H24 | 204 | 87-44-5 | WileyRegistry8e |

# *My GC-MS Report*

| RT | Compound Name | Area % | MF | Molecular Formula | Molecular Weight | Cas # | Library |
|---|---|---|---|---|---|---|---|
| 15.52 | BICYCLO[7.2.0]UNDEC-4-ENE, 4,11,11-TRIMETHYL-8-METHYLENE-, [1R-(1R*,4E,9S*)]- | 31.25 | 944 | C15H24 | 204 | 87-44-5 | WileyRegistry8e |
| 15.52 | BICYCLO[7.2.0]UNDEC-4-ENE, 4,11,11-TRIMETHYL-8-METHYLENE-, [1R-(1R*,4E,9S*)]- | 31.25 | 908 | C15H24 | 204 | 87-44-5 | WileyRegistry8e |
| 15.52 | BICYCLO[7.2.0]UNDEC-4-ENE, 4,11,11-TRIMETHYL-8-METHYLENE-, [1R-(1R*,4E,9S*)]- | 31.25 | 905 | C15H24 | 204 | 87-44-5 | WileyRegistry8e |
| 15.52 | BICYCLO[7.2.0]UNDEC-4-ENE, 4,11,11-TRIMETHYL-8-METHYLENE-, [1R-(1R*,4E,9S*)]- | 31.25 | 910 | C15H24 | 204 | 87-44-5 | WileyRegistry8e |

| Compound Structure | Hit Spectrum |
|---|---|

BICYCLO[7.2.0]UNDEC-4-ENE, 4,11,11-TRIMETHYL-8-METHYLENE-, [1R-(1R*,4E,9S*)]-
Formula C15H24, MW 204, CAS# 87-44-5, Entry# 89161
2,6,10,10-TETRAMETHYLBICYCLO[7.2.0]UNDECA-1,6-DIENE #

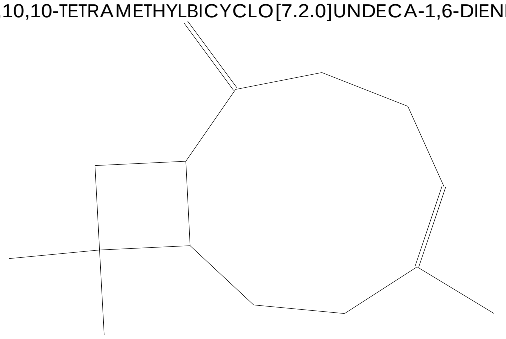

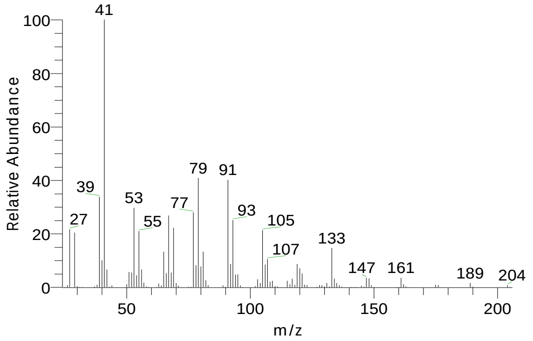


BICYCLO[7.2.0]UNDEC-4-ENE, 4,11,11-TRIMETHYL-8-METHYLENE-, [1R-(1R*,4E,9S*)]-
Formula C15H24, MW 204, CAS# 87-44-5, Entry# 89167
2,6,10,10-TETRAMETHYLBICYCLO[7.2.0]UNDECA-1,6-DIENE #

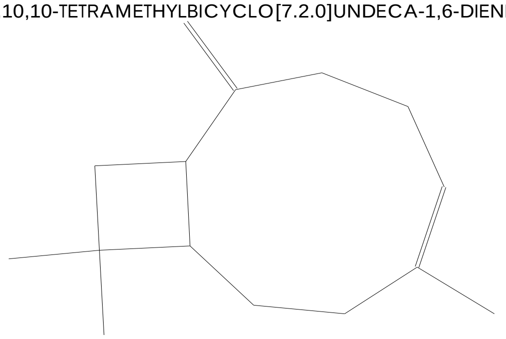

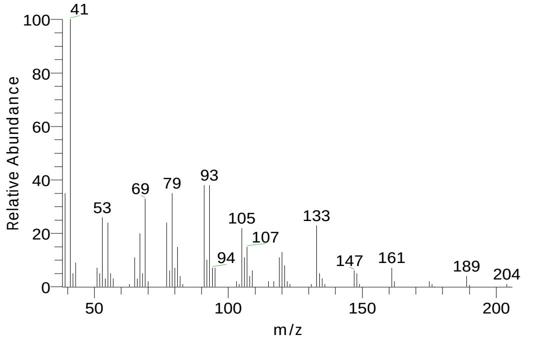


BICYCLO[7.2.0]UNDEC-4-ENE, 4,11,11-TRIMETHYL-8-METHYLENE-, [1R-(1R*,4E,9S*)]-
Formula C15H24, MW 204, CAS# 87-44-5, Entry# 89162
2,6,10,10-TETRAMETHYLBICYCLO[7.2.0]UNDECA-1,6-DIENE #

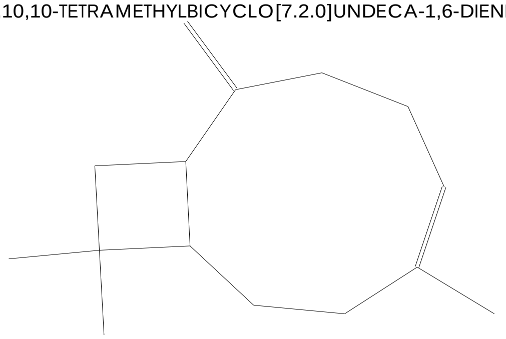

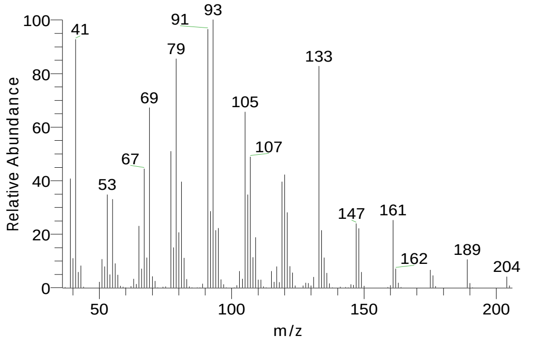

# My GC-MS Report

Compound Structure | Hit Spectrum

BICYCLO[7.2.0]UNDEC-4-ENE, 4,11,11-TRIMETHYL-8-METHYLENE-, [1R-(1R*,4E,9S*)]-
Formula C15H24, MW 204, CAS# 87-44-5, Entry# 89166
2,6,10,10-TETRAMETHYLBICYCLO[7.2.0]UNDECA-1,6-DIENE #

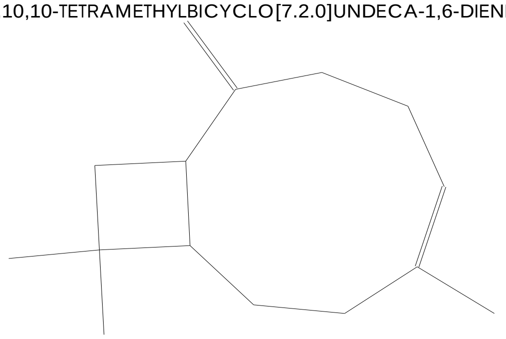

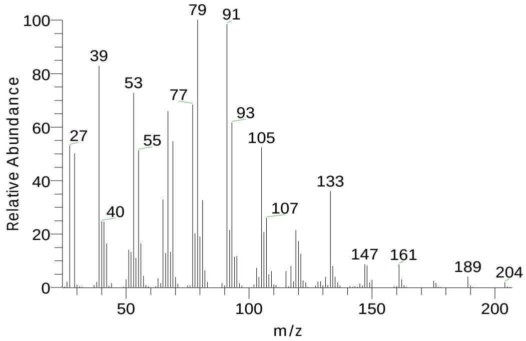


BICYCLO[7.2.0]UNDEC-4-ENE, 4,11,11-TRIMETHYL-8-METHYLENE-, [1R-(1R*,4E,9S*)]-
Formula C15H24, MW 204, CAS# 87-44-5, Entry# 89170
2,6,10,10-TETRAMETHYLBICYCLO[7.2.0]UNDECA-1,6-DIENE #

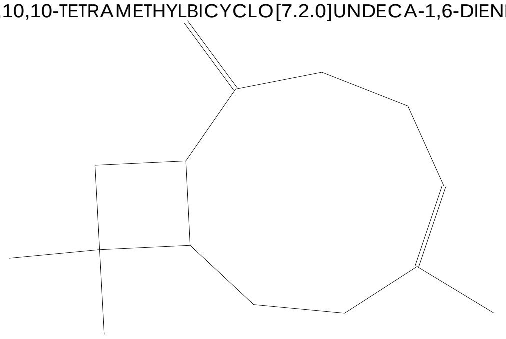

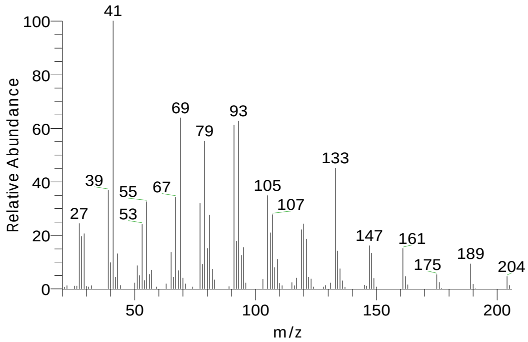


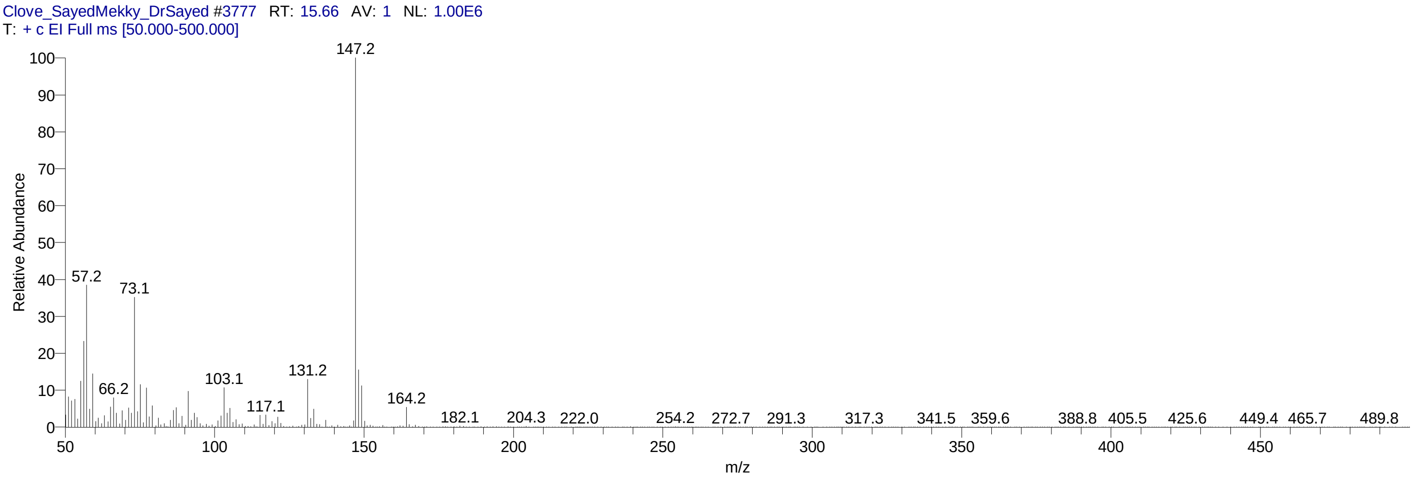


| RT | Compound Name | Area % | MF | Molecular Formula | Molecular Weight | Cas # | Library |
|---|---|---|---|---|---|---|---|
| 15.66 | 2-Dimethyl(trimethylsilyl)silyloxytetradecane | 2.30 | 773 | C19H44OSi2 | 344 | NA | mainlib |
| 15.66 | 2-Dimethyl(trimethylsilyl)silyloxytridecane | 2.30 | 765 | C18H42OSi2 | 330 | NA | mainlib |
| 15.66 | 7-Dimethyl(trimethylsilyl)silyloxytetradecane | 2.30 | 761 | C19H44OSi2 | 344 | NA | mainlib |
| 15.66 | 5-Dimethyl(trimethylsilyl)silyloxytridecane | 2.30 | 753 | C18H42OSi2 | 330 | NA | mainlib |
| 15.66 | Disiloxane, hexamethyl- | 2.30 | 774 | C6H18OSi2 | 162 | 107-46-0 | replib |

# My GC-MS Report

Compound Structure | Hit Spectrum

2-Dimethyl(trimethylsilyl)silyloxytetradecane
Formula C19H44OSi2, MW 344, CAS# NA, Entry# 135021
1,1,1,2,2-Pentamethyl-2-[(1-methyltridecyl)oxy]disilane #

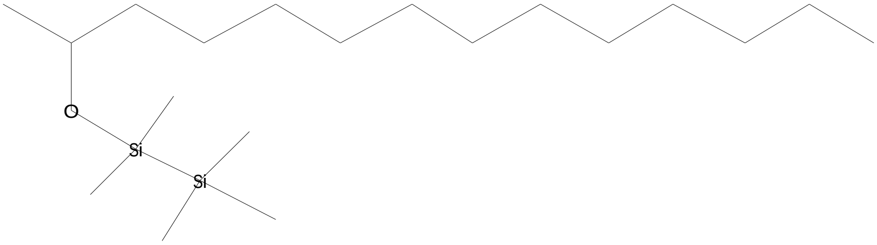


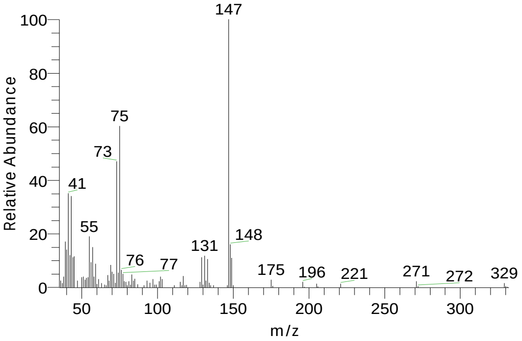


2-Dimethyl(trimethylsilyl)silyloxytridecane
Formula C18H42OSi2, MW 330, CAS# NA, Entry# 135017
1,1,1,2,2-Pentamethyl-2-[(1-methyldodecyl)oxy]disilane #

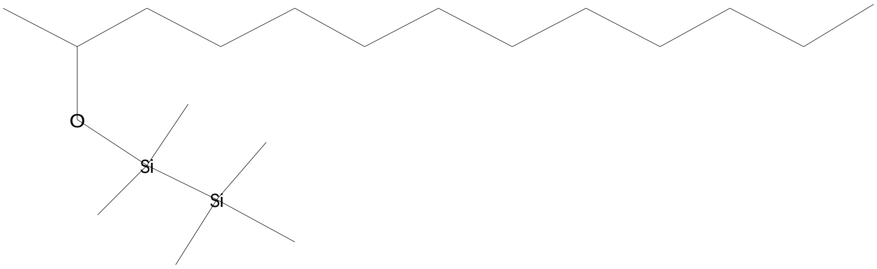


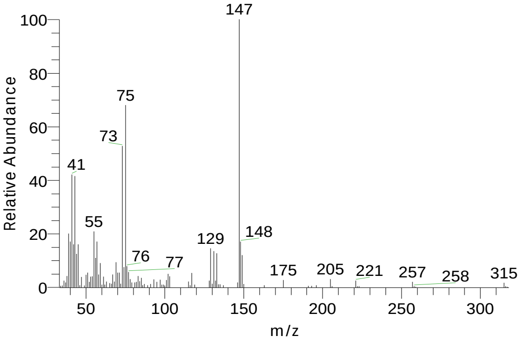


7-Dimethyl(trimethylsilyl)silyloxytetradecane
Formula C19H44OSi2, MW 344, CAS# NA, Entry# 135025
1-[(1-Hexyloctyl)oxy]-1,1,2,2,2-pentamethyldisilane #

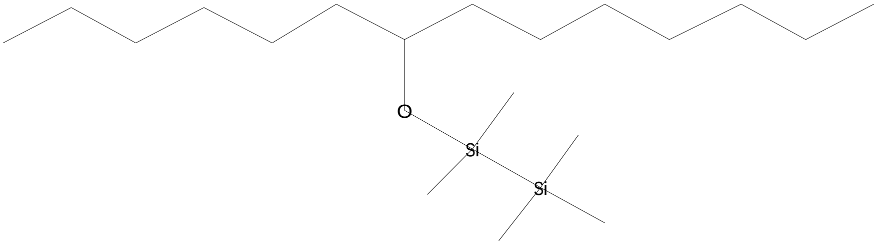


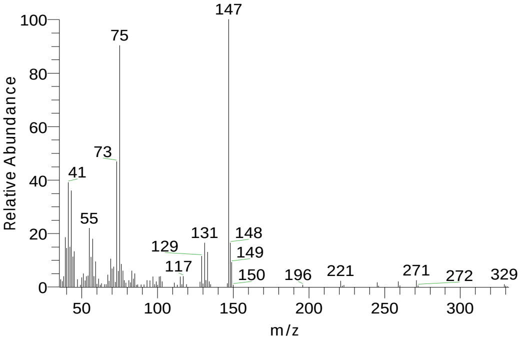


5-Dimethyl(trimethylsilyl)silyloxytridecane
Formula C18H42OSi2, MW 330, CAS# NA, Entry# 135020
1-[(1-Butylnonyl)oxy]-1,1,2,2,2-pentamethyldisilane #

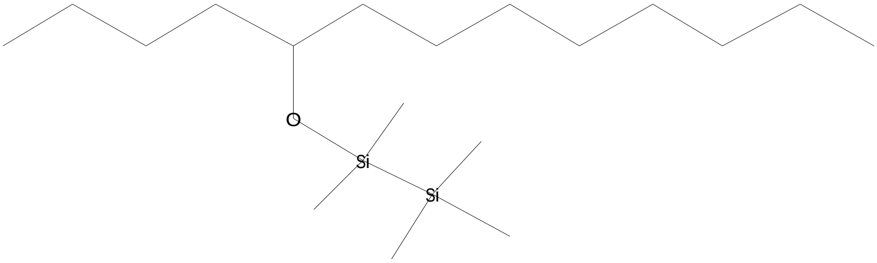


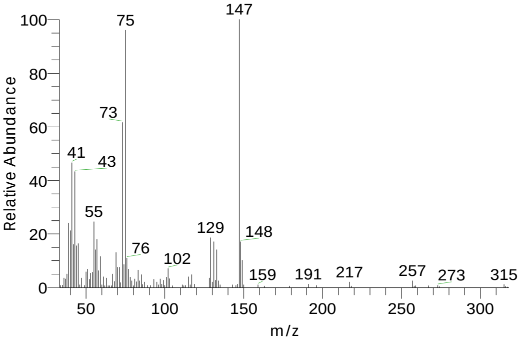

# My GC-MS Report

Compound Structure | Hit Spectrum

Disiloxane, hexamethyl-
Formula C6H18OSi2, MW 162, CAS# 107-46-0, Entry# 23139
Bis(trimethylsilyl) ether

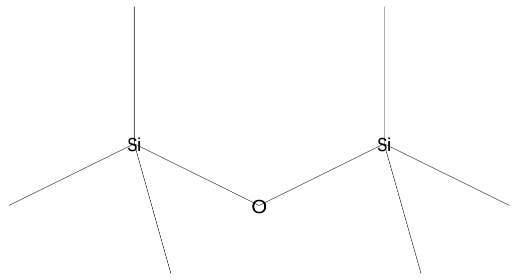


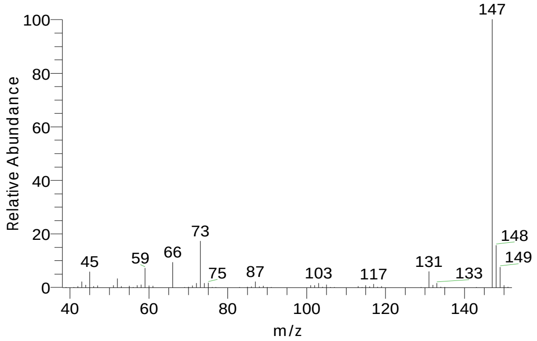


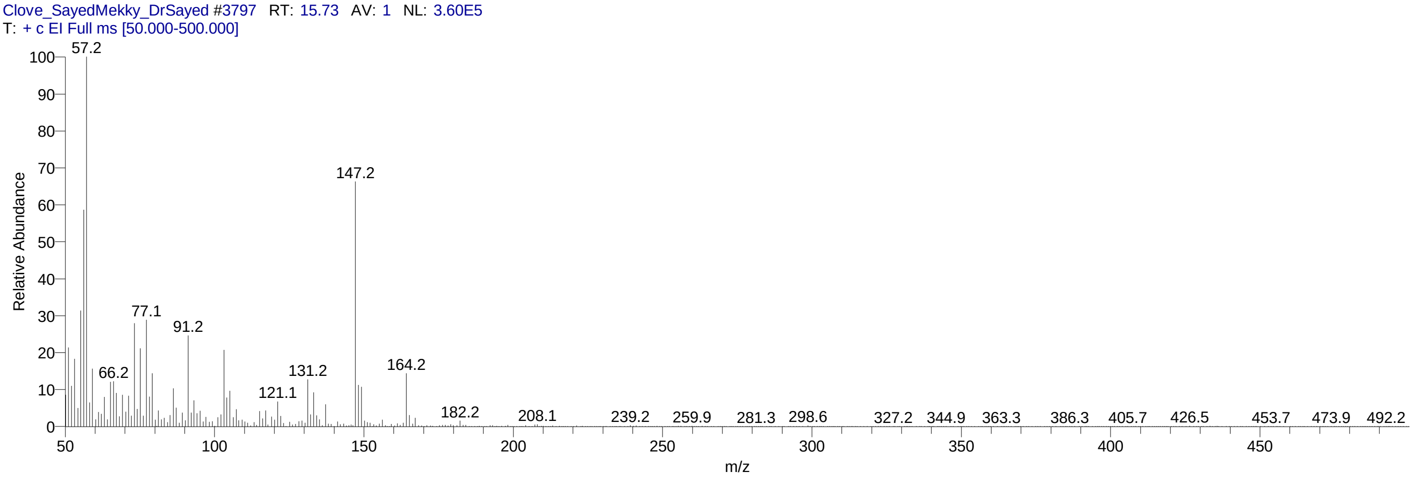


| RT | Compound Name | Area % | MF | Molecular Formula | Molecular Weight | Cas # | Library |
|---|---|---|---|---|---|---|---|
| 15.73 | 2-PROPENOIC ACID, 3-PHENYL- | 1.01 | 705 | C9H8O2 | 148 | 621-82-9 | WileyRegistry8e |
| 15.73 | PHENOL, 2-METHOXY-4-(2-PROPENYL)- | 1.01 | 697 | C10H12O2 | 164 | 97-53-0 | WileyRegistry8e |
| 15.73 | 2-PROPENOIC ACID, 3-PHENYL | 1.01 | 672 | C9H8O2 | 148 | NA | WileyRegistry8e |
| 15.73 | 2-Propenoic acid, 3-phenyl- | 1.01 | 671 | C9H8O2 | 148 | 621-82-9 | replib |
| 15.73 | Phenol, 2-methoxy-3-(2-propenyl)- | 1.01 | 627 | C10H12O2 | 164 | 1941-12-4 | mainlib |

Compound Structure | Hit Spectrum

2-PROPENOIC ACID, 3-PHENYL-
Formula C9H8O2, MW 148, CAS# 621-82-9, Entry# 32048
CINNAMIC ACID

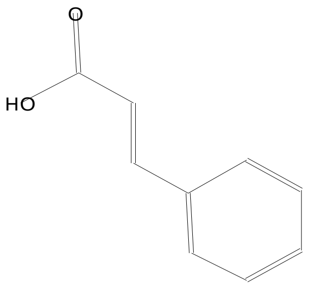


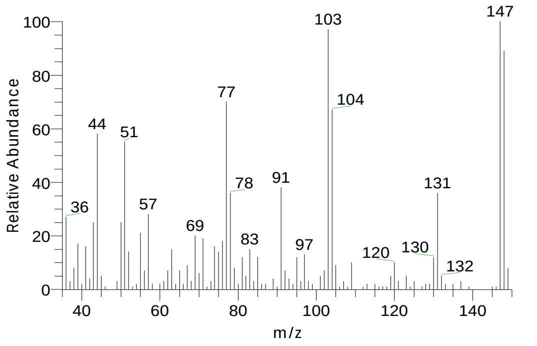

# *My GC-MS Report*

Compound Structure | Hit Spectrum

PHENOL, 2-METHOXY-4-(2-PROPENYL)-
Formula C10H12O2, MW 164, CAS# 97-53-0, Entry# 46895
4-ALLYL-2-METHOXY-PHENOL

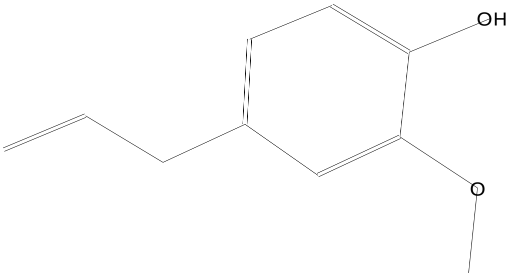


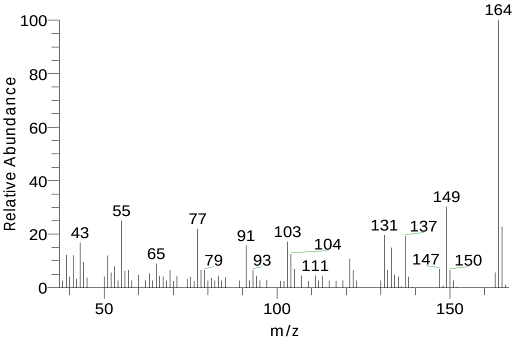


2-PROPENOIC ACID, 3-PHENYL
Formula C9H8O2, MW 148, CAS# NA, Entry# 356885
3-PHENYL-2-PROPENOIC ACID

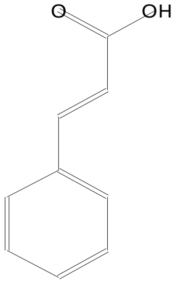


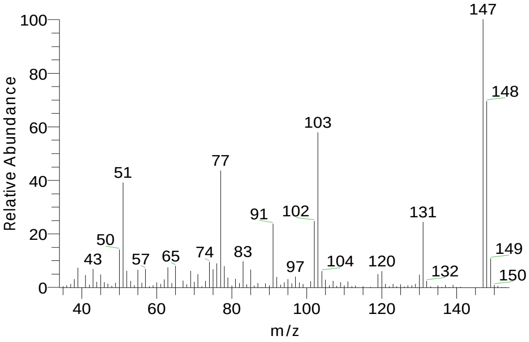


2-Propenoic acid, 3-phenyl-
Formula C9H8O2, MW 148, CAS# 621-82-9, Entry# 23249
Cinnamic acid

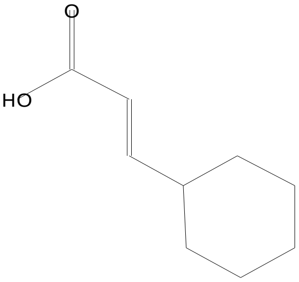


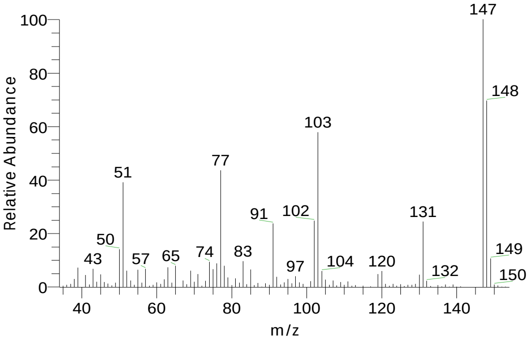


Phenol, 2-methoxy-3-(2-propenyl)-
Formula C10H12O2, MW 164, CAS# 1941-12-4, Entry# 153401
3-Allyl-2-methoxyphenol #

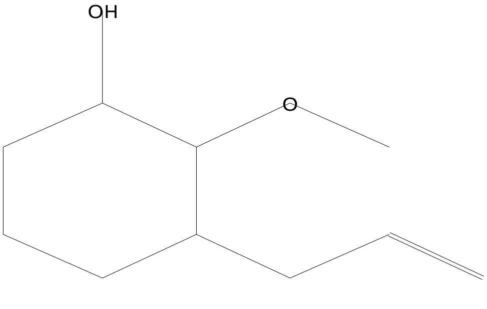


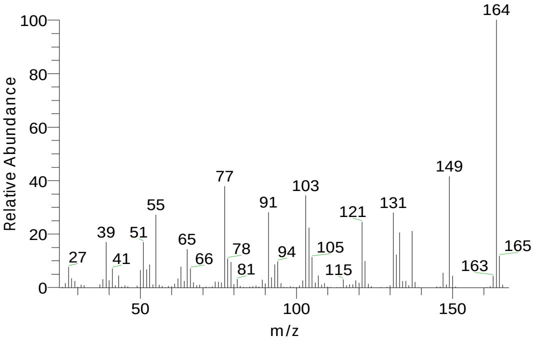

# *My GC-MS Report*

Clove_SayedMekky_DrSayed #3817 RT: 15.80 AV: 1 NL: 3.63E5
T: + c EI Full ms [50.000-500.000]

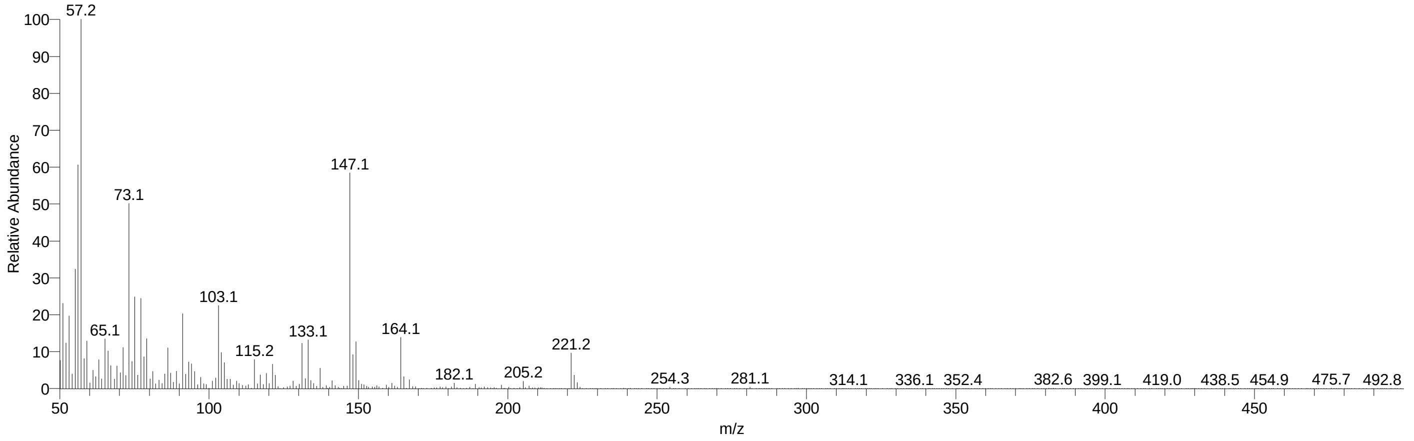


| RT | Compound Name | Area % | MF | Molecular Formula | Molecular Weight | Cas # | Library |
|---|---|---|---|---|---|---|---|
| 15.80 | 9,12,15-OCTADECATRIENOIC ACID, 2-[(TRIMETHYLSILYL)OXY]-1-[[(TRIMETHYLSILYL)OXY]METHYL]ETHYL ESTER, (Z,Z,Z)- | 1.86 | 695 | C27H52O4Si2 | 496 | 55521-23-8 | WileyRegistry8e |
| 15.80 | 2-Dimethyl(trimethylsilyl)silyloxytridecane | 1.86 | 741 | C18H42OSi2 | 330 | NA | mainlib |
| 15.80 | 2-Dimethyl(trimethylsilyl)silyloxytetradecane | 1.86 | 745 | C19H44OSi2 | 344 | NA | mainlib |
| 15.80 | 3-Dimethyl(trimethylsilyl)silyloxytetradecane | 1.86 | 749 | C19H44OSi2 | 344 | NA | mainlib |
| 15.80 | 5-Dimethyl(trimethylsilyl)silyloxytridecane | 1.86 | 734 | C18H42OSi2 | 330 | NA | mainlib |

Compound Structure | Hit Spectrum

Formula C27H52O4Si2, MW 496, CAS# 55521-23-8, Entry# 284835

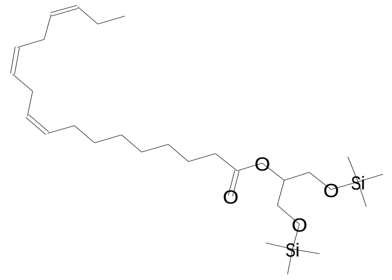

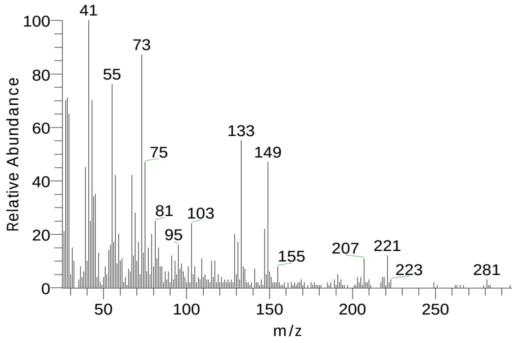


2-Dimethyl(trimethylsilyl)silyloxytridecane
Formula C18H42OSi2, MW 330, CAS# NA, Entry# 135017
1,1,1,2,2-Pentamethyl-2-[(1-methyldodecyl)oxy]disilane #

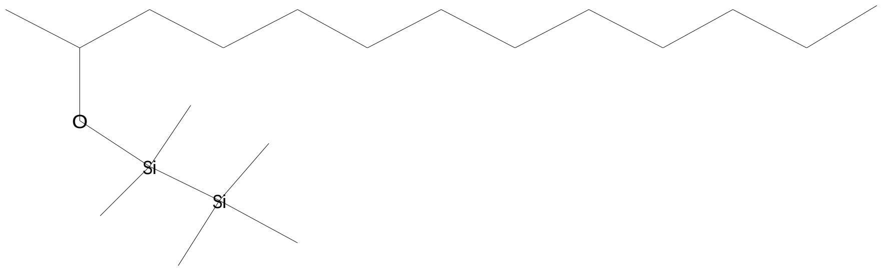

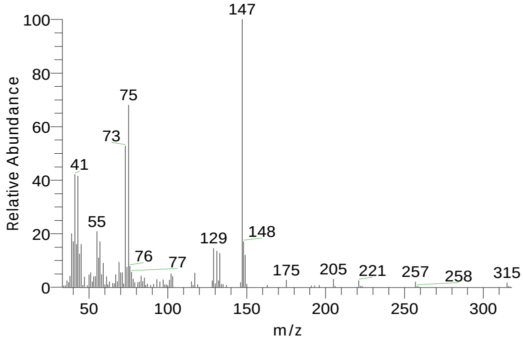

# My GC-MS Report

| Compound Structure | Hit Spectrum |
|---|---|

2-Dimethyl(trimethylsilyl)silyloxytetradecane
Formula C19H44OSi2, MW 344, CAS# NA, Entry# 135021
1,1,1,2,2-Pentamethyl-2-[(1-methyltridecyl)oxy]disilane #

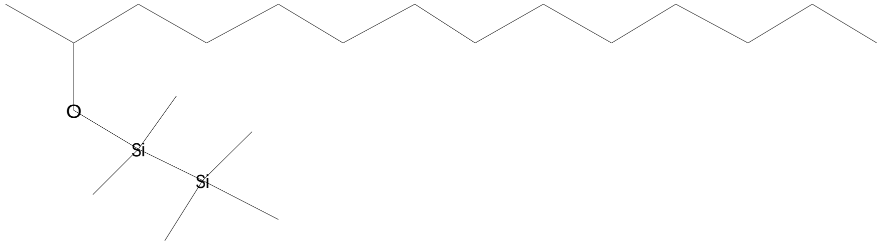


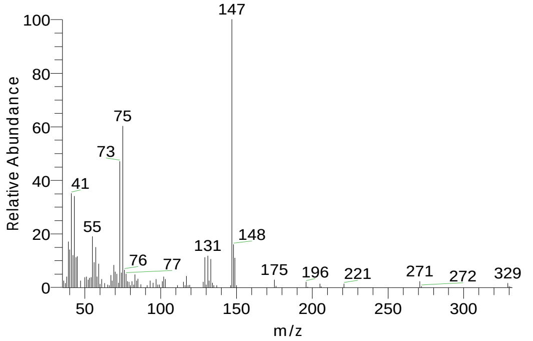


3-Dimethyl(trimethylsilyl)silyloxytetradecane
Formula C19H44OSi2, MW 344, CAS# NA, Entry# 135022
1-[(1-Ethyldodecyl)oxy]-1,1,2,2,2-pentamethyldisilane #

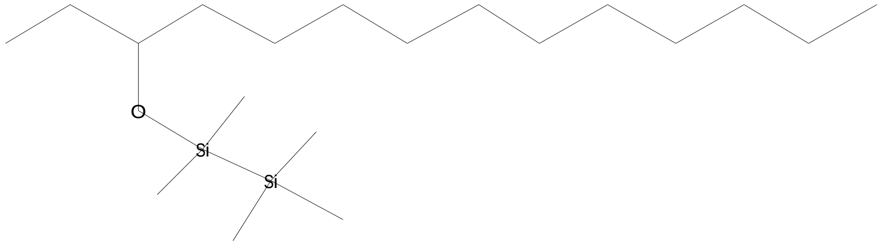


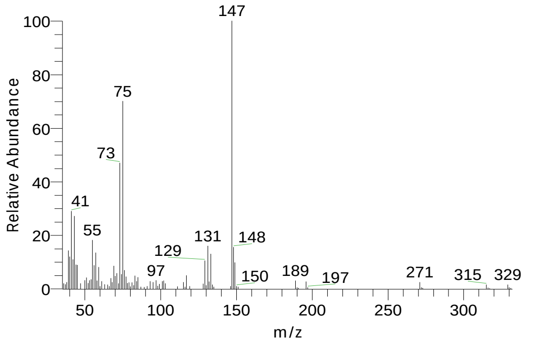


5-Dimethyl(trimethylsilyl)silyloxytridecane
Formula C18H42OSi2, MW 330, CAS# NA, Entry# 135020
1-[(1-Butylnonyl)oxy]-1,1,2,2,2-pentamethyldisilane #

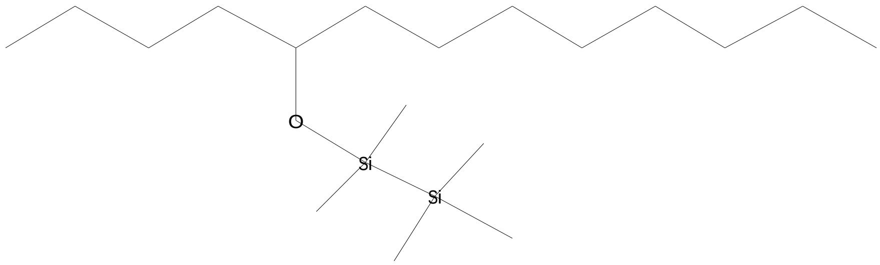


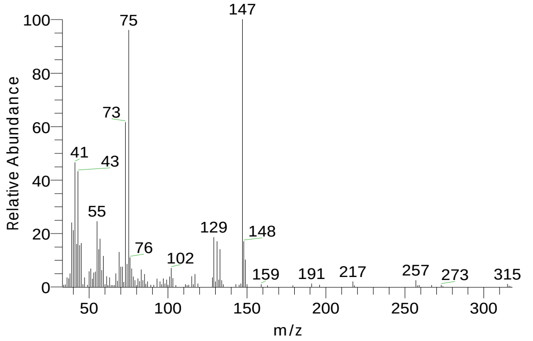


Clove_SayedMekky_DrSayed #3972 RT: 16.32 AV: 1 NL: 3.35E5
T: + c EI Full ms [50.000-500.000]

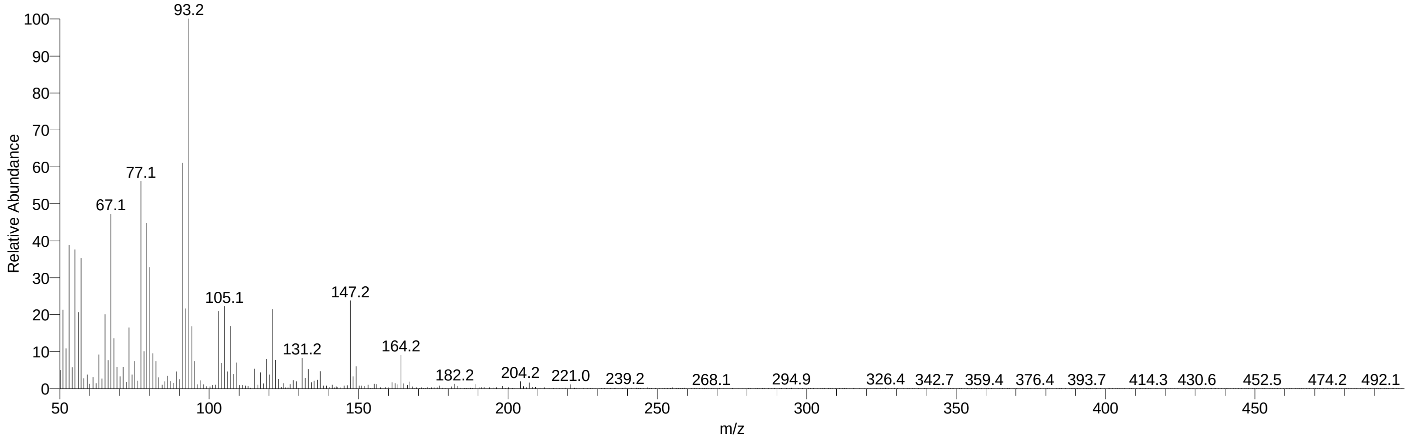


| RT | Compound Name | Area % | MF | Molecular Formula | Molecular Weight | Cas # | Library |
|---|---|---|---|---|---|---|---|
| 16.32 | 1,4,8-CYCLOUNDECATRIENE, 2,6,6,9-TETRAMETHYL-, (E,E,E)- | 2.73 | 819 | C15H24 | 204 | 6753-98-6 | WileyRegistry8e |
| 16.32 | 1,4,8-CYCLOUNDECATRIENE, 2,6,6,9-TETRAMETHYL-, (E,E,E)- | 2.73 | 811 | C15H24 | 204 | 6753-98-6 | WileyRegistry8e |

# *My GC-MS Report*

| RT | Compound Name | Area % | MF | Molecular Formula | Molecular Weight | Cas # | Library |
|---|---|---|---|---|---|---|---|
| 16.32 | Humulene | 2.73 | 843 | C15H24 | 204 | 6753-98-6 | replib |
| 16.32 | cis-à-Bisabolene | 2.73 | 808 | C15H24 | 204 | 29837-07-8 | mainlib |
| 16.32 | Cyclohexene, 4-isopropenyl-1-methoxymethoxymethyl- | 2.73 | 758 | C12H20O2 | 196 | NA | mainlib |

| Compound Structure | Hit Spectrum |
|---|---|

1,4,8-CYCLOUNDECATRIENE, 2,6,6,9-TETRAMETHYL-, (E,E,E)-
Formula C15H24, MW 204, CAS# 6753-98-6, Entry# 89115
2,6,6,9-TETRAMETHYL-1,4,8-CYCLOUNDECATRIENE #

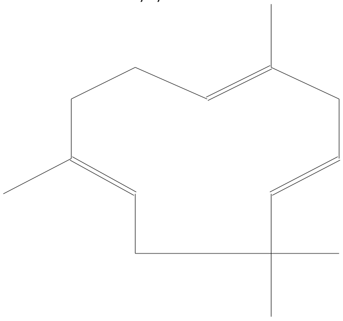

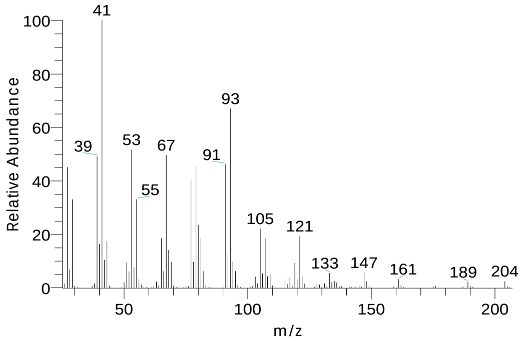


1,4,8-CYCLOUNDECATRIENE, 2,6,6,9-TETRAMETHYL-, (E,E,E)-
Formula C15H24, MW 204, CAS# 6753-98-6, Entry# 89116
2,6,6,9-TETRAMETHYL-1,4,8-CYCLOUNDECATRIENE #

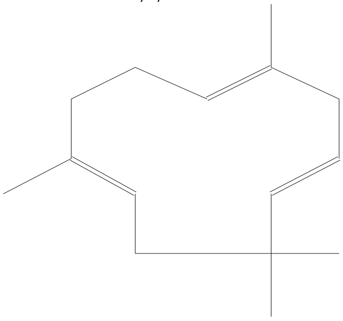

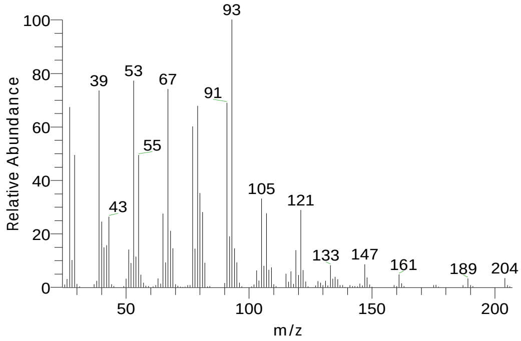


Humulene
Formula C15H24, MW 204, CAS# 6753-98-6, Entry# 14003
à-Caryophyllene

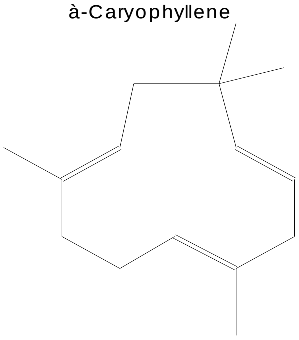

SI 768, RSI 843, replib, Entry# 14003, CAS# 6753-98-6, Humulene

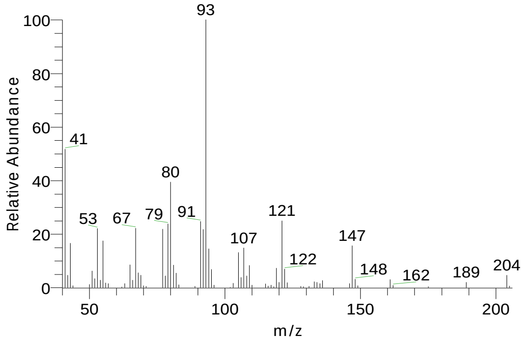


cis-à-Bisabolene
Formula C15H24, MW 204, CAS# 29837-07-8, Entry# 65792
4-[(1Z)-1,5-Dimethyl-1,4-hexadienyl]-1-methyl-1-cyclohexene

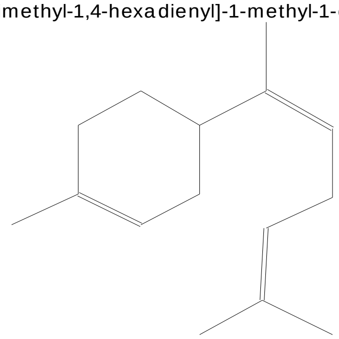

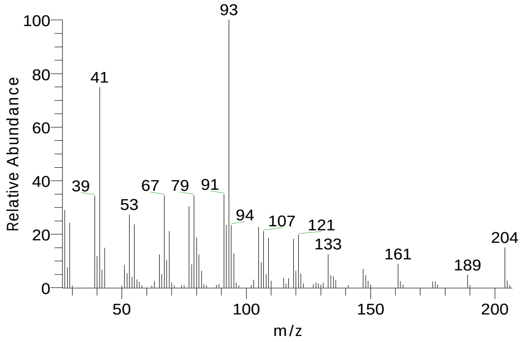

# My GC-MS Report

Compound Structure | Hit Spectrum

Cyclohexene, 4-isopropenyl-1-methoxymethoxymethyl-
Formula C12H20O2, MW 196, CAS# NA, Entry# 17436
4-Isopropenyl-1-[(methoxymethoxy)methyl]-1-cyclohexene #

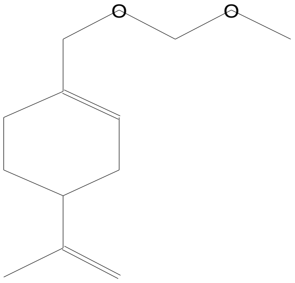

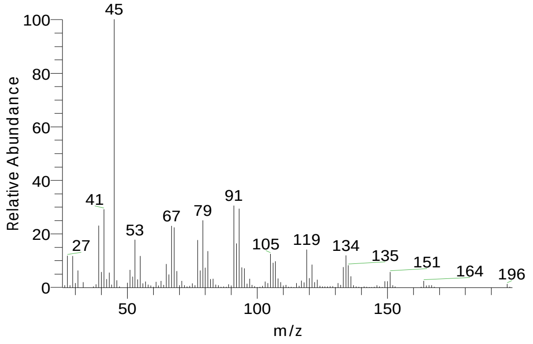


Clove_SayedMekky_DrSayed #4122 RT: 16.82 AV: 1 NL: 1.64E5
T: + c EI Full ms [50.000-500.000]

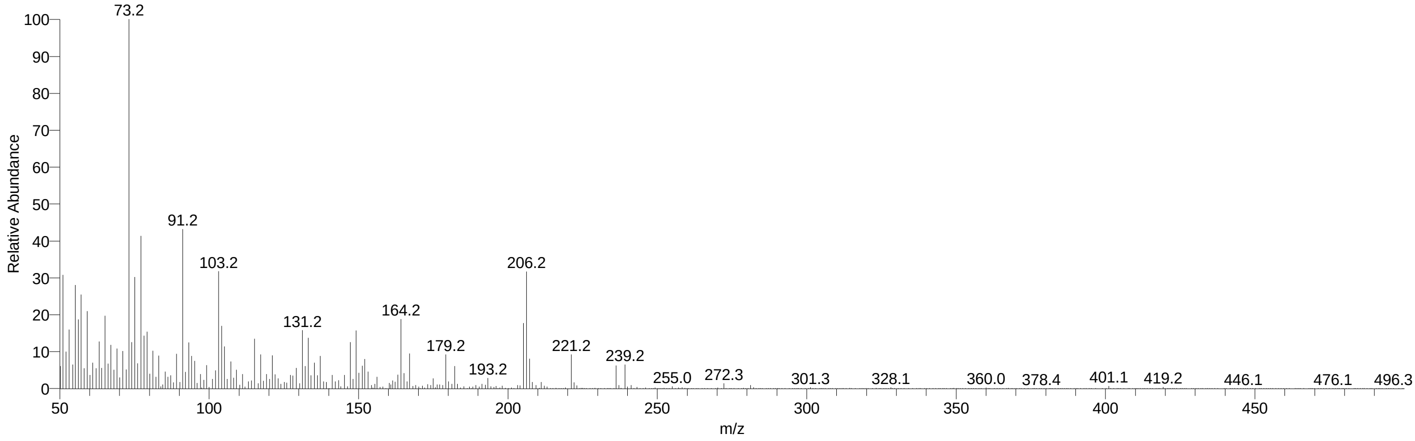


| RT | Compound Name | Area % | MF | Molecular Formula | Molecular Weight | Cas # | Library |
|---|---|---|---|---|---|---|---|
| 16.82 | 9,12,15-OCTADECATRIENOIC ACID, 2-[(TRIMETHYLSILYL)OXY]-1-[[(TRIMETHYLSILYL)OXY]METHYL]ETHYL ESTER, (Z,Z,Z)- | 1.28 | 730 | C27H52O4Si2 | 496 | 55521-23-8 | WileyRegistry8e |
| 16.82 | Eugenol, TMS derivative | 1.28 | 623 | C13H20O2Si | 236 | NA | mainlib |
| 16.82 | à-Phenethyl cyanide, 2-methoxy-6-nitro- | 1.28 | 687 | C10H10N2O3 | 206 | NA | mainlib |
| 16.82 | Cystathionine, 2TMS derivative | 1.28 | 598 | C13H30N2O4SSi2 | 366 | 73090-79-6 | replib |
| 16.82 | TRIMETHYLSILYL 2-AMINO-4-((2-AMINO-3-OXO-3-[(TRIMETHYLSILYL)OXY]PROPYL)SULFANYL)BUTANOATE # | 1.28 | 598 | C13H30N2O4SSi2 | 366 | 73090-79-6 | WileyRegistry8e |

Compound Structure | Hit Spectrum

Formula C27H52O4Si2, MW 496, CAS# 55521-23-8, Entry# 284835

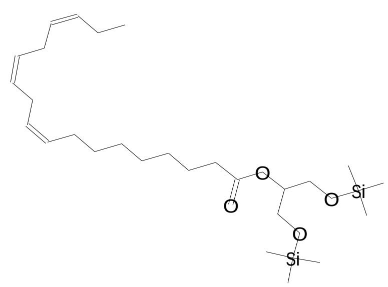

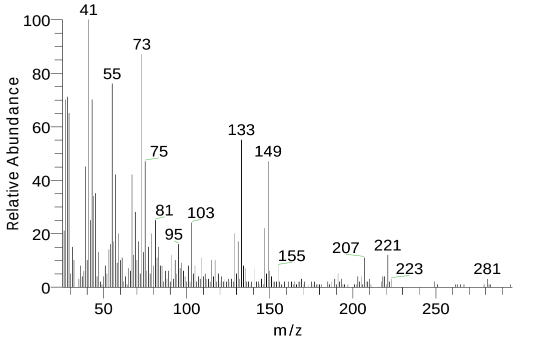

# *My GC-MS Report*

Compound Structure | Hit Spectrum

Eugenol, TMS derivative
Formula C13H20O2Si, MW 236, CAS# NA, Entry# 188517
(4-Allyl-2-methoxyphenoxy)trimethylsilane

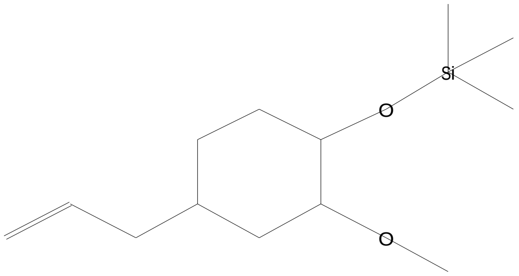


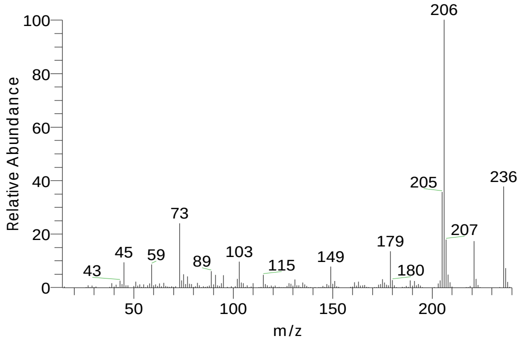


à-Phenethyl cyanide, 2-methoxy-6-nitro-
Formula C10H10N2O3, MW 206, CAS# NA, Entry# 46835
2-(2-Methoxy-6-nitrophenyl)propanenitrile #

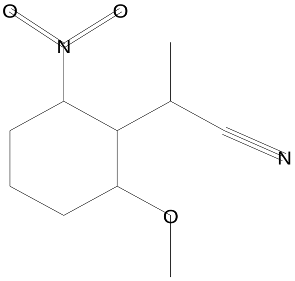


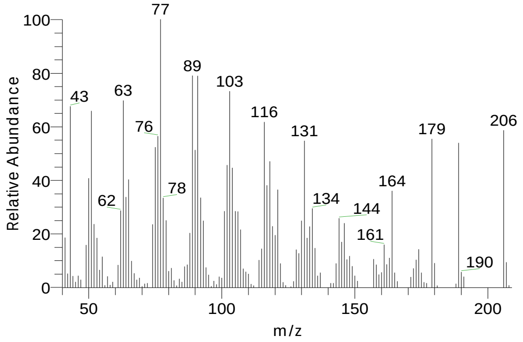


Cystathionine, 2TMS derivative
Formula C13H30N2O4SSi2, MW 366, CAS# 73090-79-6, Entry# 23126
Cystathionine, bis(triemthylsilyl) ester

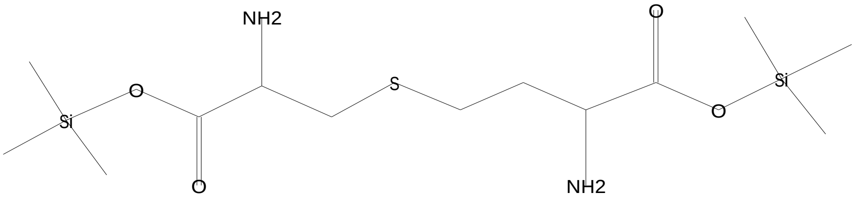


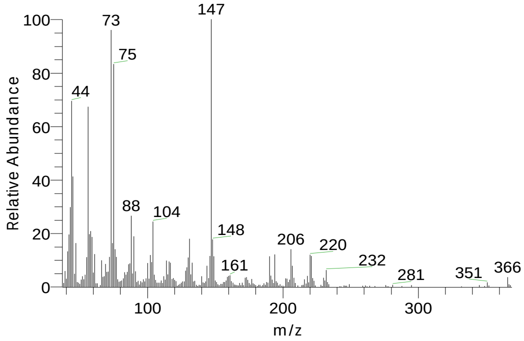


Formula C13H30N2O4SSi2, MW 366, CAS# 73090-79-6, Entry# 238204
CYSTATHIONINE DI-TMS

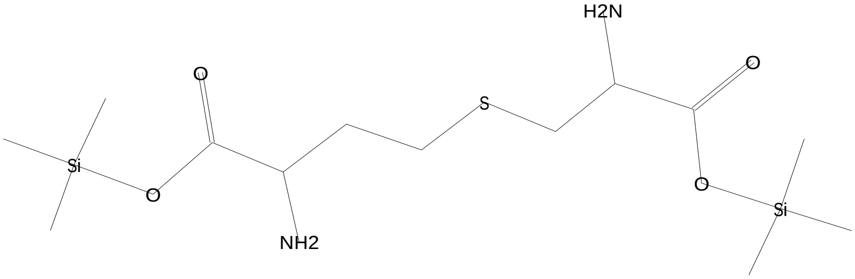


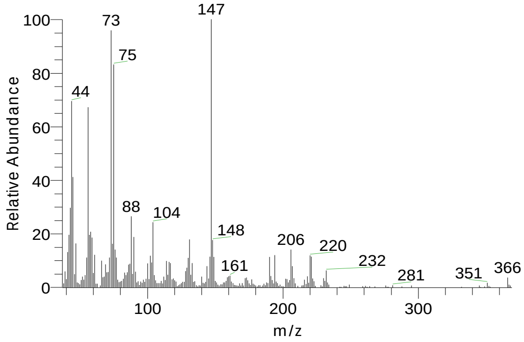

# My GC-MS Report

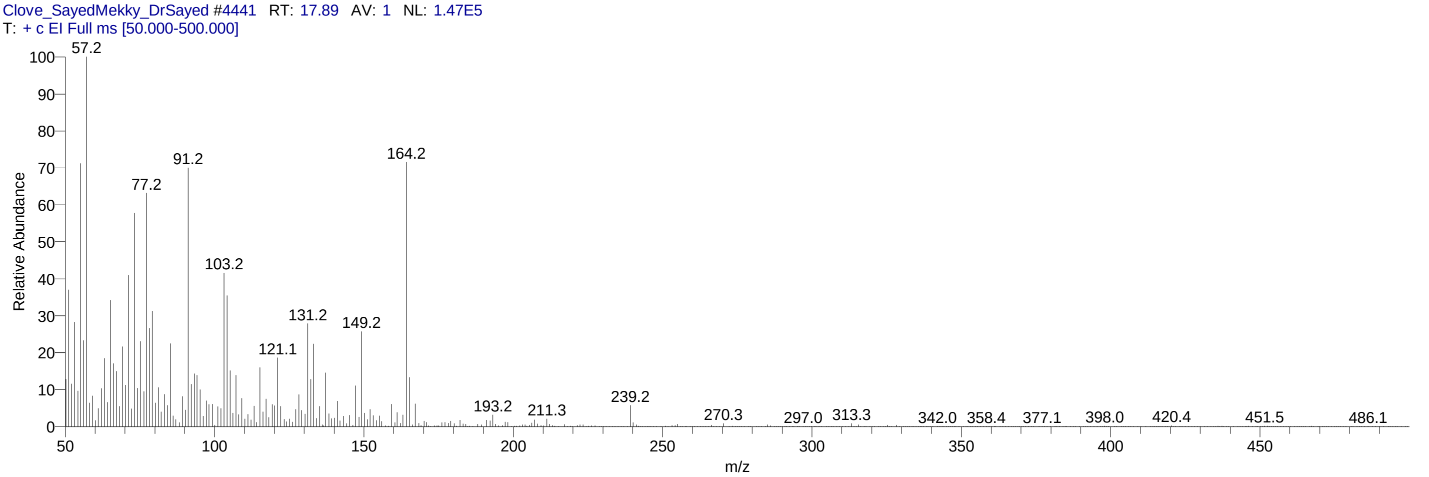


| RT | Compound Name | Area % | MF | Molecular Formula | Molecular Weight | Cas # | Library |
|---|---|---|---|---|---|---|---|
| 17.89 | PHENOL, 2-METHOXY-4-(2-PROPENYL)- | 1.62 | 823 | C10H12O2 | 164 | 97-53-0 | WileyRegistry8e |
| 17.89 | PHENOL, 2-METHOXY-4-(1-PROPENYL)- | 1.62 | 805 | C10H12O2 | 164 | 97-54-1 | WileyRegistry8e |
| 17.89 | PHENOL, 2-METHOXY-5-(1-PROPENYL)-, (E)- | 1.62 | 734 | C10H12O2 | 164 | 19784-98-6 | WileyRegistry8e |
| 17.89 | 10-Heptadecen-8-ynoic acid, methyl ester, (E)- | 1.62 | 707 | C18H30O2 | 278 | 16714-85-5 | mainlib |
| 17.89 | 10-HEPTADECEN-8-YNOIC ACID, METHYL ESTER, (E)- | 1.62 | 707 | C18H30O2 | 278 | 16714-85-5 | WileyRegistry8e |

| Compound Structure | Hit Spectrum |
|---|---|

PHENOL, 2-METHOXY-4-(2-PROPENYL)-
Formula C10H12O2, MW 164, CAS# 97-53-0, Entry# 46895
4-ALLYL-2-METHOXY-PHENOL

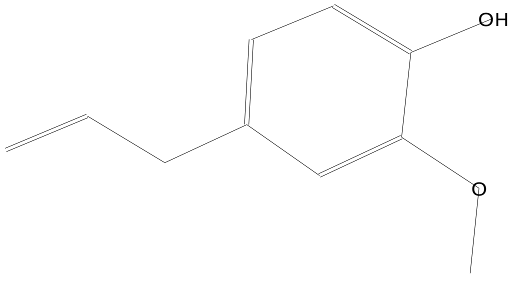


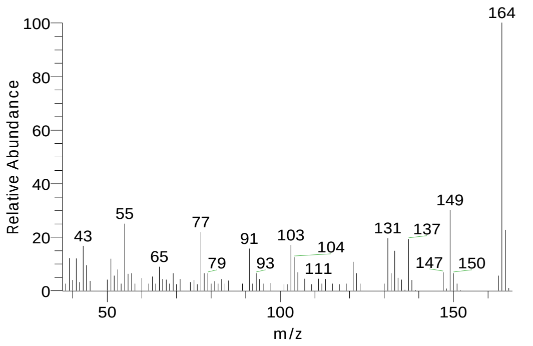


PHENOL, 2-METHOXY-4-(1-PROPENYL)-
Formula C10H12O2, MW 164, CAS# 97-54-1, Entry# 46882
2-METHOXY-4-[(1E)-1-PROPENYL]PHENOL #

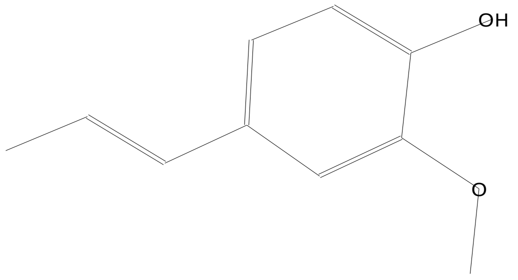


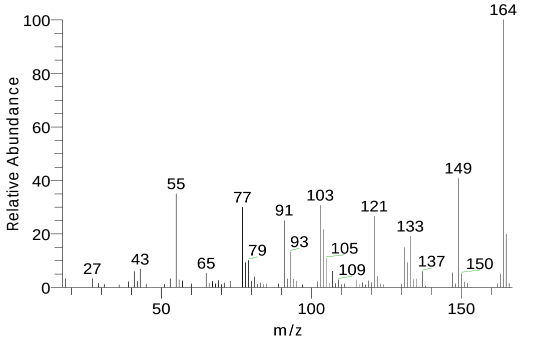

# My GC-MS Report

Compound Structure | Hit Spectrum

PHENOL, 2-METHOXY-5-(1-PROPENYL)-, (E)-
Formula C10H12O2, MW 164, CAS# 19784-98-6, Entry# 46987
2-METHOXY-5-[(1E)-1-PROPENYL]PHENOL #

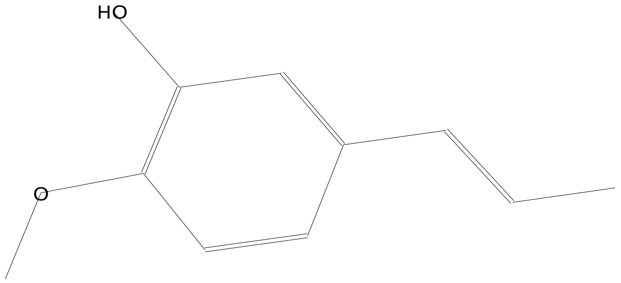


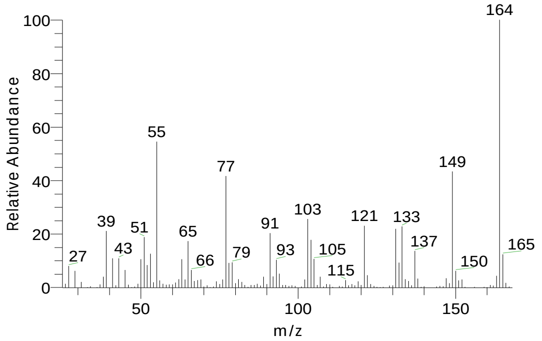


10-Heptadecen-8-ynoic acid, methyl ester, (E)-
Formula C18H30O2, MW 278, CAS# 16714-85-5, Entry# 48853
Methyl (10E)-10-heptadecen-8-ynoate #

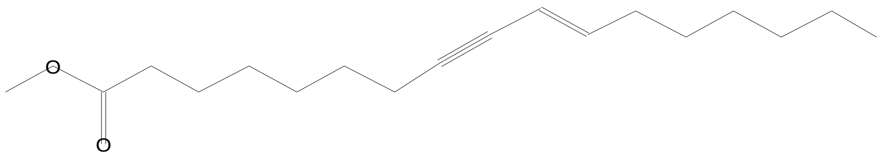


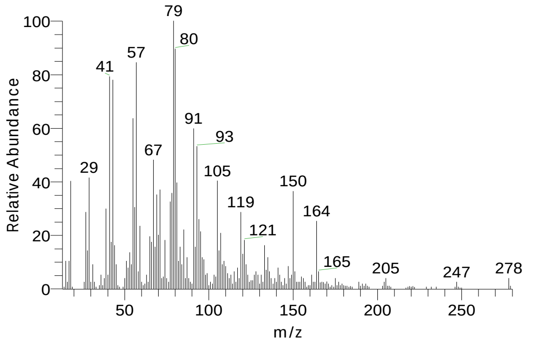


10-HEPTADECEN-8-YNOIC ACID, METHYL ESTER, (E)-
Formula C18H30O2, MW 278, CAS# 16714-85-5, Entry# 169022
METHYL (10E)-10-HEPTADECEN-8-YNOATE #

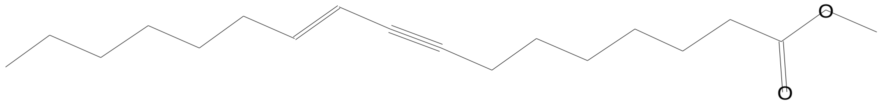


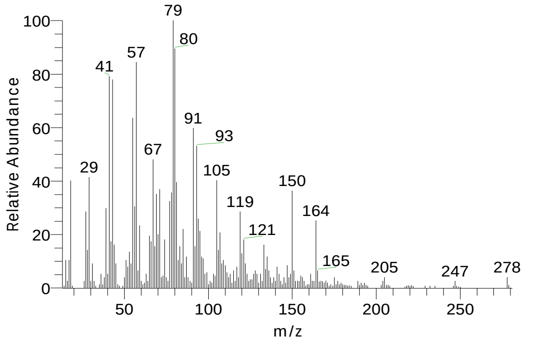


Clove_SayedMekky_DrSayed #4468 RT: 17.98 AV: 1 NL: 1.66E5
T: + c EI Full ms [50.000-500.000]

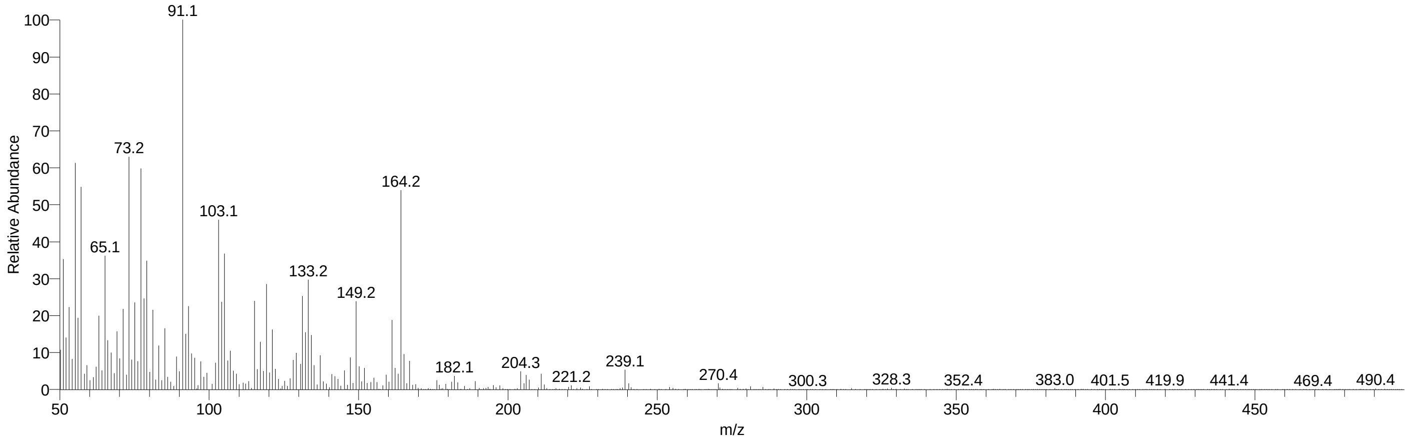


| RT | Compound Name | Area % | MF | Molecular Formula | Molecular Weight | Cas # | Library |
|---|---|---|---|---|---|---|---|
| 17.98 | Methyl 2,4-tridecadiynoate | 1.66 | 749 | C14H20O2 | 220 | NA | mainlib |
| 17.98 | Methyl 4,6-tetradecadiynoate | 1.66 | 755 | C15H22O2 | 234 | NA | mainlib |
| 17.98 | 10-Heptadecen-8-ynoic acid, methyl ester, (E)- | 1.66 | 714 | C18H30O2 | 278 | 16714-85-5 | mainlib |

# My GC-MS Report

| RT | Compound Name | Area % | MF | Molecular Formula | Molecular Weight | Cas # | Library |
|---|---|---|---|---|---|---|---|
| 17.98 | 10-HEPTADECEN-8-YNOIC ACID, METHYL ESTER, (E)- | 1.66 | 714 | C18H30O2 | 278 | 16714-85-5 | WileyRegistry8e |
| 17.98 | Panaxydol | 1.66 | 714 | C17H24O2 | 260 | 72800-72-7 | mainlib |

| Compound Structure | Hit Spectrum |
|---|---|

Methyl 2,4-tridecadiynoate
Formula C14H20O2, MW 220, CAS# NA, Entry# 59310
$:28AVAATZCMBIBGIK-UHFFFAOYSA-N

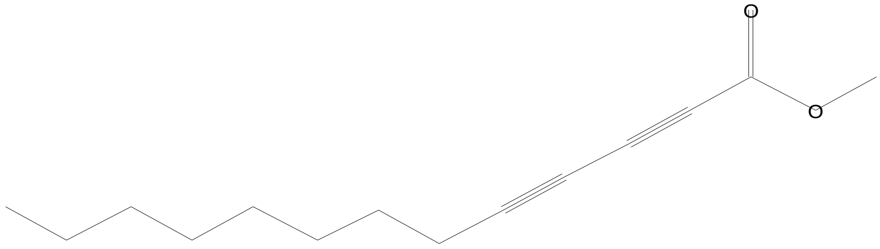


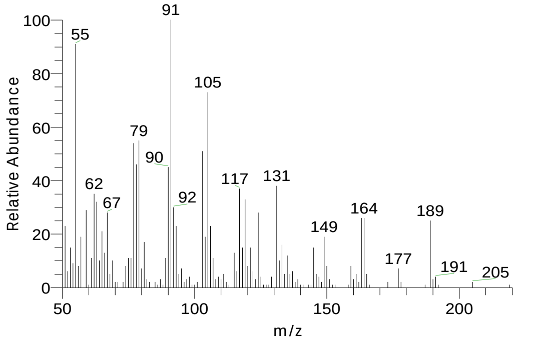


Methyl 4,6-tetradecadiynoate
Formula C15H22O2, MW 234, CAS# NA, Entry# 60961
$:28IYBFLSKYSDHDDR-UHFFFAOYSA-N

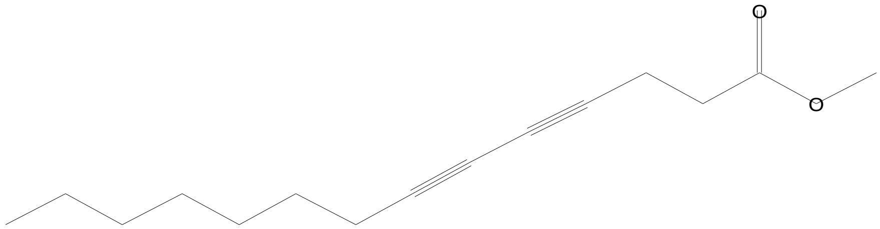


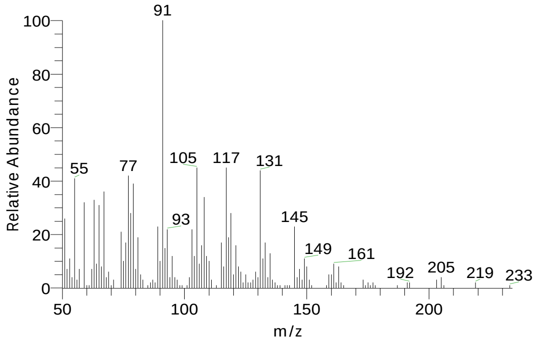


10-Heptadecen-8-ynoic acid, methyl ester, (E)-
Formula C18H30O2, MW 278, CAS# 16714-85-5, Entry# 48853
Methyl (10E)-10-heptadecen-8-ynoate #

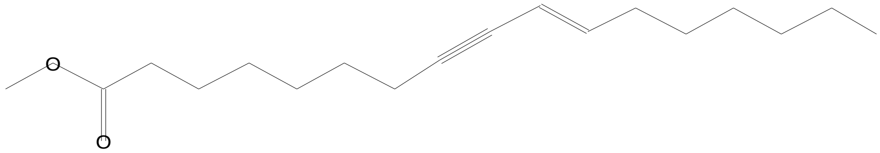


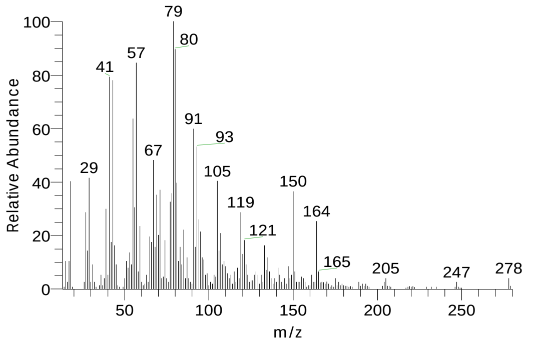


10-HEPTADECEN-8-YNOIC ACID, METHYL ESTER, (E)-
Formula C18H30O2, MW 278, CAS# 16714-85-5, Entry# 169022
METHYL (10E)-10-HEPTADECEN-8-YNOATE #

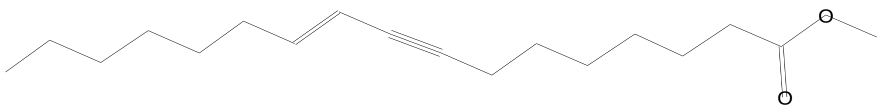


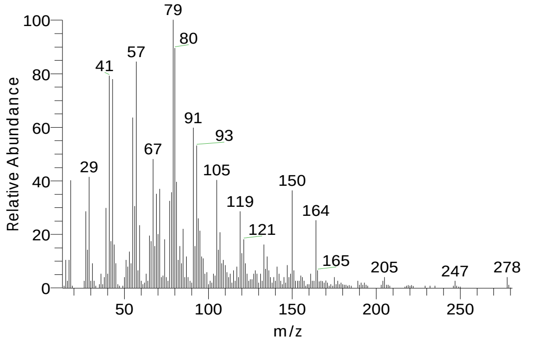

# My GC-MS Report

Compound Structure | Hit Spectrum

Panaxydol
Formula C17H24O2, MW 260, CAS# 72800-72-7, Entry# 102348
(R)-8-((2R,3S)-3-Heptyloxiran-2-yl)octa-1-en-4,6-diyn-3-ol

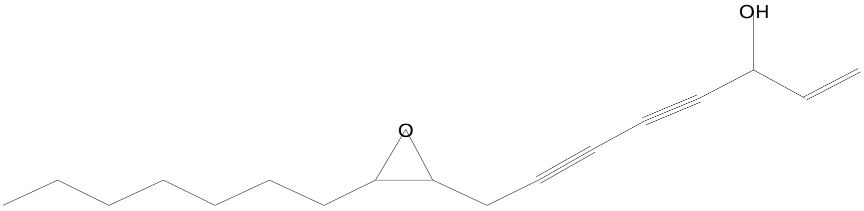


SI 693, RSI 714, mainlib, Entry# 102348, CAS# 72800-72-7, Panaxydol

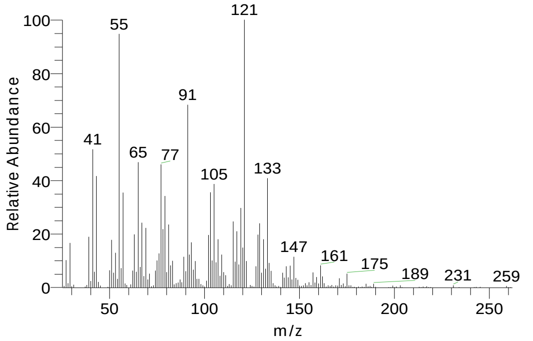


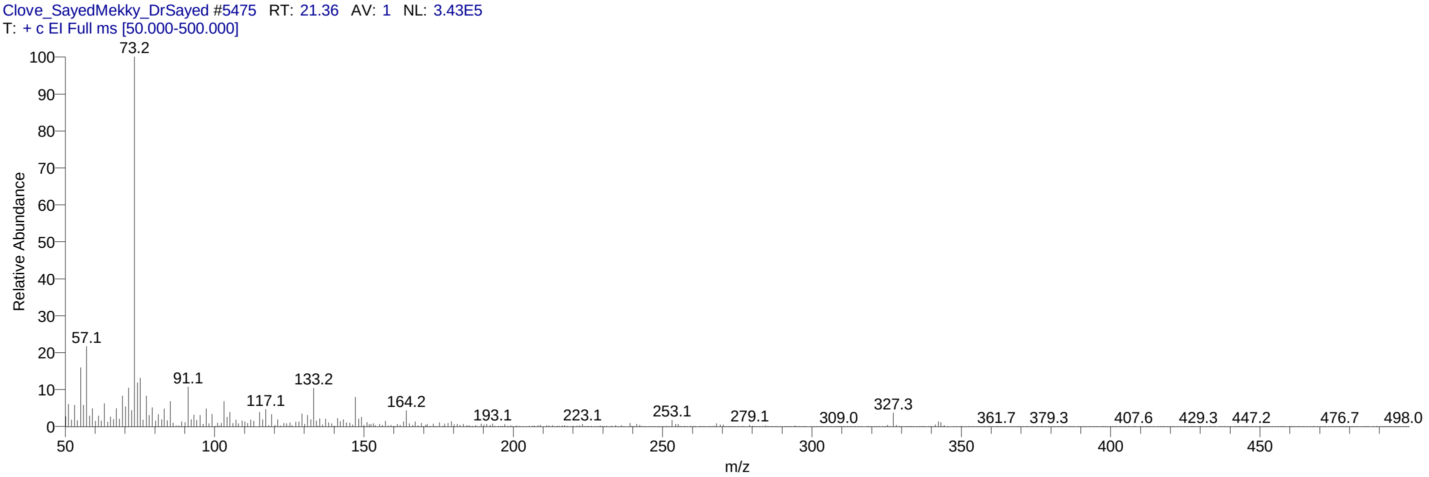


| RT | Compound Name | Area % | MF | Molecular Formula | Molecular Weight | Cas # | Library |
|---|---|---|---|---|---|---|---|
| 21.36 | Dodecanoic acid, 2,3-bis(acetyloxy)propyl ester | 1.48 | 726 | C19H34O6 | 358 | 55191-44-1 | mainlib |
| 21.36 | DODECANOIC ACID, 2,3-BIS(ACETYLOXY)PROPYL ESTER | 1.48 | 720 | C19H34O6 | 358 | 55191-44-1 | WileyRegistry8e |
| 21.36 | 2-Trimethylsiloxy-6-hexadecenoic acid, methyl ester | 1.48 | 691 | C20H40O3Si | 356 | NA | mainlib |
| 21.36 | t-Butyl-(2-[3-(2,2-dimethyl-6-methylene-cyclohexyl)-propyl]-[1,3]dithian-2-yl)-dimethyl-silane | 1.48 | 668 | C22H42S2Si | 398 | 95472-42-7 | mainlib |
| 21.36 | TERT-BUTYL-{2-[3-(2,2-DIMETHYL-6-METHYLENE-CYCLOHEXYL)-PROPYL]-[1,3]DITHIAN-2-YL}-DIMETHYL-SILANE | 1.48 | 668 | C22H42S2Si | 398 | NA | WileyRegistry8e |

Compound Structure | Hit Spectrum

Dodecanoic acid, 2,3-bis(acetyloxy)propyl ester
Formula C19H34O6, MW 358, CAS# 55191-44-1, Entry# 40755
2,3-Bis(acetyloxy)propyl laurate #

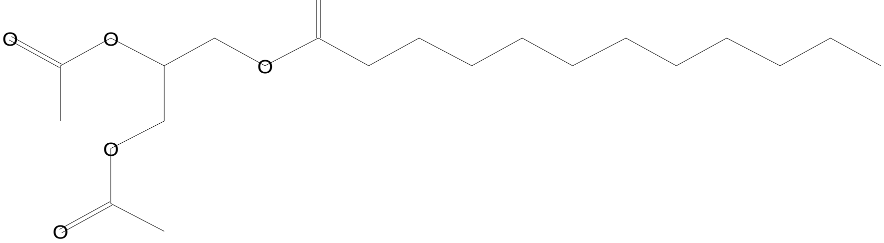


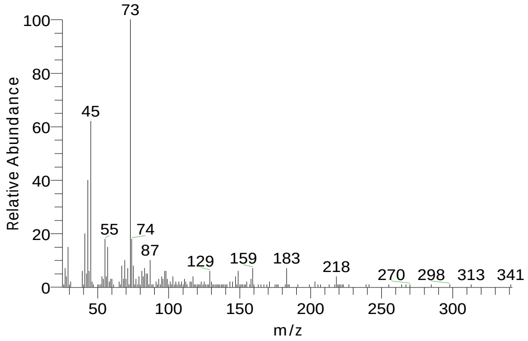

# *My GC-MS Report*

Compound Structure | Hit Spectrum

DODECANOIC ACID, 2,3-BIS(ACETYLOXY)PROPYL ESTER
Formula C19H34O6, MW 358, CAS# 55191-44-1, Entry# 233578
2,3-BIS(ACETYLOXY)PROPYL LAURATE #

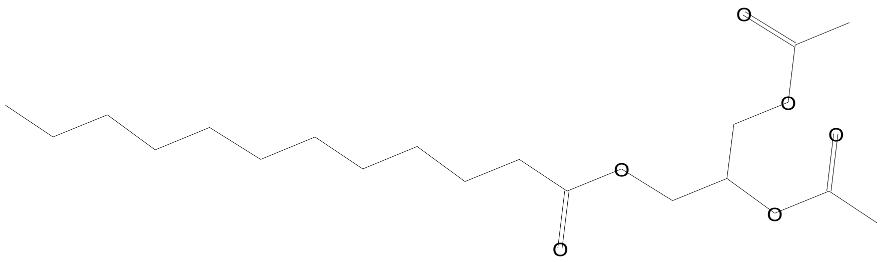


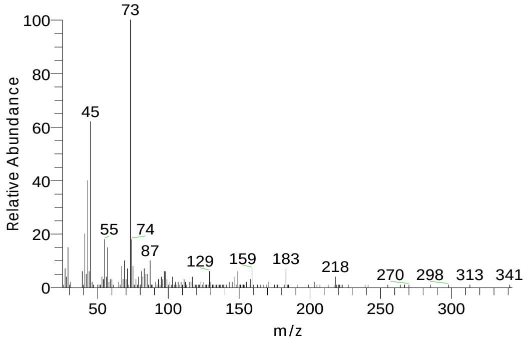


2-Trimethylsiloxy-6-hexadecenoic acid, methyl ester
Formula C20H40O3Si, MW 356, CAS# NA, Entry# 41294
$:28KICIJZNUWWRUSW-FOCLMDBBSA-N

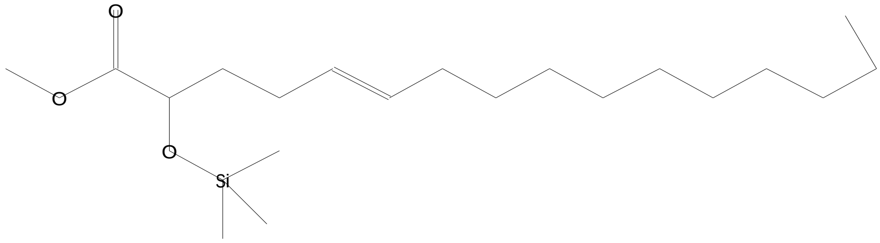


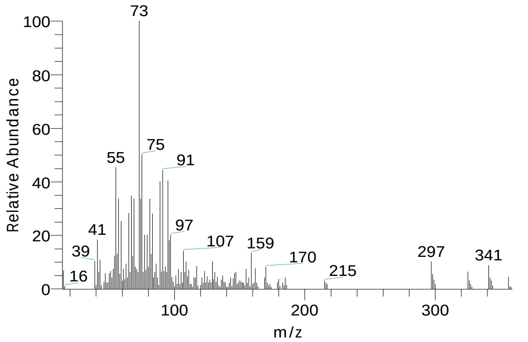


t-Butyl-(2-[3-(2,2-dimethyl-6-methylene-cyclohexyl)-propyl]-[1,3]dithian-2-yl)-dimethyl-silane
Formula C22H42S2Si, MW 398, CAS# 95472-42-7, Entry# 45252
tert-Butyl(2-[3-(2,2-dimethyl-6-methylenecyclohexyl)propyl]-1,3-dithian-2-yl)dimethylsilane #

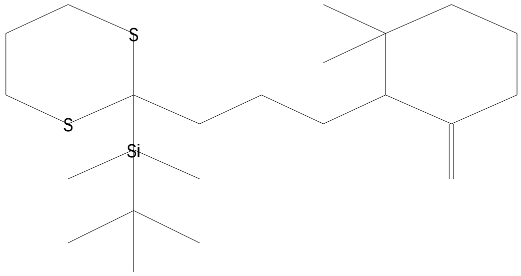


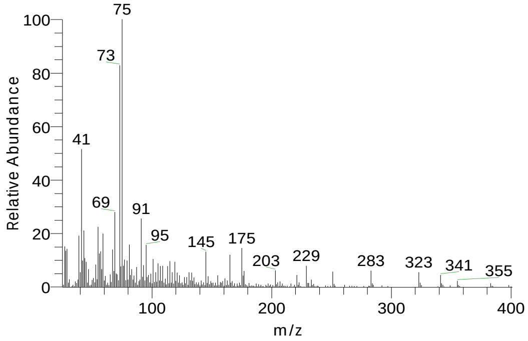


Formula C22H42S2Si, MW 398, CAS# NA, Entry# 369252

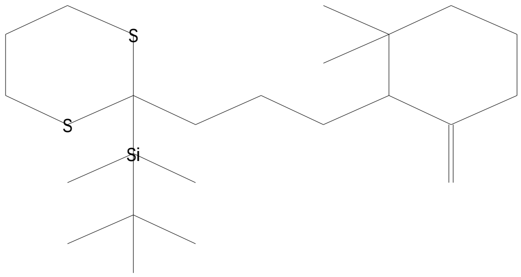


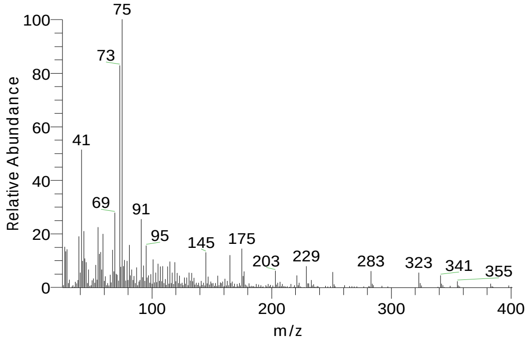

# My GC-MS Report

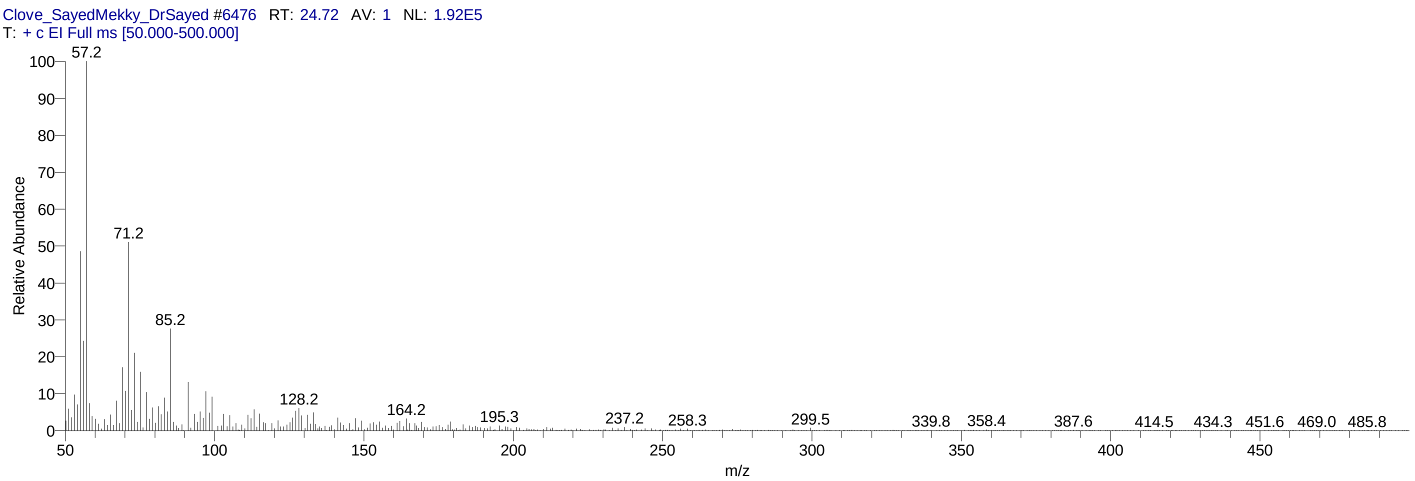


| RT | Compound Name | Area % | MF | Molecular Formula | Molecular Weight | Cas # | Library |
|---|---|---|---|---|---|---|---|
| 24.72 | DOTRIACONTANE | 1.10 | 723 | C32H66 | 450 | 544-85-4 | WileyRegistry8e |
| 24.72 | 1-CHLOROOCTADECANE | 1.10 | 774 | C18H37Cl | 288 | 3386-33-2 | WileyRegistry8e |
| 24.72 | 1,3,5-TRIAZINE-2,4-DIAMINE, 6-CHLORO-N-ETHYL- | 1.10 | 776 | C5H8ClN5 | 173 | 1007-28-9 | WileyRegistry8e |
| 24.72 | 1,25-Dihydroxyvitamin D3, TMS derivative | 1.10 | 705 | C30H52O3Si | 488 | 55759-94-9 | mainlib |
| 24.72 | 9,10-SECOCHOLESTA-5,7,10(19)-TRIENE-1,3-DIOL, 25-[(TRIMETHYLSILYL)OXY]-, (3á,5Z,7E)- | 1.10 | 705 | C30H52O3Si | 488 | 55759-94-9 | WileyRegistry8e |

Compound Structure | Hit Spectrum

DOTRIACONTANE
Formula C32H66, MW 450, CAS# 544-85-4, Entry# 274478
AI3-52367

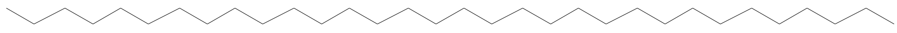

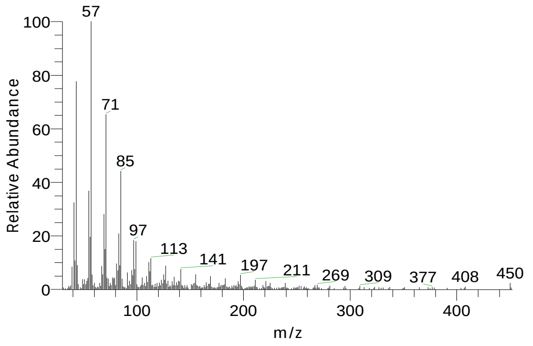


1-CHLOROOCTADECANE
Formula C18H37Cl, MW 288, CAS# 3386-33-2, Entry# 359171
1-CHLOROCTADECAN

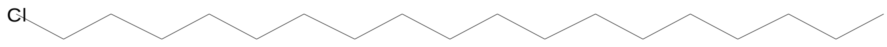


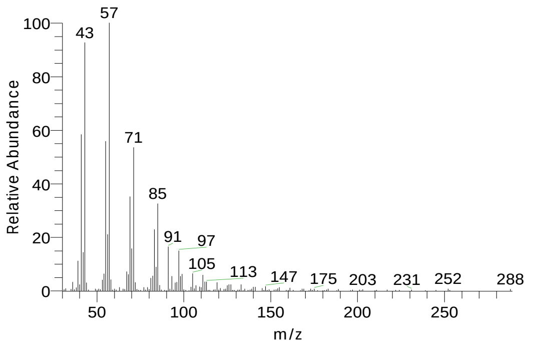

# *My GC-MS Report*

Compound Structure | Hit Spectrum

1,3,5-TRIAZINE-2,4-DIAMINE, 6-CHLORO-N-ETHYL-
Formula C5H8ClN5, MW 173, CAS# 1007-28-9, Entry# 56163
1,3, 5-TRIAZINE-2,4-DIAMINE, 6-CHLORO-N-ETHYL-

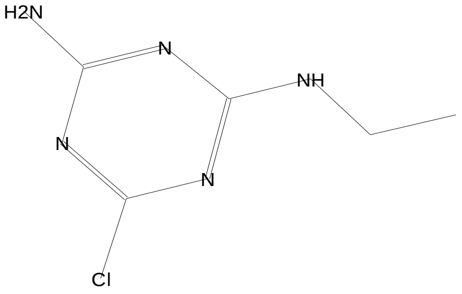


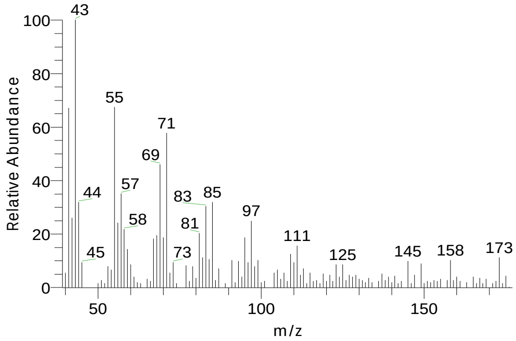


1,25-Dihydroxyvitamin D3, TMS derivative
Formula C30H52O3Si, MW 488, CAS# 55759-94-9, Entry# 19952
9,10-Secocholesta-5,7,10(19)-triene-1,3-diol, 25-[(trimethylsilyl)oxy]-, (3á,5Z,7E)-

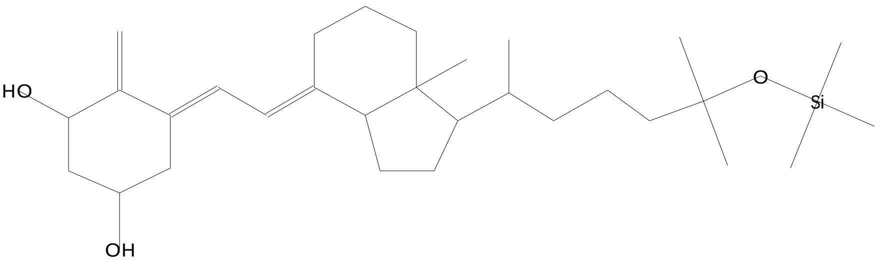


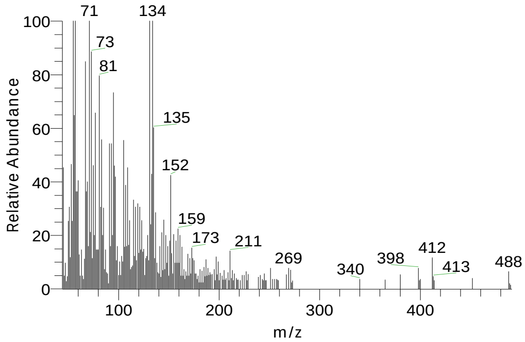


9,10-SECOCHOLESTA-5,7,10(19)-TRIENE-1,3-DIOL, 25-[(TRIMETHYLSILYL)OXY]-, (3á,5Z,7E)-
Formula C30H52O3Si, MW 488, CAS# 55759-94-9, Entry# 283552
(5E,7E)-25-[(TRIMETHYLSILYL)OXY]-9,10-SECOCHOLESTA-5,7,10-TRIENE-1,3-DIOL #

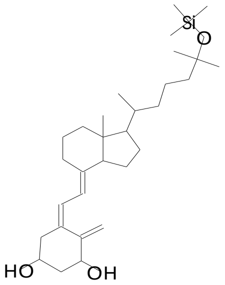


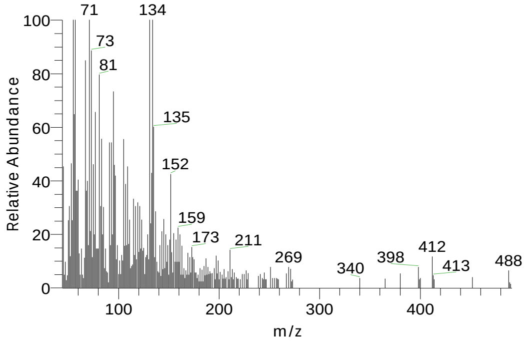


Clove_SayedMekky_DrSayed #7333 RT: 27.59 AV: 1 NL: 3.52E6
T: + c EI Full ms [50.000-500.000]

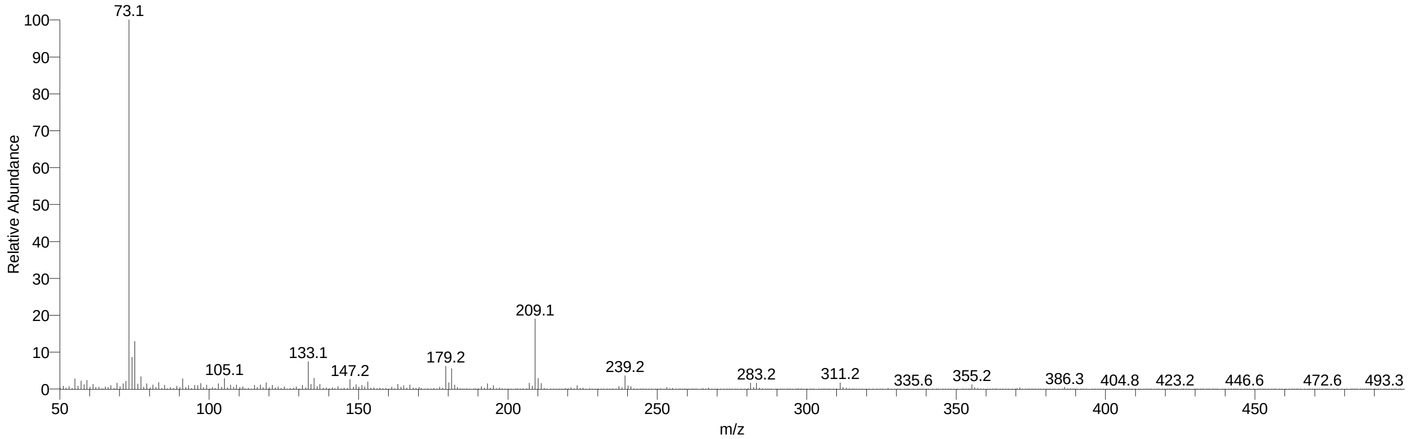


| RT | Compound Name | Area % | MF | Molecular Formula | Molecular Weight | Cas # | Library |
|---|---|---|---|---|---|---|---|
| 27.59 | (2-(3-METHOXY-4-[(TRIMETHYL SILYL)OXY]PHENYL)ETHOXY)(T RIMETHYL)SILANE # | 18.61 | 713 | C15H28O3Si2 | 312 | 4576-5 2-7 | WileyRegi stry8e |

# *My GC-MS Report*

| RT | Compound Name | Area % | MF | Molecular Formula | Molecular Weight | Cas # | Library |
|---|---|---|---|---|---|---|---|
| 27.59 | 1,2-BIS(TRIMETHYLSILYOXY)-4-TRIMETHYLSILYLOXYMETHYLBENZENE | 18.61 | 694 | C16H32O3Si3 | 356 | NA | WileyRegistry8e |
| 27.59 | (-)-Myrtenol, TBDMS derivative | 18.61 | 692 | C16H30OSi | 266 | NA | mainlib |
| 27.59 | 2-Methoxymandelic acid, ethyl ester, TMS | 18.61 | 754 | C14H22O4Si | 282 | NA | mainlib |
| 27.59 | 1,3,5-Cycloheptatriene, 7-methyl-7-phenyl-3-trimethylsilyl- | 18.61 | 683 | C17H22Si | 254 | NA | mainlib |

| Compound Structure | Hit Spectrum |
|---|---|

(2-(3-METHOXY-4-[(TRIMETHYLSILYL)OXY]PHENYL)ETHOXY)(TRIMETHYL)SILANE #
Formula C15H28O3Si2, MW 312, CAS# 4576-52-7, Entry# 199651
(2-(3-METHOXY-4-[(TRIMETHYLSILYL)OXY]PHENYL)ETHOXY)(TRIMETHYL)SILANE

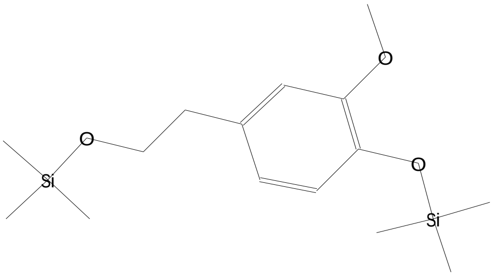


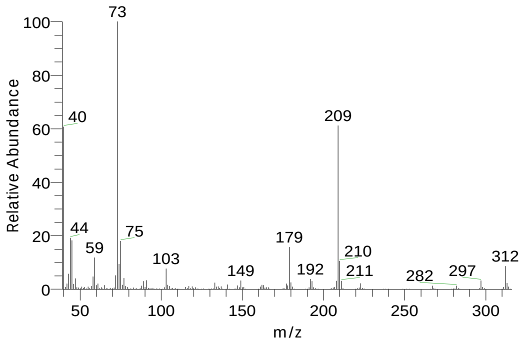


1,2-BIS(TRIMETHYLSILYOXY)-4-TRIMETHYLSILYLOXYMETHYLBENZENE
Formula C16H32O3Si3, MW 356, CAS# NA, Entry# 232178

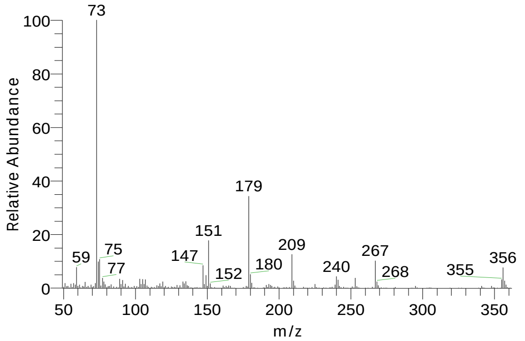


(-)-Myrtenol, TBDMS derivative
Formula C16H30OSi, MW 266, CAS# NA, Entry# 46004
(-)-Myrtenol, tert-butyldimethylsilyl ether

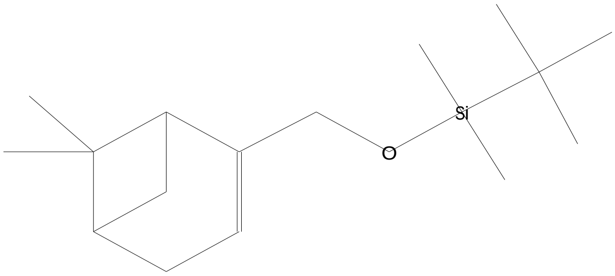


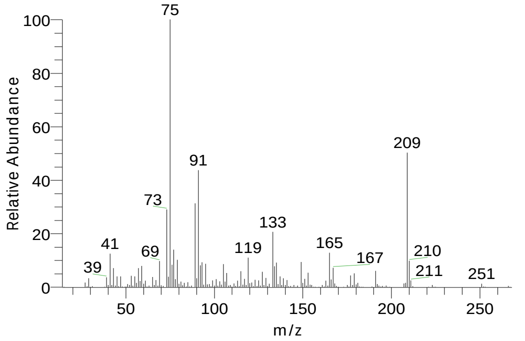


2-Methoxymandelic acid, ethyl ester, TMS
Formula C14H22O4Si, MW 282, CAS# NA, Entry# 189708
Ethyl (2-methoxyphenyl)[(trimethylsilyl)oxy]acetate #

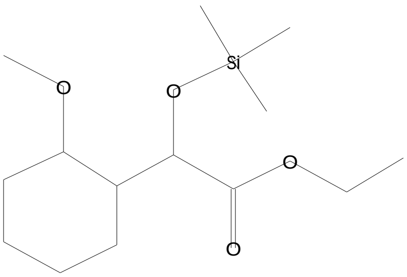


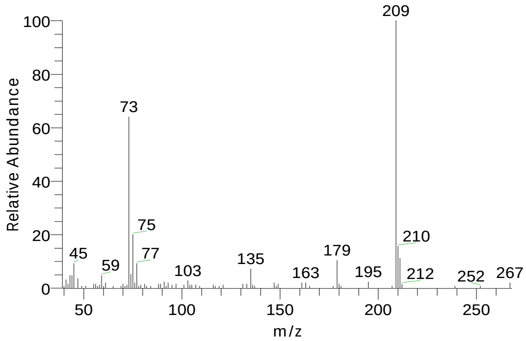

# My GC-MS Report

Compound Structure | Hit Spectrum

1,3,5-Cycloheptatriene, 7-methyl-7-phenyl-3-trimethylsilyl-
Formula C17H22Si, MW 254, CAS# NA, Entry# 40893
Trimethyl(5-methyl-5-phenyl-1,3,6-cycloheptatrien-1-yl)silane #

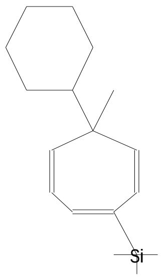


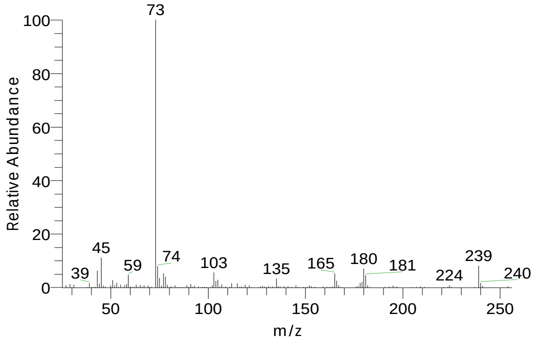


Clove_SayedMekky_DrSayed #7414 RT: 27.86 AV: 1 NL: 5.65E6
T: + c EI Full ms [50.000-500.000]

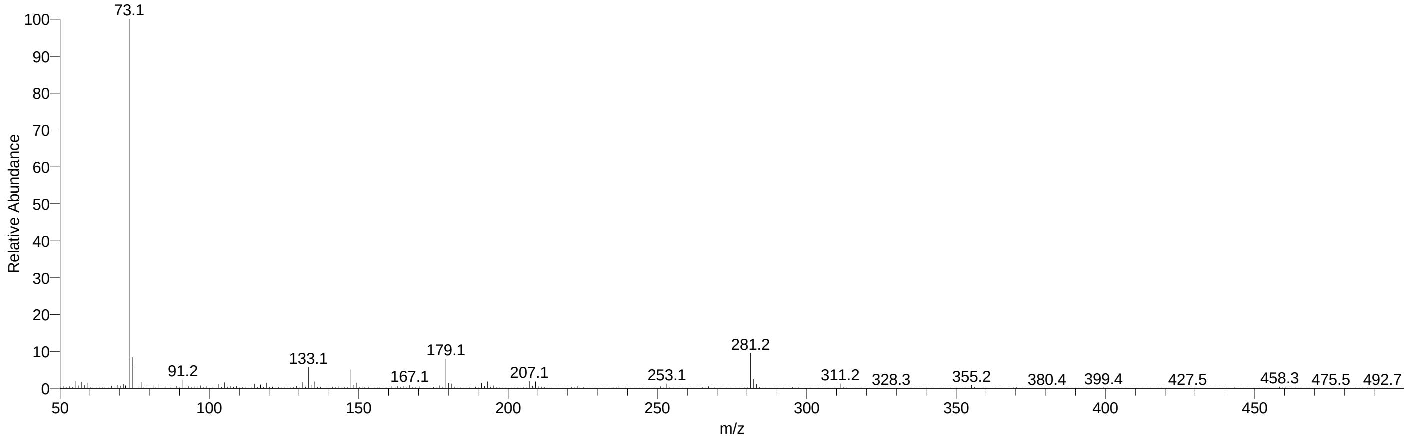


| RT | Compound Name | Area % | MF | Molecular Formula | Molecular Weight | Cas # | Library |
|---|---|---|---|---|---|---|---|
| 27.86 | Catechol, 2TBDMS derivative | 14.63 | 742 | C18H34O2Si2 | 338 | NA | mainlib |
| 27.86 | BENZENEACETIC ACID, 4-[(TRIMETHYLSILYL)OXY]-, TRIMETHYLSILYL ESTER | 14.63 | 727 | C14H24O3Si2 | 296 | 27750-57-8 | WileyRegistry8e |
| 27.86 | Pyrogallol, 3TBDMS derivative | 14.63 | 701 | C24H48O3Si3 | 468 | NA | mainlib |
| 27.86 | Benzoic acid, 4-methyl-2-trimethylsilyloxy-, trimethylsilyl ester | 14.63 | 673 | C14H24O3Si2 | 296 | 35-27-8 | mainlib |
| 27.86 | BENZENEACETIC ACID, 3-[(TRIMETHYLSILYL)OXY]-, TRIMETHYLSILYL ESTER | 14.63 | 673 | C14H24O3Si2 | 296 | 27750-55-6 | WileyRegistry8e |

Compound Structure | Hit Spectrum

Catechol, 2TBDMS derivative
Formula C18H34O2Si2, MW 338, CAS# NA, Entry# 42115
Pyrocatechol, bis(tert-butyldimethylsilyl) ether

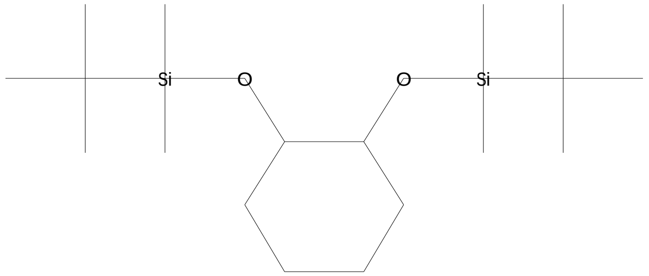


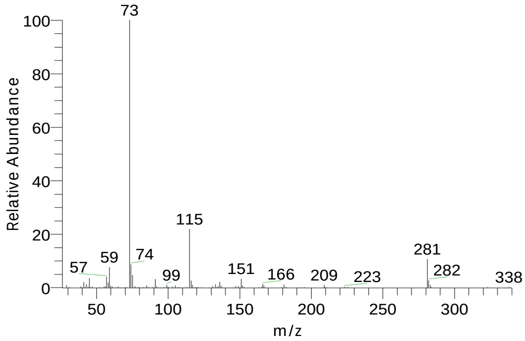

# My GC-MS Report

Compound Structure | Hit Spectrum

BENZENEACETIC ACID, 4-[(TRIMETHYLSILYL)OXY]-, TRIMETHYLSILYL ESTER
Formula C14H24O3Si2, MW 296, CAS# 27750-57-8, Entry# 185394
TRIMETHYLSILYL (4-[(TRIMETHYLSILYL)OXY]PHENYL)ACETATE #

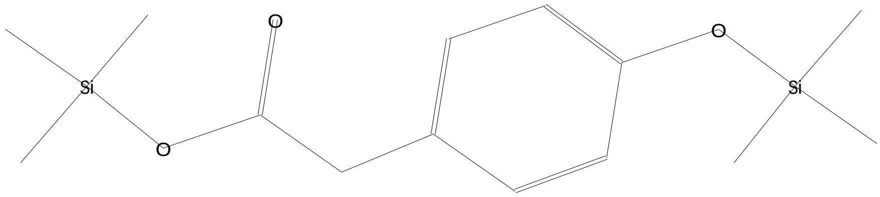


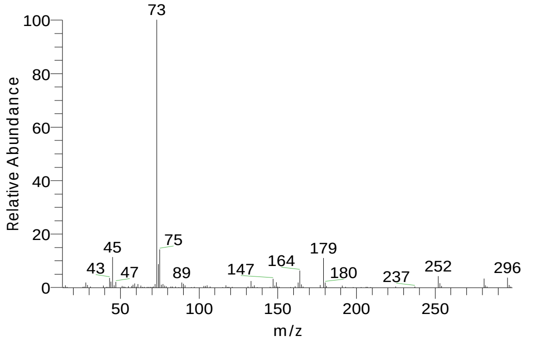


Pyrogallol, 3TBDMS derivative
Formula C24H48O3Si3, MW 468, CAS# NA, Entry# 43764
Benzene, 1,2,3-tris[(tert-butyldimethylsilyl)oxy]-

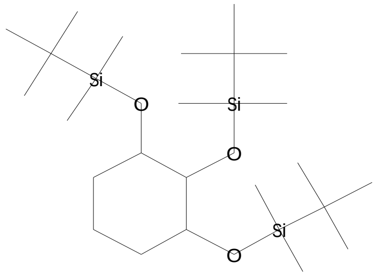


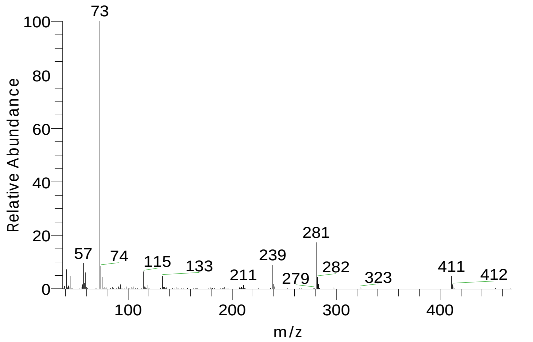


Benzoic acid, 4-methyl-2-trimethylsilyloxy-, trimethylsilyl ester
Formula C14H24O3Si2, MW 296, CAS# 35-27-8, Entry# 219921
$:28WMMUZRDZZSRQEF-UHFFFAOYSA-N

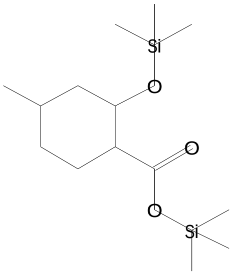


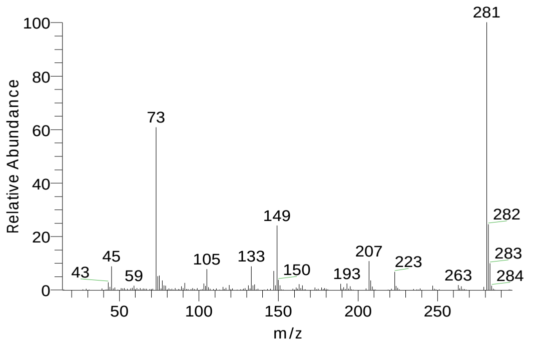


BENZENEACETIC ACID, 3-[(TRIMETHYLSILYL)OXY]-, TRIMETHYLSILYL ESTER
Formula C14H24O3Si2, MW 296, CAS# 27750-55-6, Entry# 185392
TRIMETHYLSILYL (3-[(TRIMETHYLSILYL)OXY]PHENYL)ACETATE #

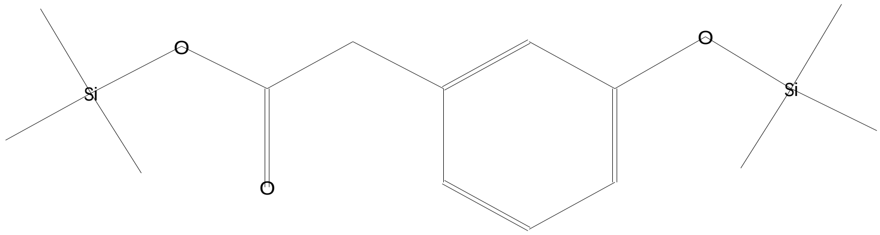


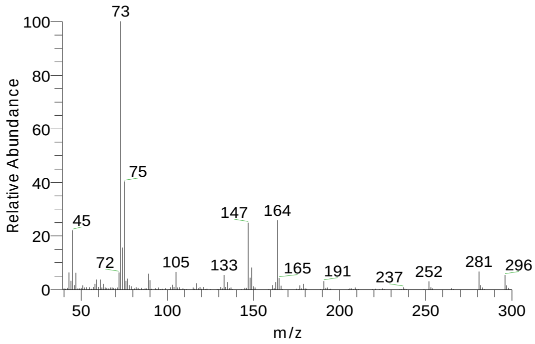

# My GC-MS Report

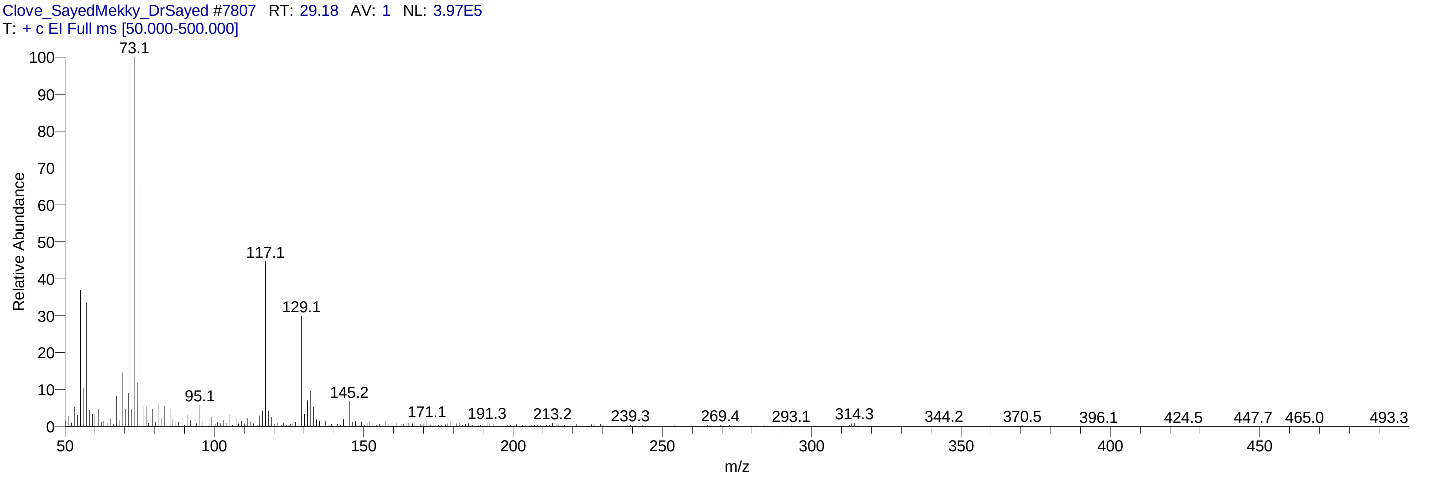


| RT | Compound Name | Area % | MF | Molecular Formula | Molecular Weight | Cas # | Library |
|---|---|---|---|---|---|---|---|
| 29.18 | 10-Undecynoic acid, TMS derivative | 3.02 | 750 | C14H26O2Si | 254 | NA | mainlib |
| 29.18 | HEXADECANOIC ACID, TRIMETHYLSILYL ESTER | 3.02 | 804 | C19H40O2Si | 328 | 55520-89-3 | WileyRegistry8e |
| 29.18 | 17-Octadecynoic acid, TMS derivative | 3.02 | 728 | C21H40O2Si | 352 | NA | mainlib |
| 29.18 | Palmitic Acid, TMS derivative | 3.02 | 726 | C19H40O2Si | 328 | 55520-89-3 | replib |
| 29.18 | HEXADECANOIC ACID, TRIMETHYLSILYL ESTER | 3.02 | 726 | C19H40O2Si | 328 | 55520-89-3 | WileyRegistry8e |

Compound Structure | Hit Spectrum

10-Undecynoic acid, TMS derivative
Formula C14H26O2Si, MW 254, CAS# NA, Entry# 45302
10-Undecynoic acid, trimethylsilyl ester

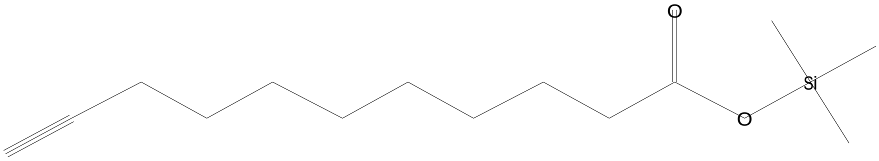


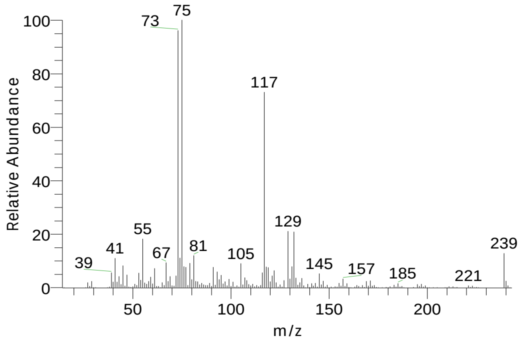


HEXADECANOIC ACID, TRIMETHYLSILYL ESTER
Formula C19H40O2Si, MW 328, CAS# 55520-89-3, Entry# 213029
TRIMETHYLSILYL PALMITATE #

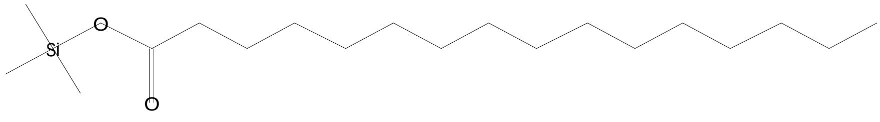


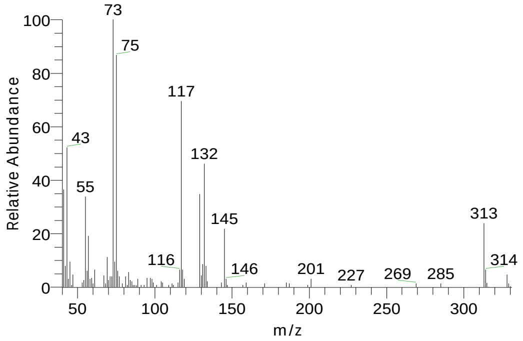

# *My GC-MS Report*

Compound Structure | Hit Spectrum

17-Octadecynoic acid, TMS derivative
Formula C21H40O2Si, MW 352, CAS# NA, Entry# 42157
17-Octadecynoic acid, trimethylsilyl ester

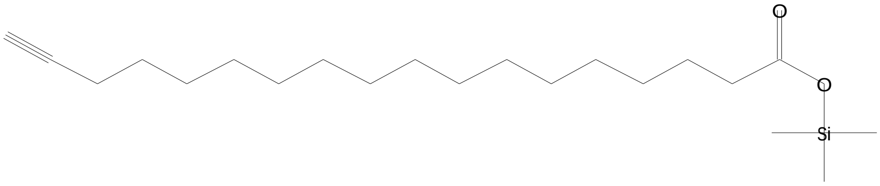


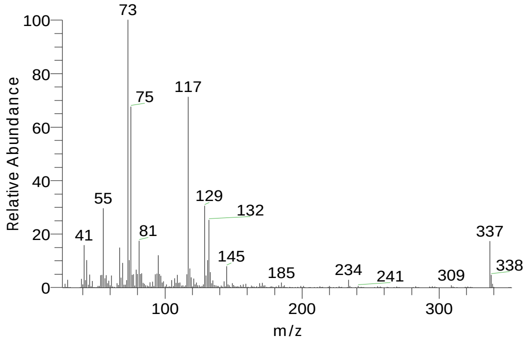


Palmitic Acid, TMS derivative
Formula C19H40O2Si, MW 328, CAS# 55520-89-3, Entry# 9711
Hexadecanoic acid, trimethylsilyl ester

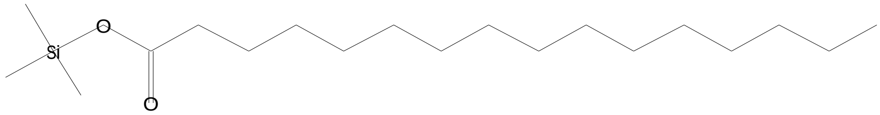


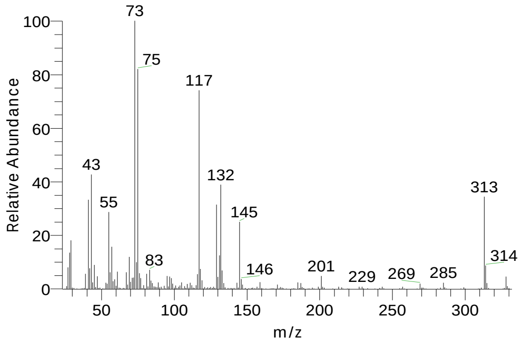


HEXADECANOIC ACID, TRIMETHYLSILYL ESTER
Formula C19H40O2Si, MW 328, CAS# 55520-89-3, Entry# 390490
TRIMETHYLSILYL PALMITATE #

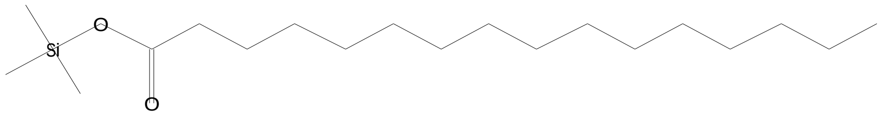


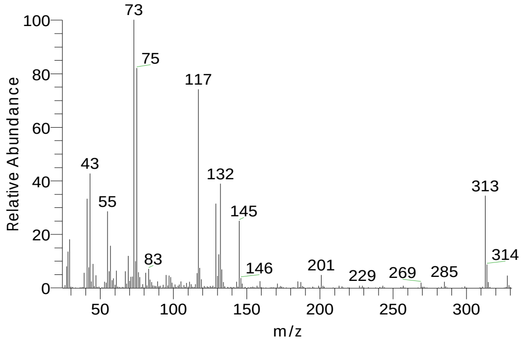


Clove_SayedMekky_DrSayed #8118 RT: 30.22 AV: 1 NL: 1.91E5
T: + c EI Full ms [50.000-500.000]

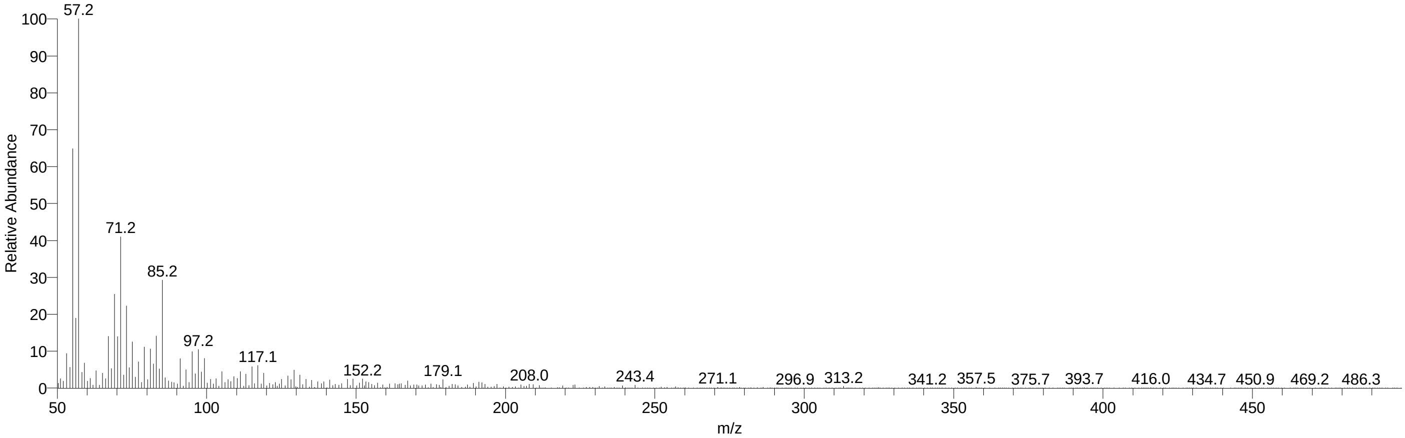

# *My GC-MS Report*

| RT | Compound Name | Area % | MF | Molecular Formula | Molecular Weight | Cas # | Library |
|---|---|---|---|---|---|---|---|
| 30.22 | PERMETHYLATED AND REDUCED PRODUCT OF DEGRADATION PRODUCT FROM H3-GLYCOLIPID BY L-L-FUCOSIDASE AND BY B-GALACTOSIDASE | 1.09 | 787 | C94H180N4O26 | 1780 | NA | WileyRegistry8e |
| 30.22 | 1,3,5-TRIAZINE-2,4-DIAMINE, 6-CHLORO-N-ETHYL- | 1.09 | 795 | C5H8ClN5 | 173 | 1007-28-9 | WileyRegistry8e |
| 30.22 | tert-Hexadecanethiol | 1.09 | 727 | C16H34S | 258 | 25360-09-2 | mainlib |
| 30.22 | 1-Hexadecanol, 2-methyl- | 1.09 | 723 | C17H36O | 256 | 2490-48-4 | mainlib |
| 30.22 | 1-HEXADECANOL, 2-METHYL- | 1.09 | 720 | C17H36O | 256 | 2490-48-4 | WileyRegistry8e |

| Compound Structure | Hit Spectrum |
|---|---|

Formula C94H180N4O26, MW 1780, CAS# NA, Entry# 305454

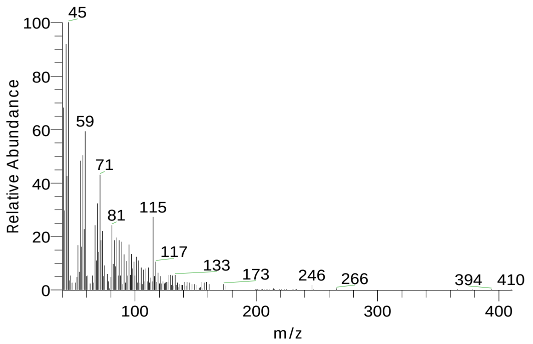


1,3,5-TRIAZINE-2,4-DIAMINE, 6-CHLORO-N-ETHYL-
Formula C5H8ClN5, MW 173, CAS# 1007-28-9, Entry# 56163
1,3, 5-TRIAZINE-2,4-DIAMINE, 6-CHLORO-N-ETHYL-

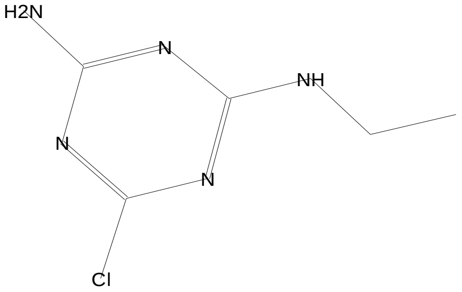


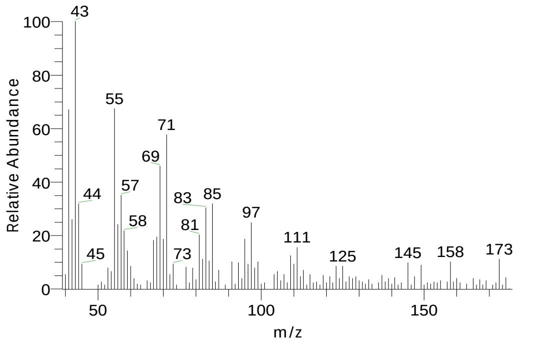


tert-Hexadecanethiol
Formula C16H34S, MW 258, CAS# 25360-09-2, Entry# 25117
1,1-Dimethyltetradecyl hydrosulfide #

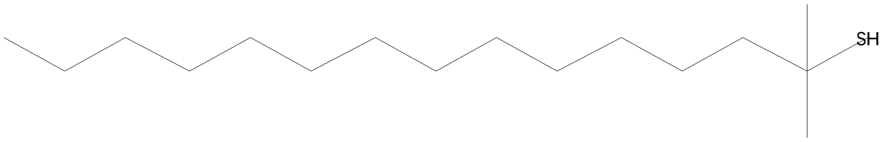


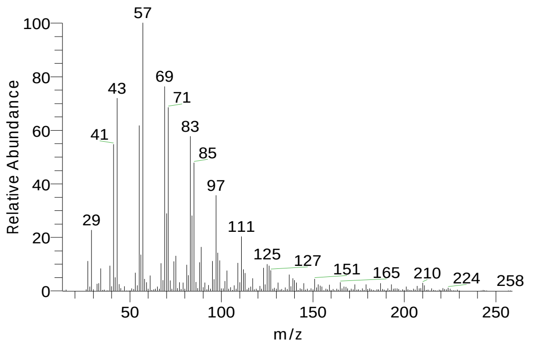

# My GC-MS Report

Compound Structure | Hit Spectrum

1-Hexadecanol, 2-methyl-
Formula C17H36O, MW 256, CAS# 2490-48-4, Entry# 24113
2-Methylhexadecan-1-ol

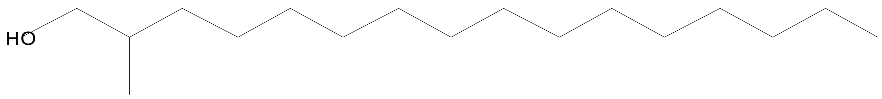


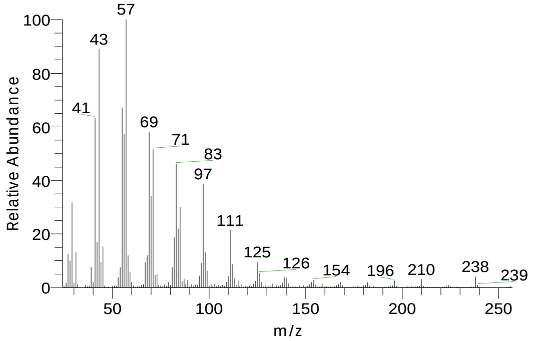


1-HEXADECANOL, 2-METHYL-
Formula C17H36O, MW 256, CAS# 2490-48-4, Entry# 146911
2-METHYLHEXADECAN-1-OL

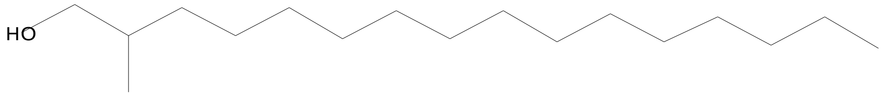


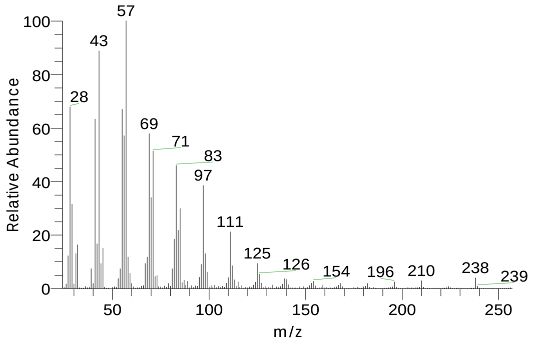


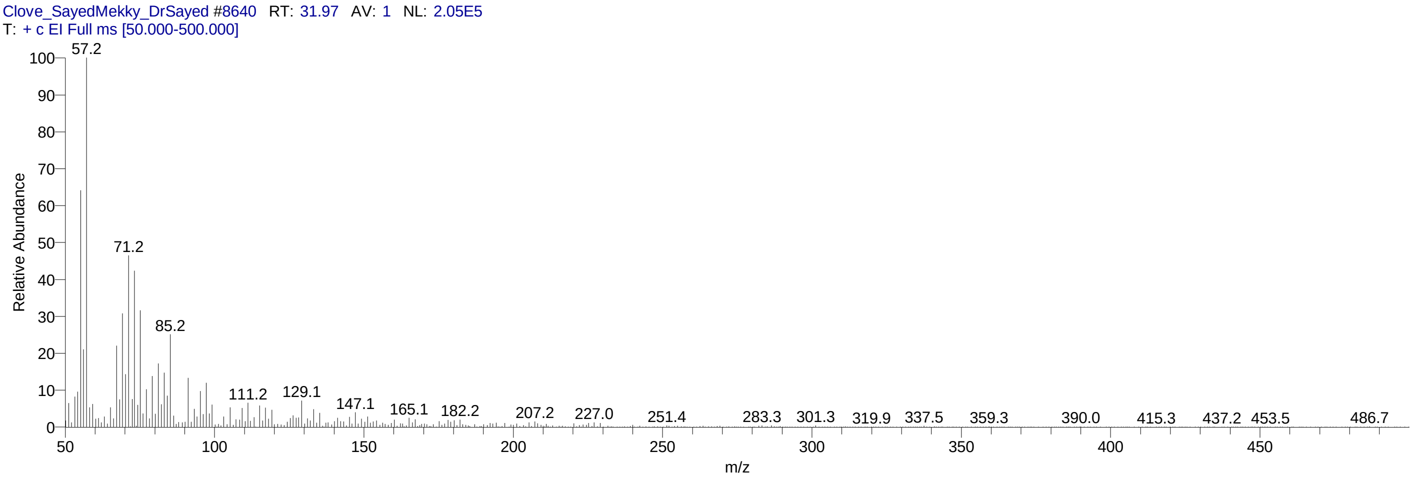


| RT | Compound Name | Area % | MF | Molecular Formula | Molecular Weight | Cas # | Library |
|---|---|---|---|---|---|---|---|
| 31.97 | 1,3,5-TRIAZINE-2,4-DIAMINE, 6-CHLORO-N-ETHYL- | 1.35 | 783 | C5H8ClN5 | 173 | 1007-28-9 | WileyRegistry8e |
| 31.97 | 2-Hexadecanol | 1.35 | 711 | C16H34O | 242 | 14852-31-4 | replib |
| 31.97 | 2-HEXADECANOL | 1.35 | 711 | C16H34O | 242 | 14852-31-4 | WileyRegistry8e |
| 31.97 | 1-CHLOROOCTADECANE | 1.35 | 758 | C18H37Cl | 288 | 3386-33-2 | WileyRegistry8e |
| 31.97 | 2,2,3,3,4,4 HEXADEUTERO OCTADECANAL | 1.35 | 694 | C18H30D6O | 274 | 56554-51-9 | WileyRegistry8e |

# *My GC-MS Report*

Compound Structure | Hit Spectrum

1,3,5-TRIAZINE-2,4-DIAMINE, 6-CHLORO-N-ETHYL-
Formula C5H8ClN5, MW 173, CAS# 1007-28-9, Entry# 56163
1,3, 5-TRIAZINE-2,4-DIAMINE, 6-CHLORO-N-ETHYL-

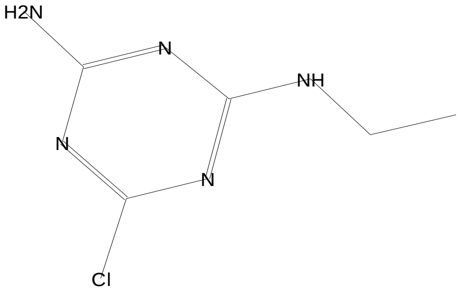


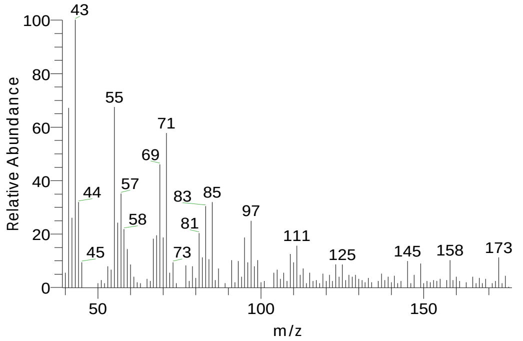


2-Hexadecanol
Formula C16H34O, MW 242, CAS# 14852-31-4, Entry# 4015
Hexadecanol-2

SI 701, RSI 711, replib, Entry# 4015, CAS# 14852-31-4, 2-Hexadecanol

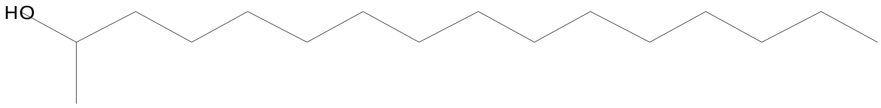


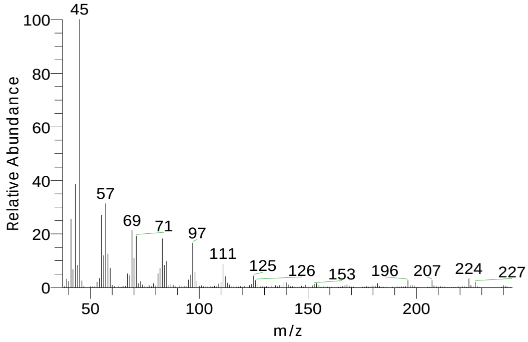


2-HEXADECANOL
Formula C16H34O, MW 242, CAS# 14852-31-4, Entry# 132208
HEXADECAN-2-OL

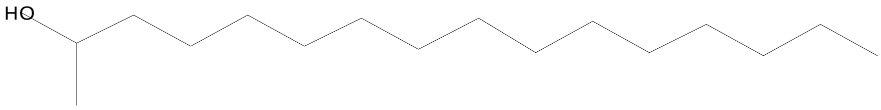


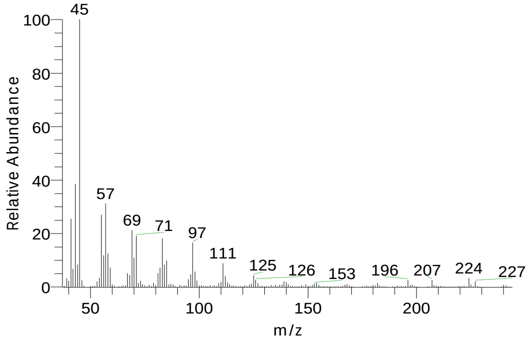


1-CHLOROOCTADECANE
Formula C18H37Cl, MW 288, CAS# 3386-33-2, Entry# 359171
1-CHLOROCTADECAN

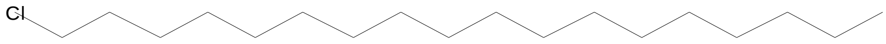


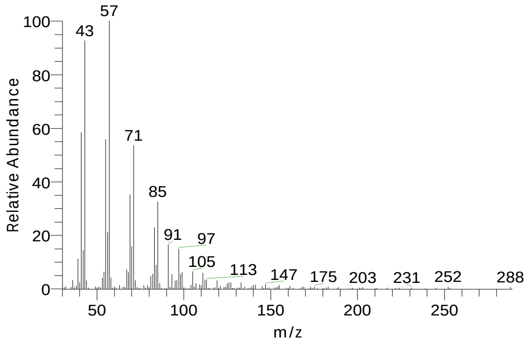

# My GC-MS Report

Compound Structure | Hit Spectrum

2,2,3,3,4,4 HEXADEUTERO OCTADECANAL
Formula C18H30D6O, MW 274, CAS# 56554-51-9, Entry# 159360

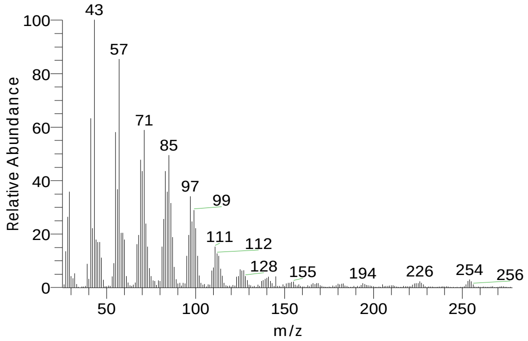


Clove_SayedMekky_DrSayed #8679 RT: 32.10 AV: 1 NL: 1.56E5
T: + c EI Full ms [50.000-500.000]

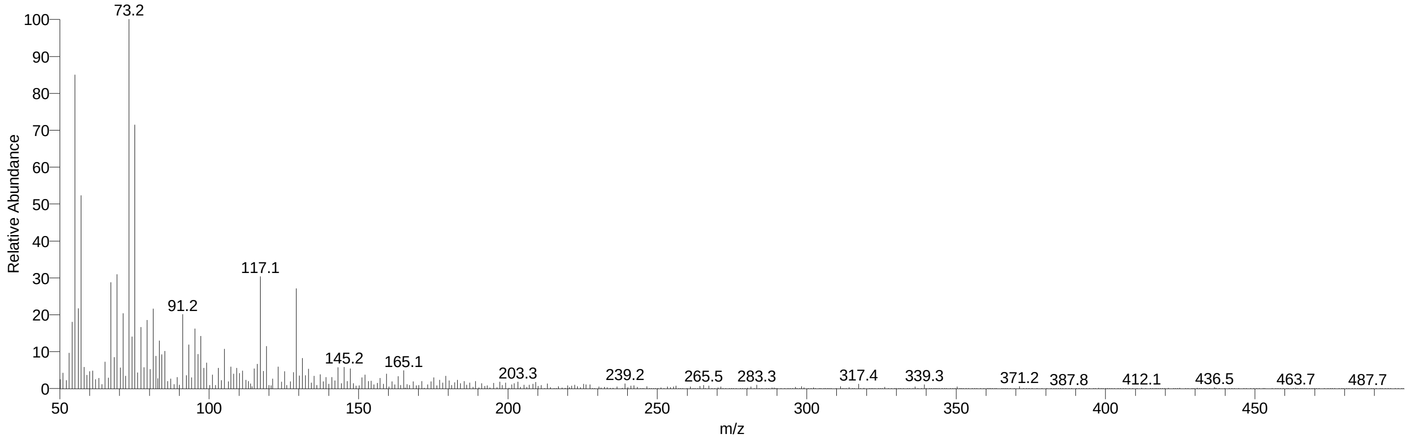


| RT | Compound Name | Area % | MF | Molecular Formula | Molecular Weight | Cas # | Library |
|---|---|---|---|---|---|---|---|
| 32.10 | 5,8,11-Eicosatrienoic acid, (Z)-, TMS derivative | 1.57 | 713 | C23H42O2Si | 378 | NA | mainlib |
| 32.10 | TRISTRIMETHYLSILYL ETHER DERIVATIVE OF 1,25-DIHYDROXYVITAMIN D2 | 1.57 | 696 | C37H68O3Si3 | 644 | NA | WileyRegistry8e |
| 32.10 | t-Butyl-(2-[3-(2,2-dimethyl-6-methylene-cyclohexyl)-propyl]-[1,3]dithian-2-yl)-dimethyl-silane | 1.57 | 698 | C22H42S2Si | 398 | 95472-42-7 | mainlib |
| 32.10 | TERT-BUTYL-{2-[3-(2,2-DIMETHYL-6-METHYLENE-CYCLOHEXYL)-PROPYL]-[1,3]DITHIAN-2-YL}-DIMETHYL-SILANE | 1.57 | 698 | C22H42S2Si | 398 | NA | WileyRegistry8e |
| 32.10 | à-D-MANNOPYRANOSIDE, METHYL, CYCLIC 2,3:4,6-BIS(BUTYLBORONATE) | 1.57 | 741 | C15H28B2O6 | 326 | 54400-84-9 | WileyRegistry8e |

Compound Structure | Hit Spectrum

5,8,11-Eicosatrienoic acid, (Z)-, TMS derivative
Formula C23H42O2Si, MW 378, CAS# NA, Entry# 41326
cis-5,8,11-Eicosatrienoic acid, trimethylsilyl ester

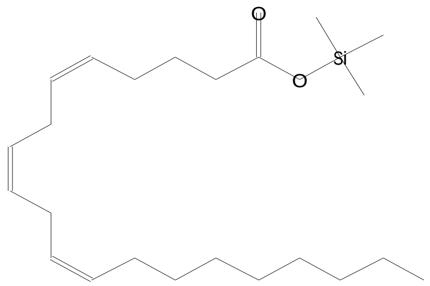


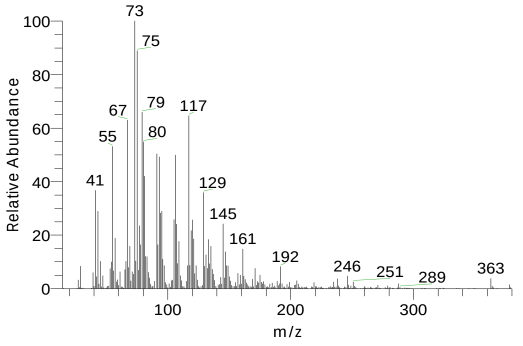

# My GC-MS Report

Compound Structure | Hit Spectrum

TRISTRIMETHYLSILYL ETHER DERIVATIVE OF 1,25-DIHYDROXYVITAMIN D2
Formula C37H68O3Si3, MW 644, CAS# NA, Entry# 299431

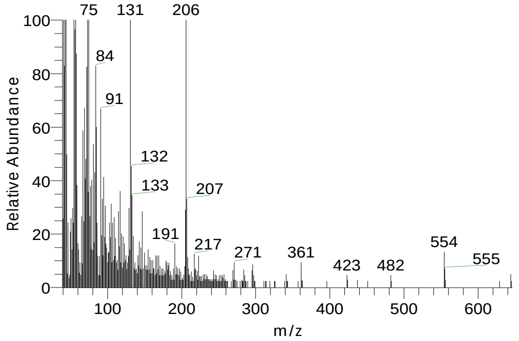


t-Butyl-(2-[3-(2,2-dimethyl-6-methylene-cyclohexyl)-propyl]-[1,3]dithian-2-yl)-dimethyl-silane
Formula C22H42S2Si, MW 398, CAS# 95472-42-7, Entry# 45252
tert-Butyl(2-[3-(2,2-dimethyl-6-methylenecyclohexyl)propyl]-1,3-dithian-2-yl)dimethylsilane #

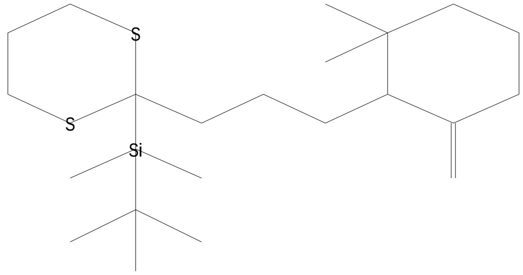


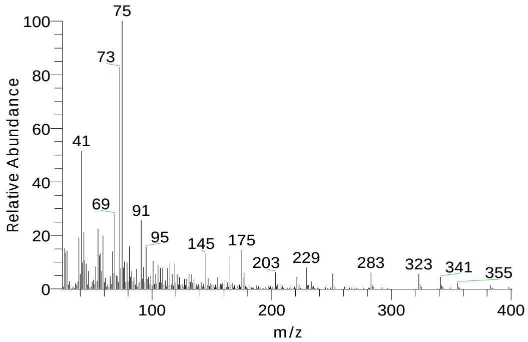


Formula C22H42S2Si, MW 398, CAS# NA, Entry# 369252

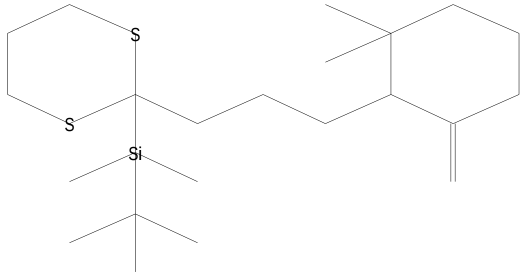


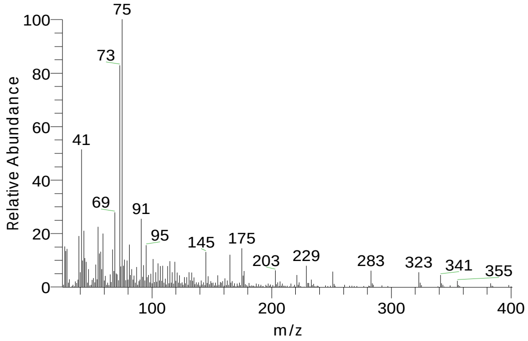


à-D-MANNOPYRANOSIDE, METHYL, CYCLIC 2,3:4,6-BIS(BUTYLBORONATE)
Formula C15H28B2O6, MW 326, CAS# 54400-84-9, Entry# 211125
A-MANNOPYRANOSIDE-1-METHYL-2,3-4,6-DI-BUTYLBORONATE

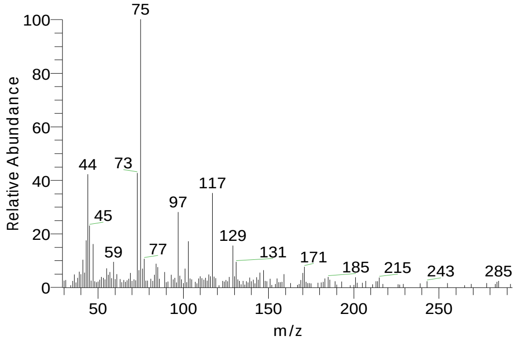

# *My GC-MS Report*

Clove_SayedMekky_DrSayed #9141 RT: 33.65 AV: 1 NL: 2.52E5
T: + c EI Full ms [50.000-500.000]

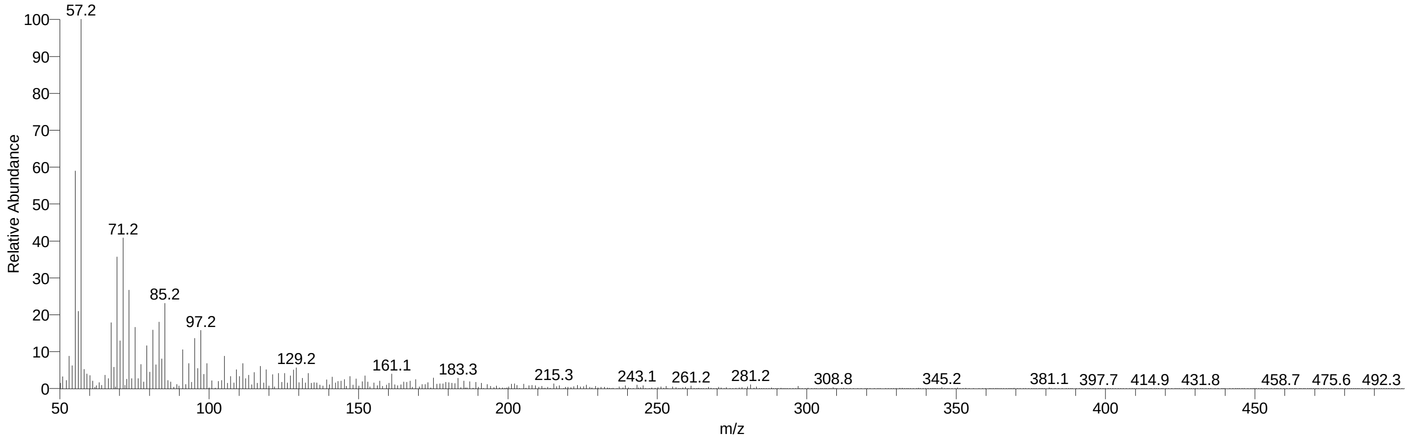


| RT | Compound Name | Area % | MF | Molecular Formula | Molecular Weight | Cas # | Library |
|---|---|---|---|---|---|---|---|
| 33.65 | 1,3,5-TRIAZINE-2,4-DIAMINE, 6-CHLORO-N-ETHYL- | 0.99 | 829 | C5H8ClN5 | 173 | 1007-28-9 | WileyRegistry8e |
| 33.65 | DOTRIACONTANE | 0.99 | 742 | C32H66 | 450 | 544-85-4 | WileyRegistry8e |
| 33.65 | ISOCHIAPIN B | 0.99 | 717 | C19H22O6 | 346 | NA | WileyRegistry8e |
| 33.65 | ISOCHIAPIN B %2< | 0.99 | 717 | C19H26O6 | 350 | NA | WileyRegistry8e |
| 33.65 | 2,2,3,3,4,4 HEXADEUTERO OCTADECANAL | 0.99 | 720 | C18H30D6O | 274 | 56554-51-9 | WileyRegistry8e |

Compound Structure

Hit Spectrum

1,3,5-TRIAZINE-2,4-DIAMINE, 6-CHLORO-N-ETHYL-
Formula C5H8ClN5, MW 173, CAS# 1007-28-9, Entry# 56163
1,3, 5-TRIAZINE-2,4-DIAMINE, 6-CHLORO-N-ETHYL-

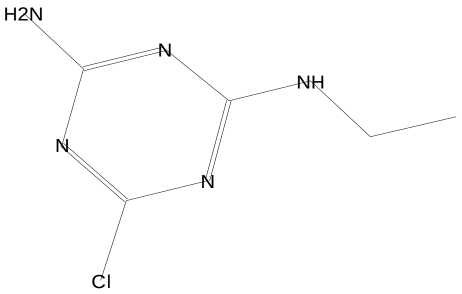


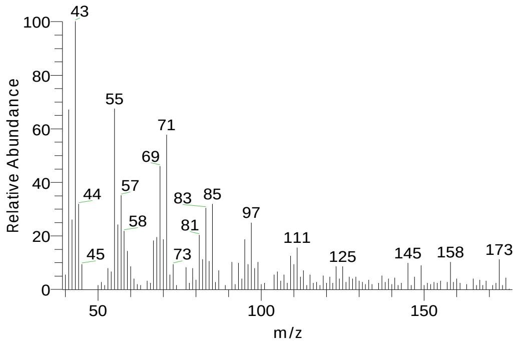


DOTRIACONTANE
Formula C32H66, MW 450, CAS# 544-85-4, Entry# 274478
AI3-52367

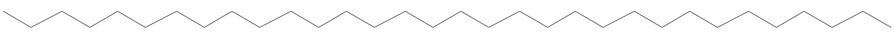

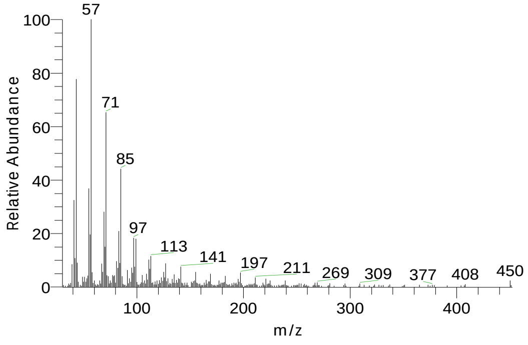

# My GC-MS Report

Compound Structure | Hit Spectrum

ISOCHIAPIN B
Formula C19H22O6, MW 346, CAS# NA, Entry# 225807

SI 716, RSI 717, WileyRegistry8e, Entry# 225807, CAS# NA, ISOCHIAPIN B

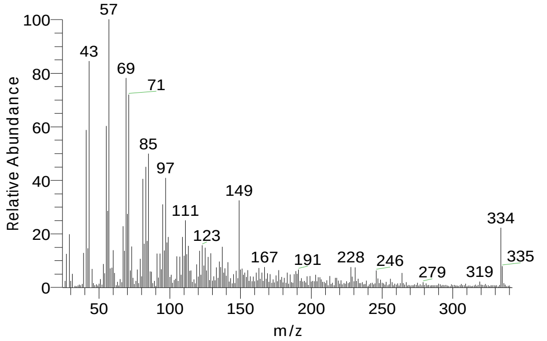


ISOCHIAPIN B %2<
Formula C19H26O6, MW 350, CAS# NA, Entry# 228500

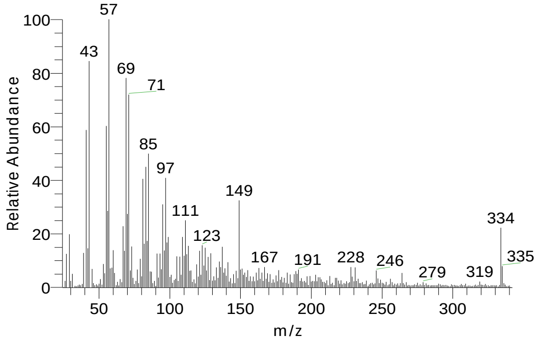


2,2,3,3,4,4 HEXADEUTERO OCTADECANAL
Formula C18H30D6O, MW 274, CAS# 56554-51-9, Entry# 159360

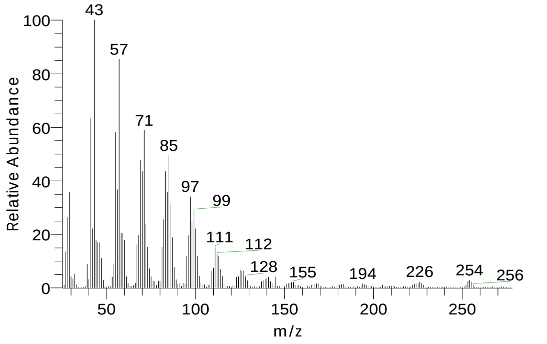


Clove_SayedMekky_DrSayed #10086 RT: 36.82 AV: 1 NL: 2.89E5
T: + c EI Full ms [50.000-500.000]

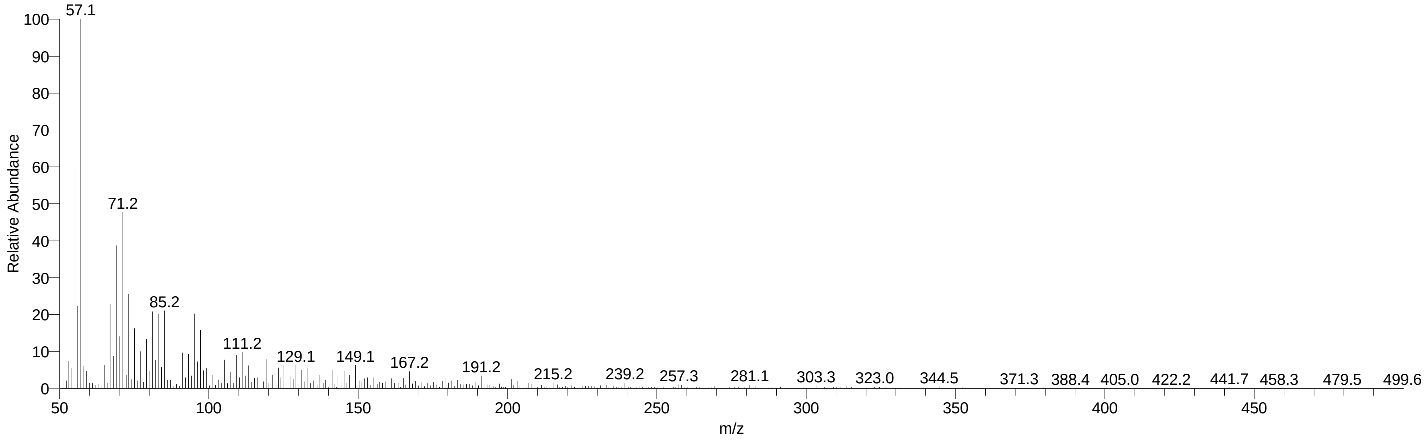


| RT | Compound Name | Area % | MF | Molecular Formula | Molecular Weight | Cas # | Library |
|---|---|---|---|---|---|---|---|
| 36.82 | ISOCHIAPIN B | 1.09 | 762 | C19H22O6 | 346 | NA | WileyRegistry8e |
| 36.82 | ISOCHIAPIN B %2< | 1.09 | 762 | C19H26O6 | 350 | NA | WileyRegistry8e |

# *My GC-MS Report*

| RT | Compound Name | Area % | MF | Molecular Formula | Molecular Weight | Cas # | Library |
|---|---|---|---|---|---|---|---|
| 36.82 | DOTRIACONTANE | 1.09 | 761 | C32H66 | 450 | 544-85-4 | WileyRegistry8e |
| 36.82 | 1,3,5-TRIAZINE-2,4-DIAMINE, 6-CHLORO-N-ETHYL- | 1.09 | 841 | C5H8ClN5 | 173 | 1007-28-9 | WileyRegistry8e |
| 36.82 | 1,25-Dihydroxyvitamin D3, TMS derivative | 1.09 | 757 | C30H52O3Si | 488 | 55759-94-9 | mainlib |

Compound Structure | Hit Spectrum

ISOCHIAPIN B
Formula C19H22O6, MW 346, CAS# NA, Entry# 225807

SI 761, RSI 762, WileyRegistry8e, Entry# 225807, CAS# NA, ISOCHIAPIN B

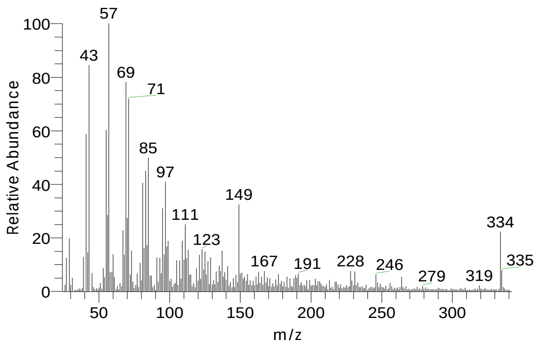


ISOCHIAPIN B %2<
Formula C19H26O6, MW 350, CAS# NA, Entry# 228500

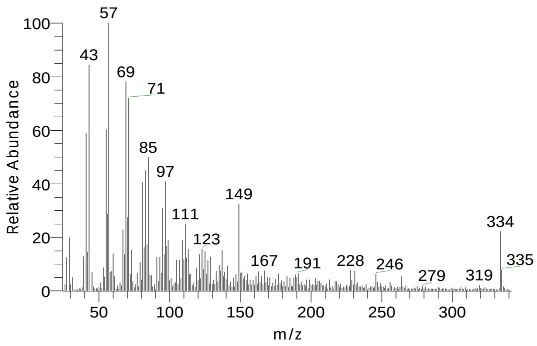


DOTRIACONTANE
Formula C32H66, MW 450, CAS# 544-85-4, Entry# 274478
AI3-52367

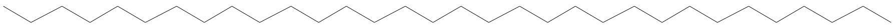

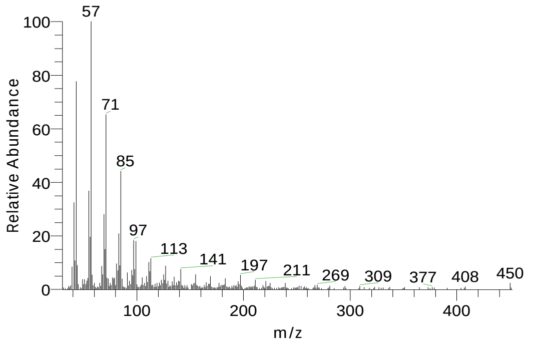


1,3,5-TRIAZINE-2,4-DIAMINE, 6-CHLORO-N-ETHYL-
Formula C5H8ClN5, MW 173, CAS# 1007-28-9, Entry# 56163
1,3, 5-TRIAZINE-2,4-DIAMINE, 6-CHLORO-N-ETHYL-

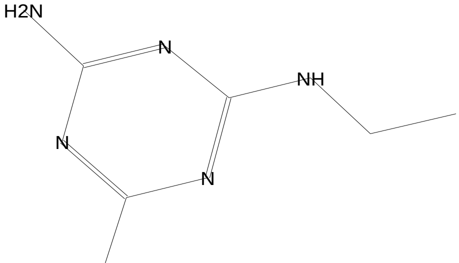


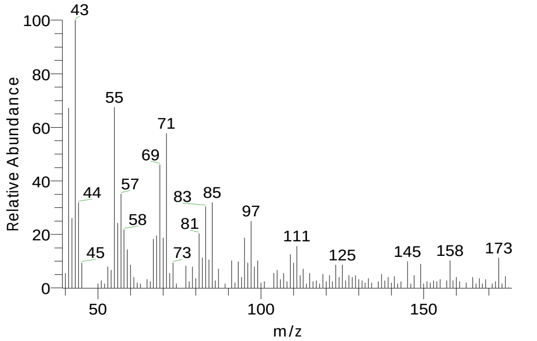

# *My GC-MS Report*

Compound Structure | Hit Spectrum

1,25-Dihydroxyvitamin D3, TMS derivative
Formula C30H52O3Si, MW 488, CAS# 55759-94-9, Entry# 19952
9,10-Secocholesta-5,7,10(19)-triene-1,3-diol, 25-[(trimethylsilyl)oxy]-, (3á,5Z,7E)-

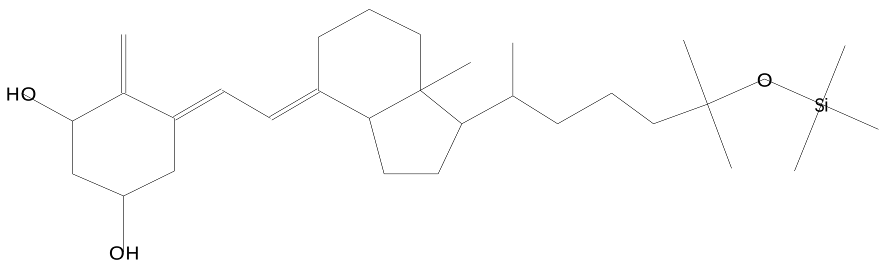


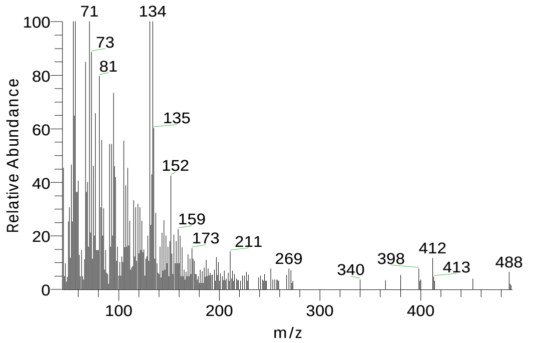


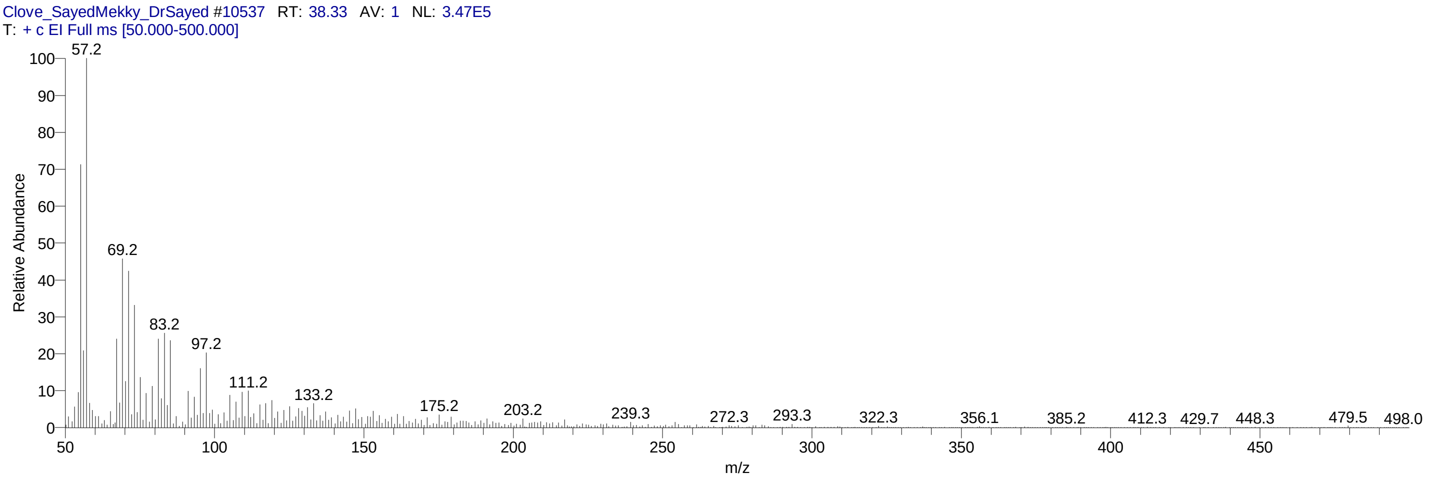


| RT | Compound Name | Area % | MF | Molecular Formula | Molecular Weight | Cas # | Library |
|---|---|---|---|---|---|---|---|
| 38.33 | 2-ACETYL-3-(2-CINNAMIDO)ETHYL-7-METHOXYINDOLE | 1.21 | 761 | C22H22N2O3 | 362 | NA | WileyRegistry8e |
| 38.33 | 4H-1-BENZOPYRAN-4-ONE, 2-(3,4-DIMETHOXYPHENYL)-3,5-DIHYDROXY-7-METHOXY- | 1.21 | 732 | C18H16O7 | 344 | 6068-80-0 | WileyRegistry8e |
| 38.33 | tert-Hexadecanethiol | 1.21 | 749 | C16H34S | 258 | 25360-09-2 | mainlib |
| 38.33 | 2,2-DIDEUTERO OCTADECANAL | 1.21 | 732 | C18H34D2O | 270 | 56555-07-8 | WileyRegistry8e |
| 38.33 | IRON IODIDE COMPLEX I | 1.21 | 720 | C26H26FeIN4O4 | 641 | NA | WileyRegistry8e |

Compound Structure | Hit Spectrum

2-ACETYL-3-(2-CINNAMIDO)ETHYL-7-METHOXYINDOLE
Formula C22H22N2O3, MW 362, CAS# NA, Entry# 236392

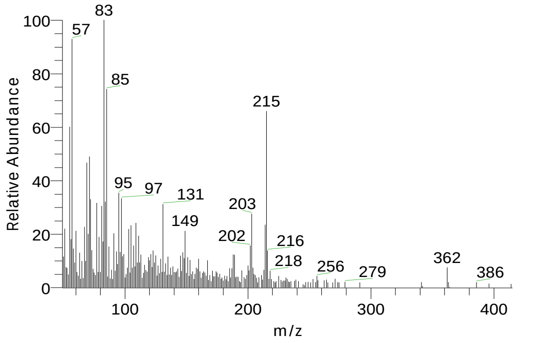

# *My GC-MS Report*

Compound Structure | Hit Spectrum

4H-1-BENZOPYRAN-4-ONE, 2-(3,4-DIMETHOXYPHENYL)-3,5-DIHYDROXY-7-METHOXY-
Formula C18H16O7, MW 344, CAS# 6068-80-0, Entry# 224392
3',4',7-TRIMETHYLQUERCETIN

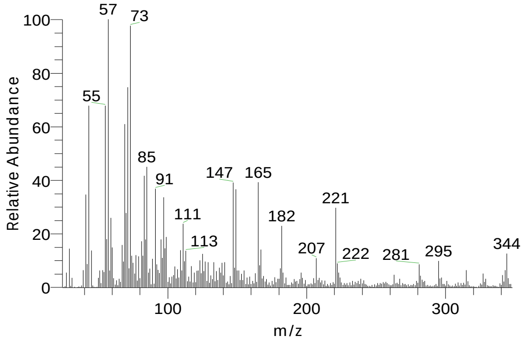


tert-Hexadecanethiol
Formula C16H34S, MW 258, CAS# 25360-09-2, Entry# 25117
1,1-Dimethyltetradecyl hydrosulfide #

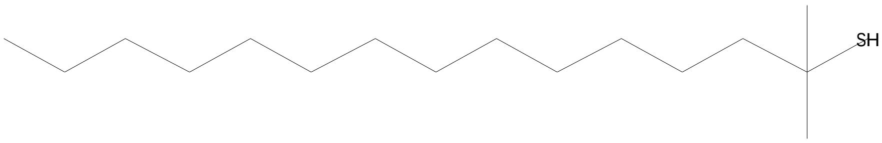


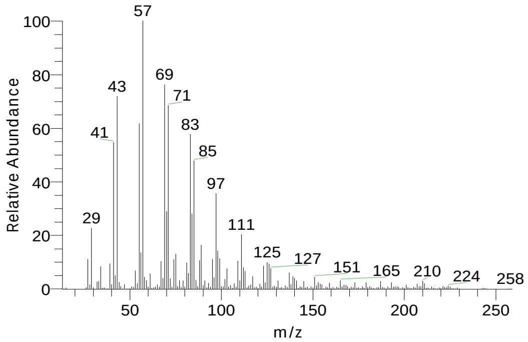


2,2-DIDEUTERO OCTADECANAL
Formula C18H34D2O, MW 270, CAS# 56555-07-8, Entry# 159359

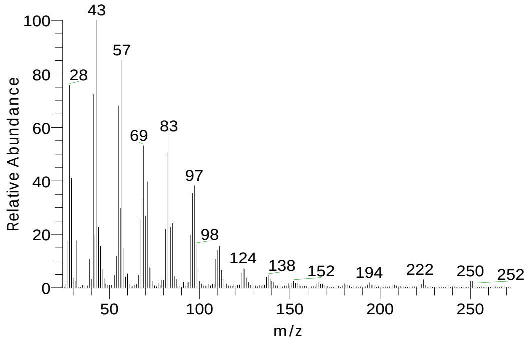


IRON IODIDE COMPLEX I
Formula C26H26FeIN4O4, MW 641, CAS# NA, Entry# 362815

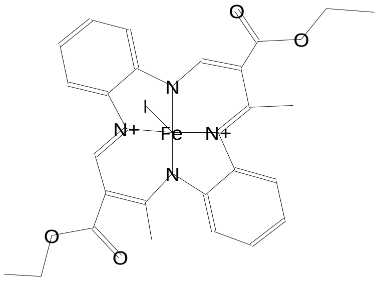


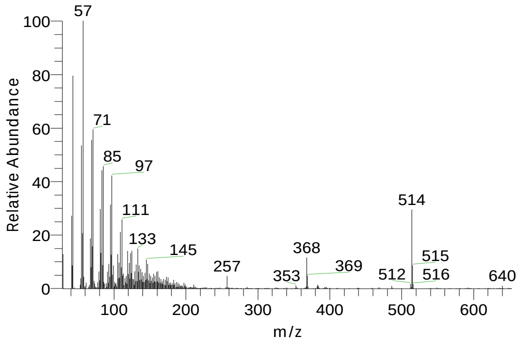

# My GC-MS Report

Clove_SayedMekky_DrSayed #10968 RT: 39.78 AV: 1 NL: 3.79E5
T: + c EI Full ms [50.000-500.000]

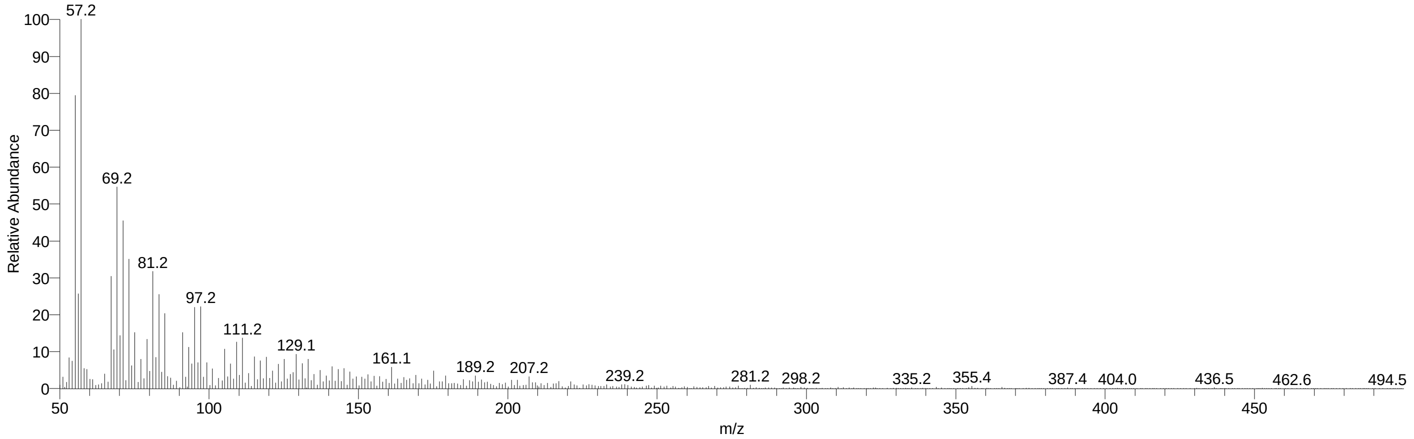


| RT | Compound Name | Area % | MF | Molecular Formula | Molecular Weight | Cas # | Library |
|---|---|---|---|---|---|---|---|
| 39.78 | 1-Heptatriacotanol | 1.02 | 804 | C37H76O | 536 | 105794 -58-9 | mainlib |
| 39.78 | 01297107001 TETRANEURIN - A - DIOL | 1.02 | 796 | C15H20O5 | 280 | NA | WileyRegistry8e |
| 39.78 | Ethyl iso-allocholate | 1.02 | 779 | C26H44O5 | 436 | NA | mainlib |
| 39.78 | ETHYL ISO-ALLOCHOLATE | 1.02 | 779 | C26H44O5 | 436 | NA | WileyRegistry8e |
| 39.78 | ISOCHIAPIN B | 1.02 | 779 | C19H22O6 | 346 | NA | WileyRegistry8e |

| Compound Structure | Hit Spectrum |
|---|---|

1-Heptatriacotanol
Formula C37H76O, MW 536, CAS# 105794-58-9, Entry# 7279
1-Heptatriacontanol #

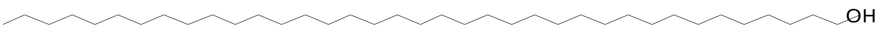


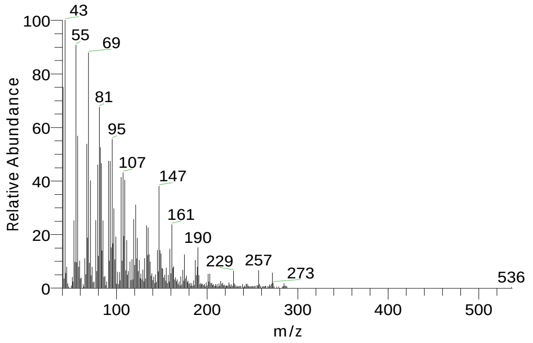


01297107001 TETRANEURIN - A - DIOL
Formula C15H20O5, MW 280, CAS# NA, Entry# 170378

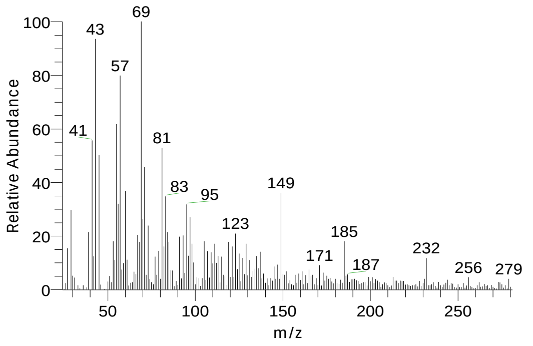

# My GC-MS Report

Compound Structure | Hit Spectrum

Ethyl iso-allocholate
Formula C26H44O5, MW 436, CAS# NA, Entry# 7020
Ethyl 3,7,12-trihydroxycholan-24-oate #

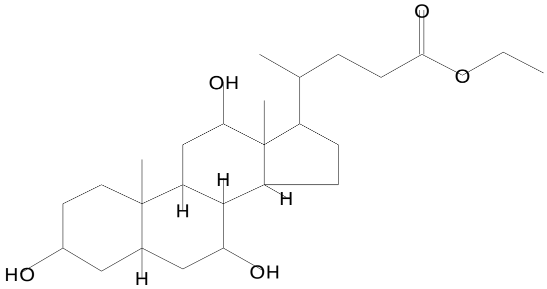


SI 778, RSI 779, mainlib, Entry# 7020, CAS# NA, Ethyl iso-allocholate

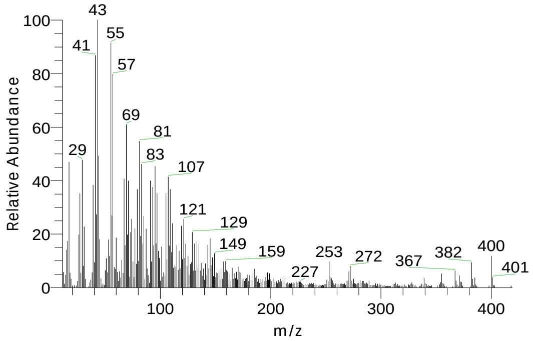


ETHYL ISO-ALLOCHOLATE
Formula C26H44O5, MW 436, CAS# NA, Entry# 270212

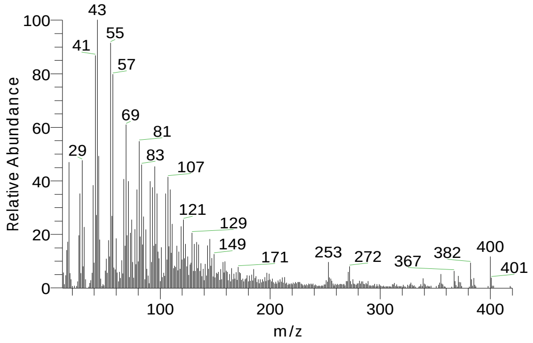


ISOCHIAPIN B
Formula C19H22O6, MW 346, CAS# NA, Entry# 225807

SI 777, RSI 779, WileyRegistry8e, Entry# 225807, CAS# NA, ISOCHIAPIN B

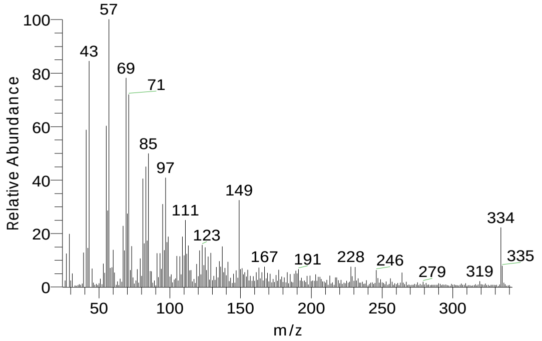

Supplement: Supplementary file 1 [file Data_Sheet_1.ZIP › Editorial 2.PDF]
