# Supplementary material for: Clove-mediated green synthesis of silver nanoparticles and in vitro investigation of their antimicrobial, antioxidant, and anticancer potential
Source: Front Microbiol. 2026 Jul 28;17:1860045. doi: 10.3389/fmicb.2026.1860045 (PMC13457700; doi:10.3389/fmicb.2026.1860045)
Supplement: Supplementary file 1 [file Data_Sheet_1.ZIP › Table 1.DOCX]

**Supplementary File**
